# Urea-Peptide Hybrids as VEGF-A_165_/NRP-1 Complex Inhibitors with Improved Receptor Affinity and Biological Properties

**DOI:** 10.3390/ijms22010072

**Published:** 2020-12-23

**Authors:** Anna K. Puszko, Piotr Sosnowski, Rachel Rignault-Bricard, Olivier Hermine, Gérard Hopfgartner, Karolina Pułka-Ziach, Yves Lepelletier, Aleksandra Misicka

**Affiliations:** 1 Faculty of Chemistry, University of Warsaw, Pasteura 1, 02-093 Warsaw, Poland; karola@chem.uw.edu.pl; 2 Department of Inorganic and Analytical Chemistry, University of Geneva, 24 Quai Ernest Ansermet, CH-1211 Geneva, Switzerland; piotr.sosnowski@unige.ch (P.S.); gerard.hopfgartner@unige.ch (G.H.); 3 Imagine Institute, Université de Paris, 24 boulevard Montparnasse, 75015 Paris, France; rachel.rignault@parisdescartes.fr (R.R.-B.); ohermine@gmail.com (O.H.); y.lepelletier@gmail.com (Y.L.); 4 INSERM UMR 1163, Laboratory of Cellular and Molecular Basis of Normal Hematopoiesis and Hematological Disorders: Therapeutical Implications, 24 Boulevard Montparnasse, 75015 Paris, France; 5 Department of Neuropeptides, Mossakowski Medical Research Centre, Polish Academy of Sciences, Pawinskiego 5, 02-106 Warsaw, Poland

**Keywords:** peptidomimetics, amide bond mimetic, neuropilin-1, VEGF-A_165_, protein–ligand interaction

## Abstract

Neuropilin-1 (NRP-1), the major co-receptor of vascular endothelial growth factor receptor-2 (VEGFR-2), may also independently act with VEGF-A_165_ to stimulate tumour growth and metastasis. Therefore, there is great interest in compounds that can block VEGF-A165/NRP-1 interaction. Peptidomimetic type inhibitors represent a promising strategy in the treatment of NRP-1-related disorders. Here, we present the synthesis, affinity, enzymatic stability, molecular modeling and in vitro binding evaluation of the branched urea–peptide hybrids, based on our previously reported Lys(*h*Arg)-Dab-Oic-Arg active sequence, where the Lys(*h*Arg) branching has been modified by introducing urea units to replace the peptide bond at various positions. One of the resulting hybrids increased the affinity of the compound for NRP-1 more than 10-fold, while simultaneously improving resistance for proteolytic stability in serum. In addition, ligand binding to NRP-1 induced rapid protein stock exocytotic trafficking to the plasma membrane in breast cancer cells. Examined properties characterize this compound as a good candidate for further development of VEGF_165_/NRP-1 inhibitors.

## 1. Introduction

**Figure 1:**
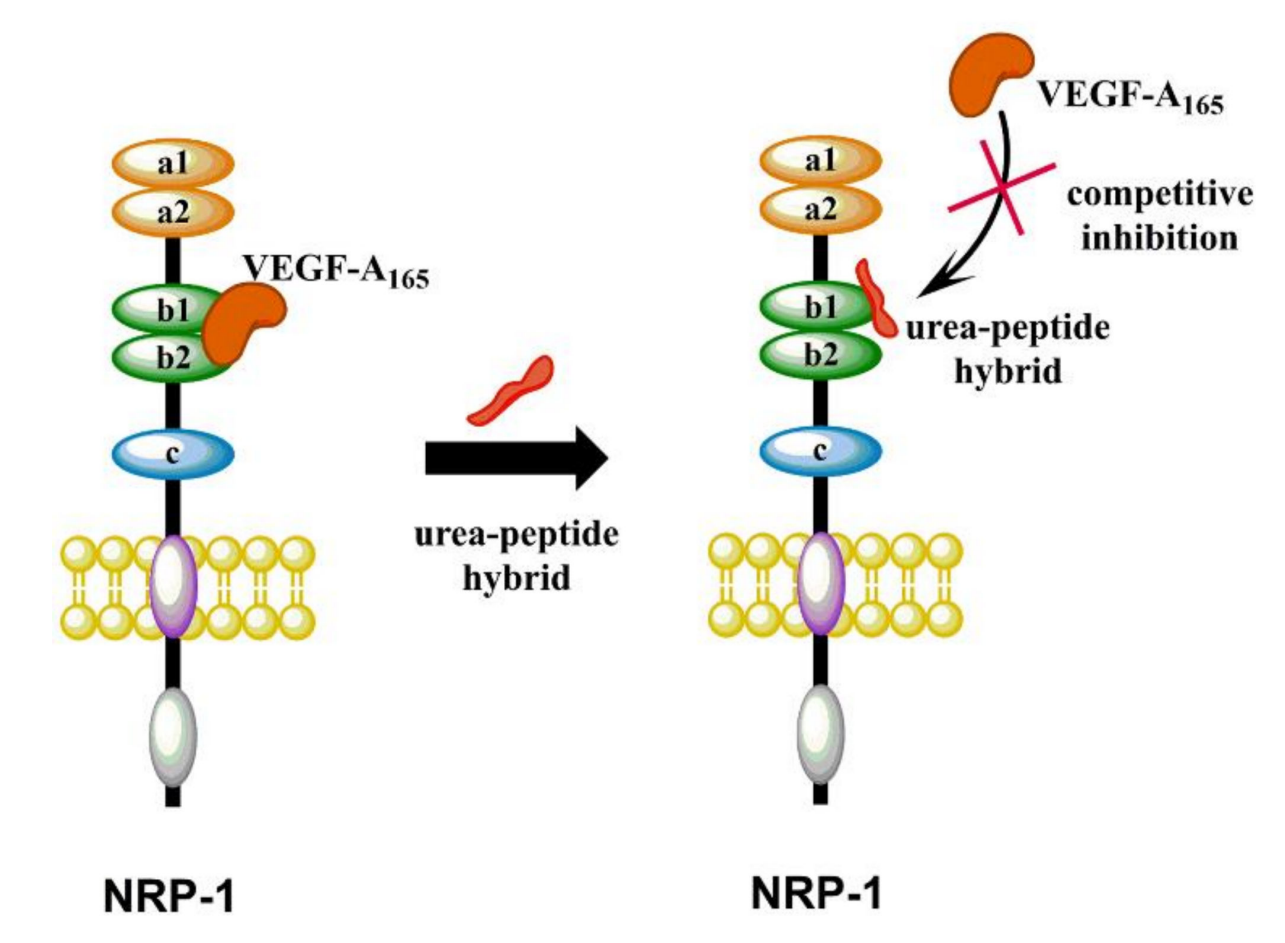
Schematic cartoon representing the VEGF-A_165_/NRP-1 complex formation inhibited by synthesized urea–peptide hybrids.

The most important member from the vascular endothelial growth factors (VEGFs) family is the VEGF-A_165_ isoform, which has been shown to play major roles in physiological and pathological angiogenesis [1,2]. It also affects vascular permeability through binding to type III tyrosine kinase receptors from the vascular endothelial growth factor receptors (VEGF-R) family: VEGF-R1 and VEGF-R2 [1,2,3,4,5]. Another significant receptor for VEGF-A_165_ is neuropilin-1 (NRP-1), expressed on endothelial cells and responsible for enhancing the VEGF-A_165_/VEGF-R2 signaling as a co-receptor, thereby increasing endothelial cell proliferation and migration and promoting angiogenesis [6,7,8,9]. NRP-1 is a transmembrane glycoprotein without catalytic activity. Apart from a role in the formation of new blood vessels, it participates in many signaling pathways, such as regulation of neuronal guidance [10], modulation of the immune response [11,12] and cell migration and survival. Despite the lack of catalytic activity, NRP-1 is considered to be an independent mediator of tumor development and progression, since it is extensively overexpressed in many cancerous cells [13,14,15,16], which is associated with tumor progression, metastasis and poor clinical outcome [17,18,19]. Therefore, the search for compounds that will inhibit the formation of the VEGF-A_165_/NRP-1 complex (Figure 1) may contribute to the development of potential anti-cancer drugs in the future. On the other hand, induction of angiogenesis and thus activation of NRP-1 is a therapeutic hope due to its usefulness in diseases such as coronary artery disease, stroke and impaired wound healing [7,20,21]. Strategies of blocking VEGF-A_165_/NRP-1 complex formation to modulate angiogenesis are currently extensively studied. Designed inhibitors include small molecules [22,23,24,25,26,27,28,29] and linear [30,31,32,33,34,35,36,37,38,39,40,41,42,43,44,45,46] and cyclic [47,48,49,50] peptides or peptidomimetics. 

Peptides are generally selective molecules that bind to specific cell surface receptors and are capable of initiating intracellular effects. Their specificity ensures safety, excellent tolerability and efficacy in therapy [51]. It has been proved that the binding pocket of NRP-1, b1-domain, requires the presence of C-terminal Arg/Lys-Xaa-Xbb-Arg/Lys motif in the ligands [2,35,36,52]. This absolute structural condition is called C-end rule (CendR) [35,36]. The C-terminal sequence of VEGF-A_165_ (CDKPPR) is responsible for growth factor interaction with NRP-1 and follows CendR [52,53,54,55]. CendR motif occurs in the sequences of many active peptides with proved affinity for NRP-1, e.g., A7R [30,32,34,56], tuftsin [31] and penetrating iRGD peptide [57]. Unfortunately, naturally occurring peptides have intrinsic flaws, such as poor chemical and physical stability, short circulating plasma half-lives, fast clearance and low membrane permeability [58,59]. Some of these drawbacks might be successfully resolved through modifications, such as the introduction of unnatural amino acids, amide bond mimetics, cyclisation (including stapling) and clipping of peptide sequences. As described recently in the literature, active pseudopeptides or compounds with peptide bond surrogates are particularly attractive; these include reduced peptide bonds [CH_2_NH] [60,61], ester bonds in depsipeptides [62], thioamides [CSNH] [63,64,65], 1,2,3-triazoles (1,4 or 1,5-disubstituted) [66,67,68] and urea and thiourea bonds [69,70,71,72]. The amide bond surrogates maintain three-dimensional structures similar to those of natural peptides, but may possess different polarity, which could affect the formation of hydrogen bonds or acid-base interactions [73]. Most importantly, pseudopeptides are more resistant to the proteolytic degradation, due to the enzymes not recognizing properly the cleaving site.

We have recently developed short branched peptides with significant inhibitory effects on VEGF-A_165_/NRP-1 complex formation [42,44]. The most promising among them have the Lys(*h*Arg)–Dab–Oic–Arg sequence, where the ε-amino group of Lys forms an amide bond with homoarginine (*h*Arg) carboxyl group [44]. To obtain more stable analogues, the side chain of the Lys residue in the first position was coupled with Arg urea moiety [45]. The resulting compound Lys(Arg^U^)–Dab–Oic–Arg exhibited similar inhibitory activity compared to the parent sequence, but its enzymatic stability was significantly higher. In the presented study, in order to further induce the enzymatic stability and activity of NRP-1 ligands, we designed compounds based on branched Lys(*h*Arg) fragment, which was modified using various urea units. Synthesis, inhibitory activity against the VEGF-A_165_/NRP-1 complex, enzymatic stability, molecular modelling and in vitro analysis of the derivatives with introduced urea bonds are described. This work is the next step in structure–activity relationship (SAR) studies for ligand–NRP-1 interactions and the development of prospective drug candidates.

## 2. Results and Discussion

### 2.1. Design Strategy

**Figure 2:**
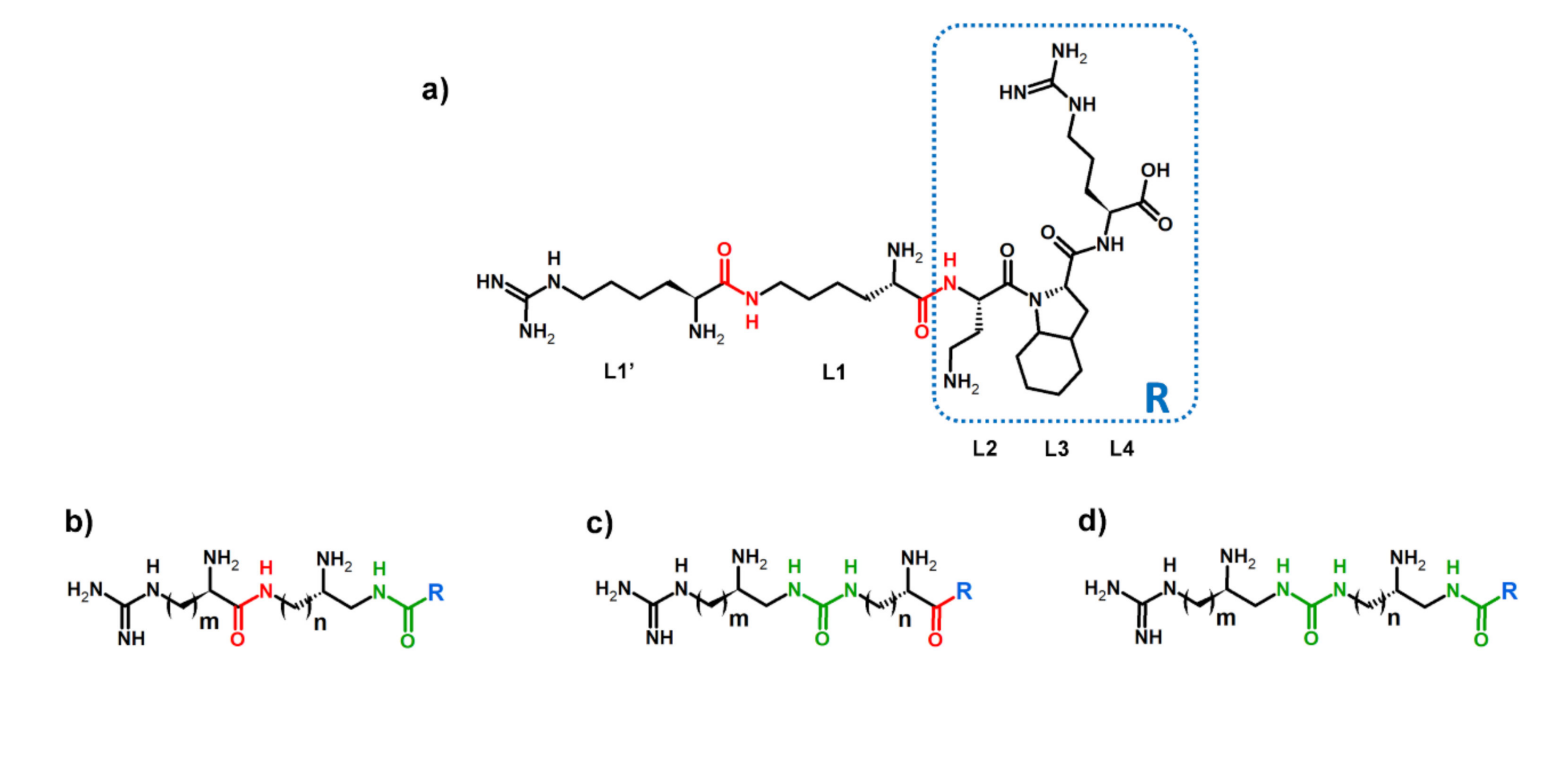
(**a**) Parent sequence with marked amide bonds (red) intended for modification. L denotes the position of the amino acid in the sequence. The general structure of urea–peptide hybrids branching in (**b**) compounds **1**, **2** and **3**; (**c**) compounds **4**, **5** and **6**; and (**d**) compounds **7**, **8** and **9** with urea bonds marked (green).

Identifying sites for the hydrolysis of the compound by proteases is one of the steps for the rational design of peptidomimetics. It can indicate parts of the molecule that need chemical modifications to increase the biological stability of the compound. Our aim was to design enzymatically stable, short and branched peptidomimetics, based on our previously described Lys(*h*Arg)-Dab-Oic-Arg sequence, which exhibits highly competitive inhibition of NRP-1/VEGF-A_165_ complex formation [44]. Lys(*h*Arg) branching is crucial for obtaining high affinity to NRP-1 receptor, but undergoes enzymatic hydrolysis primarily. Considering that a urea moiety might enhance stability and provide additional hydrogen bond donor through an extra –NH– group, which may generate additional interactions sites with target, it could be considered as a beneficial peptide bond surrogate [74]. However, such a replacement might also affect a compound’s inhibitory activity. Our previous studies showed that the substitution of Lys-ε-*h*Arg amide bond by urea group and replacement of *h*Arg with shorter Arg side chain made peptidomimetics more resistant to enzymatic cleavage, but it also affected the affinity for NRP-1. The inhibitory activity of the most potent Lys(*h*Arg)-Dab-Oic-Arg peptide (IC_50_ = 2.3 µM) [44] decreased about two-fold due to introduction of urea bond (IC_50_ = 5.5 μM) [45]. Lower inhibitory activity might be related to N-terminus branching elongation, as urea moiety changed the number of atoms in the chain, thereby suggesting further refinement of Lys(*h*Arg) part. The applied combination of building blocks provides the same branching length as present in the Lys(*h*Arg) fragment of parent sequence but modifies the position of individual residues and the location of the urea bond. Three different strategies were envisioned (Figure 2a) and the following sites were modified: between the carboxylic group of Lys (L1) and α-amino group of Dab in L2 (Figure 2b); between the carboxylic group of *h*Arg (L1′) and ε-amino group of Lys in L1 (Figure 2c); in both sites simultaneously (Figure 2d).

### 2.2. Synthesis

**Scheme 1:**
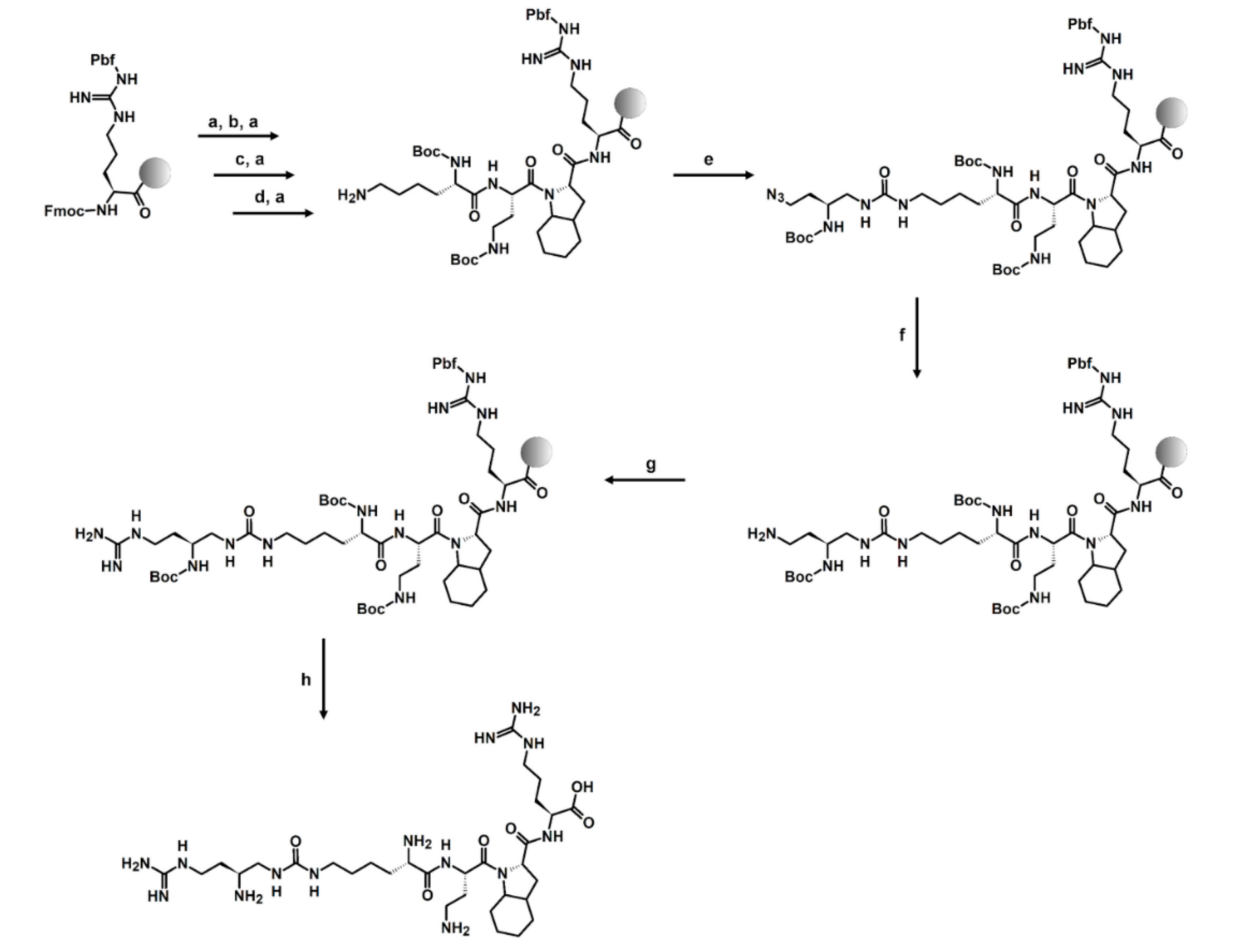
The solid-phase peptide synthesis (SPPS) strategy on the example of urea–peptide hybrid **4**: (**a**) 20% piperidine in DMF; 20 min; (**b**) 2 eq Fmoc-Oic-OH, 2 eq HCTU, 5 eq DIPEA, 90 min; (**c**) 2 eq Fmoc-Dab(Boc)-OH, 2 eq HCTU, 5 eq DIPEA, 90 min; (**d**) 2 eq Boc-Lys(Fmoc)-OH, 2 eq HCTU, 5 eq DIPEA, 90 min; (**e**) 1.5 eq Boc-Dab(N_3_) BB, 3 eq DIPEA, 50 W, 60 °C, 2 × 15 min; (**f**) 10 eq 1 M PMe_3_/THF, 7:3 1,4-dioxane:H_2_O, 50 W, 60 °C, 2 × 30 min; (**g**) 5 eq DMPCN, 19.5 eq DIPEA, 7 days; (**h**) 95:2.5:2.5 TFA:H_2_O:TIS, 3 h.

To introduce urea units into the peptidomimetic sequence, Boc-protected succinimidyl carbamate building blocks with side chain amino groups masked as azide groups or succinimidyl carbamate building blocks with α-amino groups masked as azide groups were used. The general synthesis of such activated building blocks is well described in the literature [75], and a simplified pathway with NMR data is presented in Supplementary Materials (Scheme S1, Figures S1–S5). The synthesis of urea–peptide hybrids was carried out following the Fmoc chemistry [76] using O-(1H-6-chlorobenzotriazole-1-yl)-1,1,3,3-tetramethyluronium hexafluorophosphate (HCTU) [77] as a coupling reagent and *N*,*N*-diisopropylethylamine (DIPEA) as a base. Active carbamate building block coupling and azide reduction were supported by microwave irradiation as previously described [45,75]. Guanidinylation reaction of side chain amino group in L1’ position was performed for 7 days using 3,5-dimethylpyrazole-1-carboxamidine nitrate (DMPCN) [78]. The synthesis pathway can be found in Scheme 1. The remaining compounds were synthesized in an analogous manner using sequence-appropriate building blocks and by carrying out, respectively, coupling and amine deprotection reactions for amino acids or coupling and azide reduction to amine for urea units. When Arg or *h*Arg residues were incorporated in L1’, the guanidinylation step was omitted. The structures of all obtained compounds were confirmed by HRMS and MS/MS fragmentation present in Supplementary Materials (Tables S1–S3, Figures S6–S15).

### 2.3. Receptor Binding Studies

Inhibitory activity of urea–peptide hybrids against VEGF-A_165_/NRP-1 complex formation was assessed with a screening ELISA-like assay. NRP-1 was immobilized on microplate wells by simple adsorption and urea–peptide hybrids competed for NRP-1 with biotinylated VEGF-A_165_ (bt-VEGF-A_165_). The amount of receptor bound bt-VEGF-A_165_ was visualized by its interaction with the streptavidin–horseradish peroxidase conjugate (strep-HRP) and the chemiluminescent substrate. The first analyzed group of compounds were analogues **1**–**3** with the urea bond between L1 and L2. Obtained results show that this modification does not affect binding of hybrids to the receptor significantly (IC_50_ = 1.2–2.2 μM), compared to the parent peptide (IC_50_ = 2.3 μM) (Table 1). The best hybrid in this group was compound **3**, which had Lys^U^ (gDab) branching (where “g” corresponds to guanidinium group), but its IC_50_ value decreased only 2-fold. Therefore, we assume that no additional interactions between urea bond and the surface of the protein occurred. Similarly, no significant change of affinity was observed for third group of compounds with two urea bonds in branching. IC_50_ values for hybrids **7**–**9** were between 1.8–2.1 μM. The most interesting group proved to be urea–peptide hybrids **4**–**6** with modification between L1 and L1′, where inhibitory activity increased 5 to 12-fold. Overall, the most promising among all analogues is hybrid **6** with IC_50_ = 0.19 µM. Our previous molecular modelling studies of branched peptides, with structures similar to the parent sequence, showed that the branching might lay over the receptor surface and interact with protein via the guanidinium group and/or α-amino group of *h*Arg [42]. However, the lack of the crystal structure of the branched peptide/receptor complex significantly hinders SAR studies and prediction of the effects of modifications to ligand–receptor interactions. In the case of urea–peptide hybrids, the urea bond might be capable of forming additional hydrogen bonds with protein residues on the surface of NRP-1. The affinity of compounds may conceivably be affected by the colocation of the urea bond and the free amino group of *h*Arg in the branching. In addition, for compounds **4**–**6**, we can observe that decreasing the distance between the free amino group in the main chain and the urea bond in the branching has a positive effect on compounds’ affinity for NRP-1. 

Binding selectivity is one of the crucial requirements on the path toward development of a non-hazardous therapeutics, as nonspecific binding generates a risk of causing unwanted side effects. However, absolute selectivity for a single protein is most often unreachable. An appropriate selectivity profile can lead to better drug properties by targeting multiple proteins, eventually leading to further benefits [79,80].

The most potent urea–peptide hybrid, **6**, was subjected to the analysis of selectivity to angiogenesis associated receptors, NRP-2, VEGF-R1 and VEGF-R2, which form a complex with VEGF-A_165_. Amino acid sequence homology for primary structures of NRP-1 and NRP-2 is 44% [81,82]. Structural studies have shown that both receptors bind VEGF-A_165_ using a conserved binding pocket core, formed by the b1 subdomain loops [83]. VEGF-A_165_ interacts also with VEGF-R1 and R2 receptors by different fragments encoded by exons 1–5 [84,85]. Affinity studies were done using modified ELISA-like assay, performed in the same manner as affinity tests for NRP-1 after optimization of method for individual protein. Obtained results show that urea–peptide hybrid **6** exhibits high inhibitory activity with the IC_50_ = 0.48 µM against VEGF-A_165_/NRP-2 complex formation (Table 2). However, this value is more than two times higher compared to VEGF-A_165_/NRP-1 complex inhibition. At the same time, our data suggest no affinity for both VEGF-Rs.

### 2.4. 2D NMR and Computational Study

**Figure 3:**
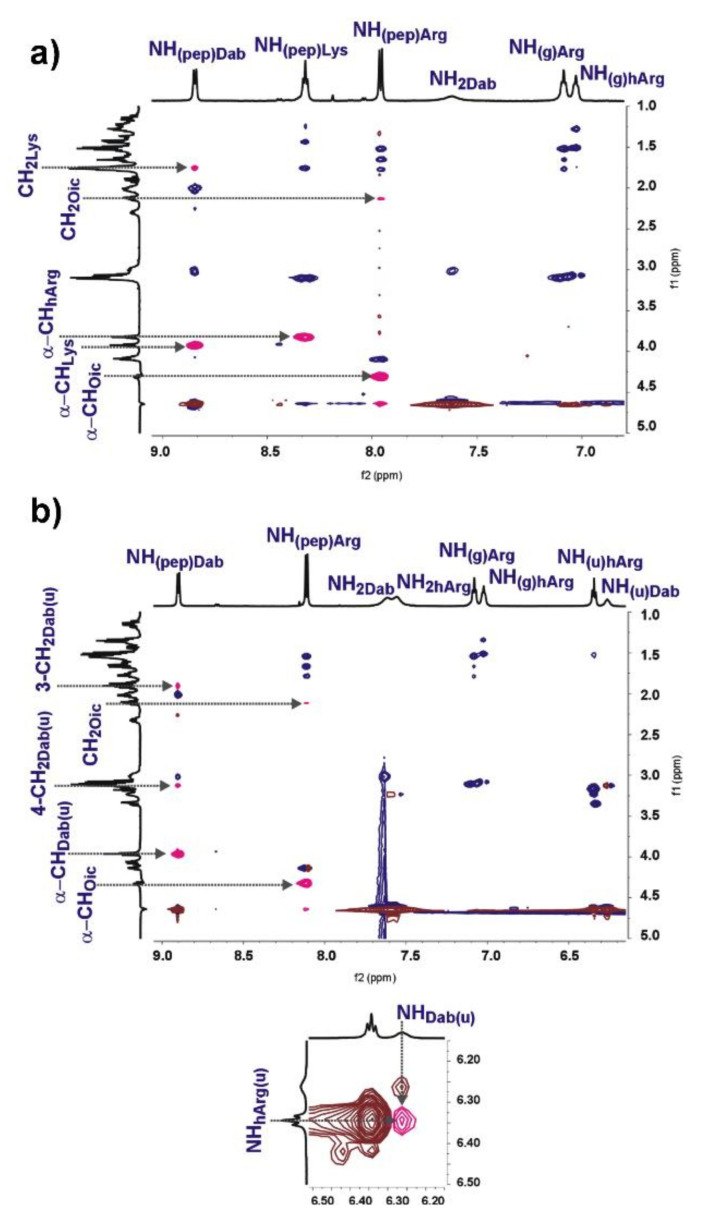
Part of NH/aliphatic region of ROESY spectra: NOEs between protons of different residues are marked with pink; (**a**) parent peptide; (**b**) hybrid **6** and NOE of NH/NH urea protons.

2D NMR spectra were recorded to get an insight in to the structure of parent peptide and urea–peptide hybrid **6** in solution. Both compounds were dissolved in DPBS buffer with 10% addition of D_2_O. Signals were assigned based on TOCSY, COSY and HSQC spectra (Figures S46–S50 and S52–S56). The analysis of ROESY spectra (Figure 3) indicated that both compounds exhibited very similar patterns of NOE connectivities. Most of ROESY signals overlapped perfectly well with the correlations observed at TOCSY (Figures S51 and S57); therefore, they reflected interaction only within one residue. For both compounds, we observed strong NOEs between sequential α-CH/NHpep protons and weak NOEs between some NHpep and CH_2_ protons of neighboring amino acid residues in the branching (Figure 2). Any medium-range NOEs were not detected. All these observations indicated that parent peptide and hybrid **6** adopted quite similar extended and unstable conformations in solution [86]. It is also interesting to mention that NOE connectivity between protons of urea moiety in hybrid **6** was observed (Figure 3b), which may suggest trans/trans conformation of this urea group. NMR studies of the structure of the parent peptide and hybrid **6** in solution did not provide any explanation about differences in inhibitory activity against VEGF-A_165_/NRP-1 complex formation.

In order to identify possible biding sites of parent peptide Lys (*h*Arg)Dab-Oic-Arg and urea–peptide hybrid **6**, we prepared complexes of both compounds with NRP-1 receptor and performed molecular dynamics simulations. Using the available 2ORZ crystallographic structure [33], where the C-terminal Arg residue of short peptide tuftsin is present in binding pocket of NRP-1, we superimposed both compounds’ Arg residue on its counterpart in tuftsin. The remaining chain was set to extend outside of the protein, after which system was minimized and equilibrated. From this point, simulations were run independently in six repetitions for 200 ns each. Detailed information with graphs presenting evolution of the distance in time can be found in Supplementary Materials (Figures S58–S71). Many short-lived contacts could be observed; thus, it is impossible to indicate a single binding pose for in silico tested molecules, but the prevalence of selected interactions may explain the hybrid **6** increase of affinity. By comparing the simulation outcome for both molecules, we can observe a similar behavior of Arg residue in NRP-1 binding pocket to previously described results [41,42]. 

**Figure 4:**
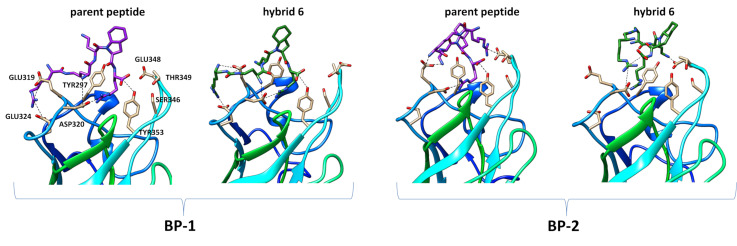
Molecular dynamics snapshots representing examples of binding poses between NRP-1 protein residues (PDB 2ORZ) and ligand functional groups for parent peptide and hybrid **6**.

A summary of the observed interactions of both molecules in each run can be found in Supplementary Materials (Figure S72). A visual representation of binding poses is presented in Figure 4 with snapshots extracted from simulations. The most notable difference is in interactions of *h*Arg free amino group and urea moiety. In majorly present binding pose 1 (BP-1) both molecules could be described as “spread”, similar to their 2D drawing. For the parent peptide, the guanidinium group of *h*Arg interacts with Glu319 and Glu324. N-terminal amine interacts through hydrogen bonding with Asp320, and other positively charged groups are directed outside of the protein (Figure 4). In the case of hybrid **6**, the urea moiety forms hydrogen bonds with Glu319 and “wraps” the molecule around this region, as at the same time interactions between free amino groups of *h*Arg^U^ with Glu319 and Dab (L1) with Glu319 or Asp320 are also observed. The guanidinium group of *h*Arg^U^ either forms interactions with nearby Glu324 or is exposed to solvent (Figure 4).

In the much less frequent binding pose 2 (BP-2), the molecule is “twisted” in a manner such that the guanidinium group of *h*Arg or *h*Arg^U^ moiety is reaching the Glu348 (parent peptide) or Asp320 (hybrid **6**) while also forming the intra molecular interactions between carboxyl group of C-terminal Arg. This also promotes the formation of hydrogen bond between free amino group of Dab side chain and Glu319 (parent peptide) or Glu348 (hybrid **6**). BP-2 was significantly present in 2 out of 6 simulations for parent peptide (*h*Arg-Glu348 contact for more than 50% simulation time) and 1 out of 6 simulations for hybrid **6** (*h*Arg^U^-Arg contact for more than 50% simulation time).

### 2.5. In Vitro Enzymatic Stability

**Figure 5:**
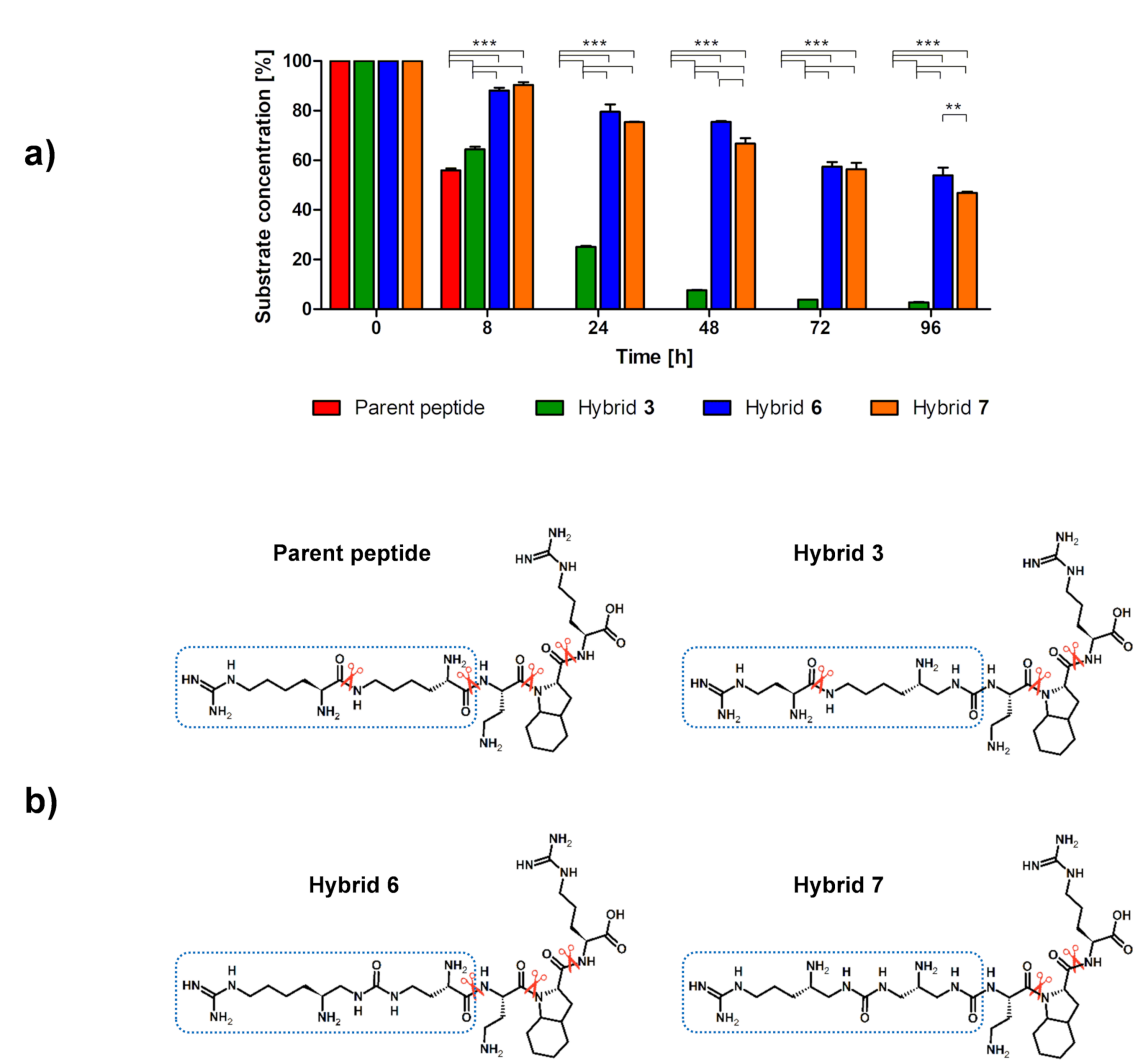
(**a**) Comparison of the in vitro metabolic stability of parent peptide and its urea–peptide hybrids **3**, **6** and **7**. Results are presented as the means ± SEMs of three independent experiments on LCMS (*N* = 3). In order to show significant differences in the concentration of intact substrate at each time interval depending on the sequence of the hybrid, a two-way ANOVA with Bonferroni’s post-tests was done (** *p* < 0.01, *** *p* < 0.001). (**b**) Potential bonds cleaved by enzymes (scissors show identified proteases cleavage sites).

Rapid renal clearance and enzymatic degradation are classified as most important disadvantages of peptides as drugs. Covalent linkage to plasma proteins or chemical modifications, including amide bond modifications, might improve their half-lives [87]. We performed proteolytic stability studies in human blood serum to investigate whether the introduction of urea bonds affected the half-lives of compounds from each of synthesized group of hybrids. Compounds **3**, **6** and **7** were incubated with human serum at 37 °C. Samples were taken at different time intervals and analyzed using LC-SWATH-MS [88,89,90]. Identified cleavage sites were compared with the degradation of the parent peptide, which exhibited a half-life of 8 h. Hybrids **6** and **7** were stable toward enzymatic degradation, with 50% of each compound still being intact after 96 h (Figure 5). These analogues exhibit improved stability compared to the parent Lys (*h*Arg)-Dab-Oic-Arg sequence. In the case of hybrid **3**, degradation was significantly faster, and after 24 h only ~25% of initial substrate concentration remained in the sample. However, it was still more stable compared to the parent peptide. Overall, it can be concluded that the substitution of an amide bond with a urea bond has a positive effect on the proteolytic stability of the examined inhibitors.

Analysis of the metabolites of urea–peptide hybrids by LC-MS, predicted by their theoretical masses, shows that urea bonds remained intact within the monitored time of the experiment (Tables S5–S8, Figures S17–S44). An example is the Dab-Oic-Arg (L2-L3-L4) metabolite, which is easily detected in the degradation of the parent peptide and hybrid **6**, but non-existent for hybrid **3** and **7**, where the peptide bond of AA-Dab (L1–L2) was substituted with its urea analogue. The results suggest that the bond between the side chain of the unit in L1 and L1′, which lengthens the branching, may play a significant role not only in the interaction with the receptor, but also in the stability of the branched compounds. Modification of this bond to obtain increase in enzymatic stability in serum was therefore justified.

### 2.6. Binding Assays on Cells

One of the methods applied for the detection of a compound, considering biological assays on living cells, is the application of a fluorescently tagged analogue for flow cytometry analysis. In order to carry out this experiment, we synthesized compound **10**, which is a hybrid of **6** labeled with 5/6-carboxyfluorescein (5/6-FAM) at N-terminus (5/6-FAM-Dab(*h*Arg^U^)-Dab-Oic-Arg). This molecule was first used to examine inhibitory activity of tagged hybrid against VEGF-A_165_/NRP-1 complex formation using ELISA-like assay. The results show a significant increase of IC_50_ after labeling (IC_50_ = 4.04 µM) compared to unmodified compound (IC_50_ = 0.19 µM), but it must be noted that the 5/6-FAM tag may influence peptide affinity for NRP-1, e.g., due to its size. The IC_50_ value should be sufficient to prove compound interaction with NRP-1 (Table S4 in Supplementary Materials).

**Figure 6:**
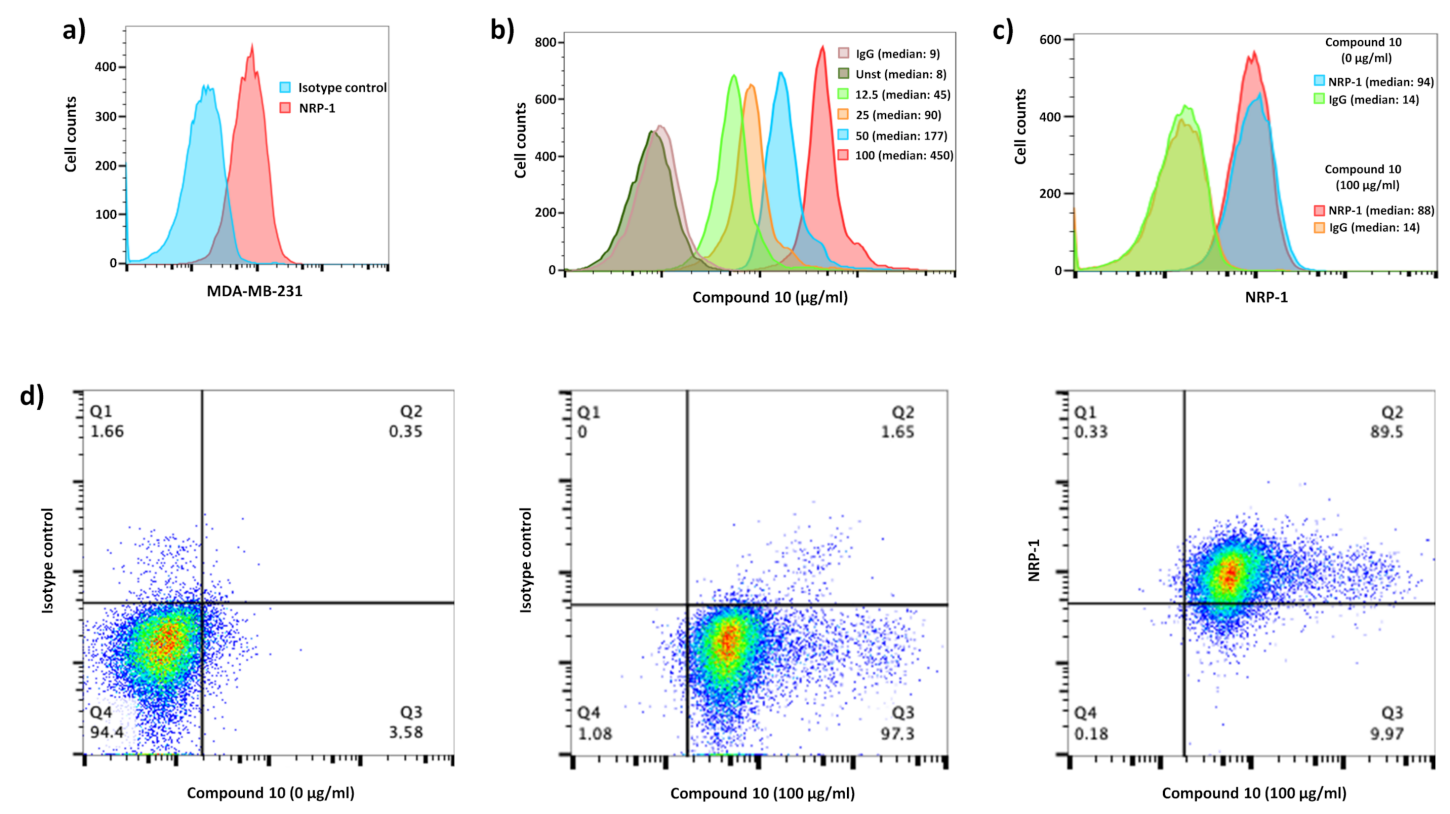
Compound **10** binds to NRP-1-positive MDA-MB-231 breast cancer cells. (**a**) Histogram shows NRP-1 level expression (red) compared to irrelevant control IgG staining (blue) on MDA-MB-231. (**b**) 5/6-FAM coupled with hybrid **6** (compound **10**) was used at 100 (red), 50 (blue), 25 (orange) and 12.5 (green) µg/mL to follow its positive binding on MDA-MB-231 compared to unstained (dark green) and FITC-irrelevant IgG (pink) as controls. Median fluorescence intensity is shown for each staining. (**c**) The histogram shows the uncompetitive and steric access for the NRP-1 staining in the presence (blue) of compound **10** compared to its absence (red). Both NRP-1 stainings are compared to irrelevant control IgG staining (green and orange respectively). Median fluorescence intensity is shown for each staining. (**d**) Dot plots show cells expressing NRP-1, which bound fluorescent compound **10**, compared to unstained conditions without compound **10** or with irrelevant isotype control for NRP-1 staining.

VEGF-A_165_ binds to cell-surface NRP-1 on endothelial cells, effectively reducing surface levels of this receptor, promoting complex internalization and possibly inducing biological effects [91]. Based on this mechanism, peptide carriers for selective drug delivery, such as iRGD peptides, have been recently disclosed [57]. The iRGDs are used to selectively address potential therapeutic agents, e.g., doxorubicin or siRNAs inside malignant cells. We tested our tagged urea–peptide hybrid’s ability to bind human NRP-1-positive breast cancer cells (MDA-MB-231), which expressed large amount of NRP-1 at the cell surface (Figure 6a). A broad range of concentrations between 0 to 100 µg/mL of compound **10** was used to show its binding capacity on cells using fluorescence-activated cell sorting (FACS). As shown in Figure 6b, binding on cells was dose-dependent. To follow the impact of the ligation of this peptidomimetic on NRP-1 expression, we verified whether hybrid **10** binding interferes with the antibody used to detect NRP-1. As we can observe in Figure 6c, the presence of compound **10** did not interfere with the NRP-1 detection (94 vs. 88 of median fluorescence intensity, respectively), allowing us the study of NRP-1 regulation in presence of these inhibitors. Finally, we performed co-staining (hybrid **10** and NRP-1 staining) to determine the ability (percentage) of NRP-1-positive MDA-MB-231 to bind compound **10**. All cells were able to bind our labeled hybrid and then all of these cells expressed NRP-1 (Figure 6d middle and right panels, respectively). In conclusion, we could follow the expression of NRP-1 by MDA-M-231 exposed to compound **10** using co-staining.

**Figure 7:**
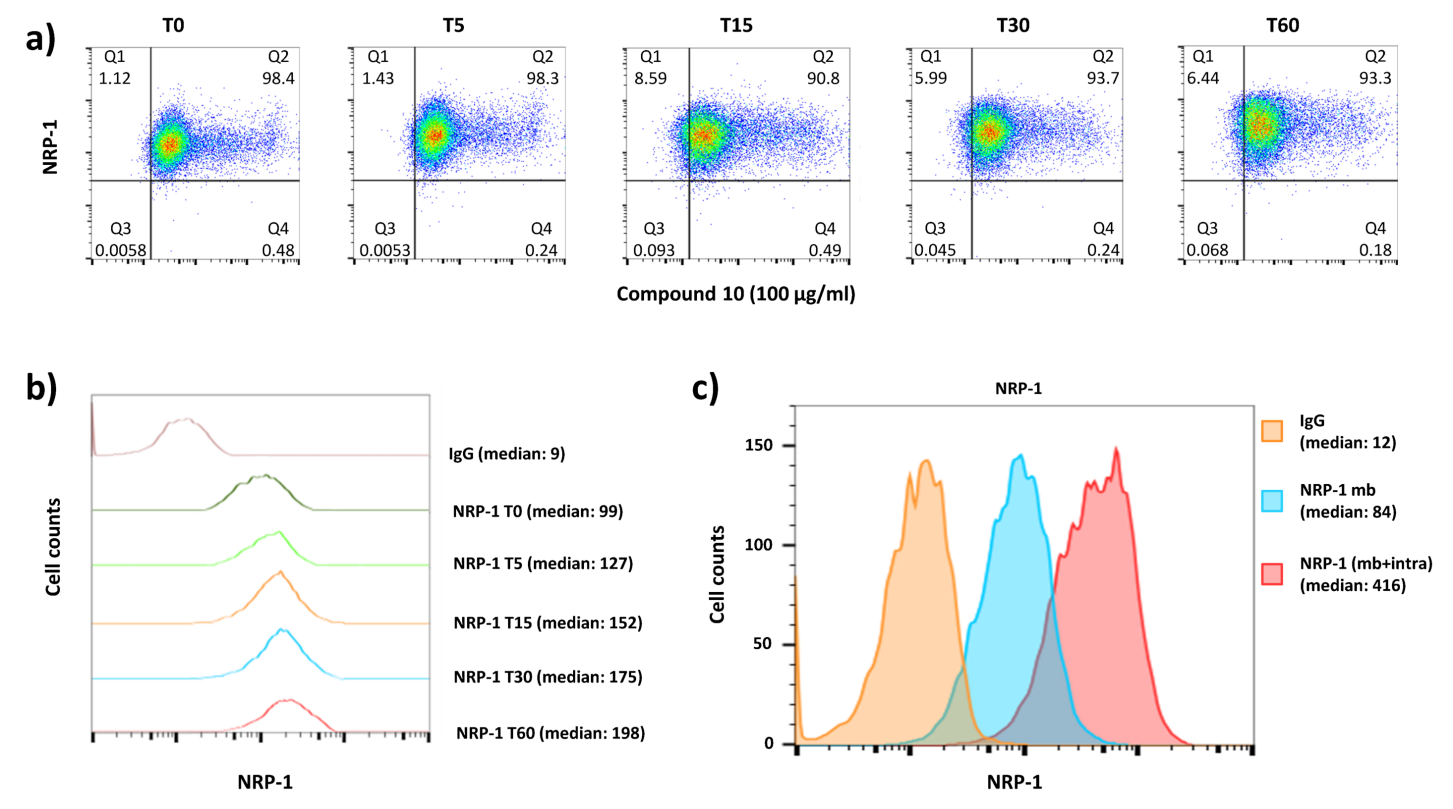
Urea–peptide hybrid **10** modify NRP-1 level expression on MDA-MB-231 breast cancer cells. (**a**) Dot plots show the percentages of NRP-1-positive cells that bind the fluorescent peptide during 37 °C exposure at different times (T = 0 to T = 60 min). (**b**) The histogram shows the NRP-1 level expression modification on cells exposed to compound **10** (100 µg/mL) after 60 (red), 30 (blue), 15 (orange) and 5 (green) min at 37 °C compared to level expression at 4 °C (dark green) and to irrelevant control IgG staining (pink). Median fluorescence intensities of NRP-1 staining are shown for each condition. (**c**) The level expression of NRP-1 at the cell membrane (mb, blue histogram) is overlain on the membrane and intracytoplasmic NRP-1 expression (mb + intra, red histogram), and compared to the irrelevant IgG isotype staining (orange histogram). Median fluorescence intensity of NRP-1 staining is indicated for each histogram.

Usually, in the presence of its natural ligand, a transmembrane receptor at the cell surface may induce signaling, internalization of complex and recycling at the cell surface. In contrast, some drugs, e.g., blocking antibodies, may induce internalization of antibody-receptor complex and receptor recycling or internalization with an intracellular sequestration, as we previously showed in anti-human transferrin receptor (anti-Tfr) therapy development [92]. Thus, to identify the mechanism of action of the hybrid **10** on the NRP-1 expression on cells, we followed its expression after a time course exposure of cells at 37 °C. Independently of time exposure, the results prove the presence of compound **10** bound with cells (positive from 5/6-FAM-labeled peptide) with no downmodulation of NRP-1 expression (Figure 7a), as we observed strong signal from the stained receptor at each time interval. This might suggest the incapacity of hybrid **10** to induced downmodulation of NRP-1 by cells or its internalization, since only NRP-1 cell surface expression was followed. This intriguing result focused our attention on tracking the level of expression intensity of NRP-1 on the surfaced of MDA-MB-231 cells exposed on compound **10**. Surprisingly, the NRP-1 level expression, in a time-dependent manner, was rapidly increased at the cell surface. The median fluorescent intensity after 1 h (T60) of cell exposure on hybrid 10 was 1.5-fold higher than after 5 min of incubation (Figure 7b). This upregulation of NRP-1 expression could not be explained by a neo-synthesis of NRP-1 from mRNA, which requires more time, but only by the induction of endogenous stock trafficking of intracytoplasmic NRP-1 reserve toward the cell surface. This hypothesis is strengthened here, since MDA-MB-231 expressed a large amount of NRP-1 at the cell surface, but also had plenty of the cytoplasmic counterpart, reaching a maximum of median fluorescence intensity higher than the restricted level of NRP-1 expression at the surface (Figure 7c). 

We believe there are two possibilities that could explain this phenomenon. One is that hybrid/NRP-1 complex is rapidly internalized into the cell and leads to deprivation of NRP-1 on the cell surface, which is followed by induced NRP-1 transport to the cell membrane. Another one is that inhibition of VEGF-A_165_/NRP-1 complex formation is blocked on the cell surface by hybrid, which causes VEGF-A_165_ deprivation of cells (cells are unable to bind VEGF-A_165_ ligand) and induces rapid NRP-1 stock trafficking to restore VEGF-A_165_ signaling pathways. In both cases, the lack of biochemical signaling is compensated by swift mobilization of NRP-1 intracellular counterpart stock. The process of externalization of an intracellular vesicular pool of receptors was previously observed for other receptors [93].

## 3. Materials and Methods

### 3.1. Materials

Unless otherwise specified, reagents were obtained from commercial sources. LC-MS grade water was purchased from Huberlab (Aesch, Switzerland); methanol and acetonitrile (both HPLC gradient grade) were purchased from VWR (Darmstadt, Germany). Ammonium formate and formic acid were purchased from Sigma-Aldrich (Buchs, Switzerland). Other solvents and reagents were purchased from Merck (Darmstadt, Germany). Fmoc-Arg(Pbf) Wang resin was obtained from Activotec (Cambridge, UK). Amino acids and coupling reagents were purchased from Iris Biotech (Marktredwitz, Germany). Recombinant human receptors and biotinylated human VEGF-A_165_ were purchased from R&D Systems (Minneapolis, MN, USA). Chemiluminescent, streptavidin-horseradish peroxidase conjugate and DPBS were obtained from Thermo Scientific (Waltham, MA, USA).

### 3.2. Succinimidyl Carbamate Building Blocks Synthesis

Activated monomers were prepared using a previously described procedure [75]. Detailed NMR and HRMS data can be found in Supplementary Materials (Figures S1–S5).

Urea–peptide hybrids’ synthesis. Synthesis of urea–peptide hybrids was done manually on the pre-loaded Fmoc-Arg(Pbf) Wang resin with capacity 0.32 mmol/g (0.3 g) following the standard Fmoc chemistry. Coupling of 2 eq protected amino acids (0.19 mmol) was done using 2 eq HCTU (79 mg, 0.19 mmol) and 5 eq DIPEA (81 µL, 0.48 mmol) in DMF (3 mL). For the deprotection step, 20% piperidine in DMF was used. Coupling of protected succinimidyl carbamate building blocks was done following previously reported procedures [75]: 1.5 eq of building block (0.14 mmole) was dissolved in 3 mL of DMF with the addition of 2.5 eq of DIPEA (41 μL, 0.24 mmol). The synthesis was supported by microwave irradiation (60 °C, 50 W, 2 × 15 min). The resin was filtered and washed with DMF (4 × 3 mL). The reduction of azide group was performed with 1M Pme_3_ solution in THF (10 eq relative to the resin loading) in a mixture of 1,4-dioxane:H_2_O (3 mL, 7:3, *v*:*v*) under the microwave irradiation (60 °C, 50 W, 2 × 30 min). After the reaction, the resin was filtered and washed with 1,4-dioxane:H_2_O (1 × 3 mL) and DMF (4 × 3 mL). Guanidinylation reaction was carried out using 5 eq of 3,5-dimethylpyrazole-1-carboxamidine nitrate (DMPCN, 96 mg, 0.48 mmol) dissolved in 3 mL of DMF with 19.5 eq of DIPEA (320 μL, 1.9 mmol) for 7 days. 5/6-FAM was coupled using the same equivalents of reagents for amino acids, but reaction was carried out overnight in darkness. 

After the synthesis, the resin was dried and final compounds were cleaved from the resin using 5 mL TFA:H_2_O:TIS (95:2.5:2.5, *v*:*v*:*v*) for 3 h and then precipitated by dropwise addition into a cold Et_2_O. Crude peptides were collected by centrifugation and purified by preparative RP-HPLC (Duisburg, Germany) on a C12 column (Torrance, CA, USA) with H_2_O/ACN gradient containing 0.1% TFA. Pure compounds were analyzed with the Shimadzu Prominence analytical HPLC system (Duisburg, Germany). Molecular weight and elemental composition were confirmed using a TripleTOF 6600 mass spectrometer (Sciex, Concord, Ontario, Canada). Detailed RP-HPLC, HRMS and MS/MS data can be found in Supplementary Materials (Figures S6–S15).

### 3.3. Competitive Receptor Binding Assays

This method was previously described for NRP-1 [44,45,46]. Briefly, overnight coating at 4 °C of the flat bottom surface of a 96-well plate with 100 μL (200 ng/well) recombinant human NRP-1, NRP-2, VEGFR-1 or VEGFR-2 was followed by non-specific interactions blocking using 0.5% bovine serum albumin (BSA) in PBS. Next, 50 µL of urea–peptide hybrid in PBS and 50 µL (400 ng/mL) of human (bt)-VEGF-A_165_ in PBS containing 4 µg/mL of heparin were added respectively. Plates were incubated for 2 h at RT, and then washed and treated with streptavidin-horseradish peroxidase conjugate in PBS (1:8000). Next, 100 µL chemiluminescent substrate was added, and luminescence was quantified using Tecan Infinite F200Pro microplate reader (Männedorf, Switzerland). In a positive control (P) only (bt)-VEGF-A_165_ was present in wells. Not coated by receptor wells were treated as a negative control (N). Percentages of inhibition were calculated by the following formula:

100% − [[(S − N)/(P − N)]∙100%](1)

where S is the signal intensity measured in wells with urea–peptide hybrid. The IC_50_ of each urea–peptide hybrid was calculated using the nonlinear regression function with GraphPad Prism Version-5.01 (GraphPad Software, San Diego, CA, USA). Data are presented as log (inhibitor) versus normalized response-variable slope (Figure S16). Data are the means ± SEMs of two or three independent experiments, each performed in triplicate. 

### 3.4. 2D NMR Spectroscopy

2D NMR spectra of parent peptide and hybrid **6** were recorded on Agilent DD2 600 MHz spectrometer. The compounds were dissolved in 9:1 DPBS buffer:D_2_O at a concentration of around 10 mg/mL. 1D spectra were recorded with a double pulsed field gradient spin echo (DPFGSE) water signal suppression sequence. Sequences and parameters for 2D spectra were as follows: TOCSY DPFGSE water signal suppression—512 × 256 time domain complex points zero-filled to 2048 × 1024 complex points, and apodized by cosine square function in both dimensions; spectral width 6 kHz in both dimensions; number of scans 16 and mixing time 65 ms. ROESY DPFGSE water signal suppression—512 × 256 time domain complex points zero-filled to 2048 × 1024 complex points, and apodized by cosine square function in both dimensions; spectral width 6 kHz in both dimensions; number of scans 48 and mixing time 200 ms. 13C-1H HSQC—1442 × 400 time domain complex points zero-filled to 2048 × 1024 complex points, and apodized by cosine square function in both dimensions; spectral width 9.6 × 25.6 kHz in F2 and F1, respectively; number of was scans 8. COSY water signal suppression using presaturation—900 × 256 time domain complex points zero-filled to 2048 × 2048 complex points, and apodized by cosine square function on both dimensions; spectral width 6 kHz in both dimensions; number of scans 16.

### 3.5. Molecular Dynamic Studies

Ligand–protein interactions were studied by molecular dynamics (MD) in GROMACS 2018.2 [94]. Input structures were prepared by superposing C-terminal Arg part of studied compounds, on the C-terminal Arg of tuftsin (TKPR) found in 2ORZ crystallographic structure of NRP-1 tuftsin complex. CHARMM-GUI service [95] was used to solvate the complexes (rectangular waterbox, TIP3 waters, almost 48,000 water molecules). In total, 0.154 M of Na^+^ and Cl^-^ ions was added to neutralize the system. The protonation states in ligands and in the protein were set as assumed in pH 7. CHARMM36 force field was used [96]. Unnatural amino acids topologies and parameters were compiled either from existing CHARMM36 values or taken from SwissSideChain [97]. The systems were subject to minimization and NVT equilibration. The production step (NPT ensemble, T ¼ 303.15 K, integration step ¼ 2 fs, cut-off scheme Verlet, Nose-Hoover thermostat, Parrinello-Rahman barostat, LINCS H-bonds constraints) followed. The production lasted 200 ns and was repeated six times (each time from the same starting point) giving 1200 ns for every compound in total. Analysis and visualization of results were performed in Gromacs and UCSF Chimera [98].

### 3.6. Blood Collection and Serum Preparation

The study was conducted in full accordance with ethical principles in accordance with the ICH E6 (R2) Guideline for Good Clinical Practice and the Code of Good Customs in Science developed by the Polish Academy of Sciences. Signed consent for using serum was obtained from volunteers. The Rector’s Commission for Ethics of Scientific Research with Human Participation, University of Warsaw, Warsaw, Poland gave the ethical approval (approval number 51/2020). Human blood from four healthy persons was directly drawn into evacuated tubes and left undisturbed at room temperature for 1 h to clot. Tubes were centrifuged at 2500× *g* for 10 min to prevent possible platelet activation. Serum was pipetted out of the blood collection tubes, pooled and collected in microtubes.

### 3.7. In Vitro Enzymatic Stability Assay

A degradation assay was adapted from a method described previously [42,44,45,46]. Human blood serum was preactivated in Eppendorf ThermoMixer^®^ Comfort (Hamburg, Germany) at 37 °C for 20 min (350 rpm). Next, 20 μL of aqueous urea–peptide hybrid stock solution was added to give ∼750 μg/mL final compound concentration, and incubation was continued. At selected time intervals, 50 μL of the mixture was collected and quenched by adding 200 μL of ACN:H_2_O:FA mixture (89:10:1, *v*:*v*:*v*). The obtained suspension was vortexed for 1 min (3000 min^−1^) and centrifuged for 10 min in 4 °C (11,000× *g*). 100 μL of supernatant was collected and lyophilized. The samples reconstituted in 1 mL of 10 mM ammonium formate with 0.1% FA were subjected to LC-MS. Relative concentration at time intervals was calculated using peak area of either individual or most intense fragment of compound of interest. Analyst software version 1.7.1 was used for data acquisition, Peak View 2.2 and MultiQuant 2.1 (Sciex, ON) were used for data processing. Detailed analytical data can be found in Supplementary Materials (Figures S17–S45). GraphPad Prism Version-5.01 was used to present data and statistics. All experiments were independently conducted three times and results are represented as means ± SEMs. In order to show the significant difference in the concentration of intact substrate at each time interval depending on the sequence of the hybrid, a two-way ANOVA with Bonferroni’s post-tests was done (** *p* < 0.01, *** *p* < 0.001).

### 3.8. Cell Culture

The breast cancer cell line MDA-MB-231 was purchased from ATCC (France) and cultured in presence of DMEM complemented with 10% fetal calf serum (FCS), 1% penicillin–streptomycin and 1% glutamine (Invitrogen, France) at 37 °C, 5% CO_2_.

### 3.9. Flow Cytometry Analysis of Membrane and Intracytoplasmic NRP-1 Expression 

Cells were harvested using a mix of EDTA/Trypsin buffer (Invitrogen, France) to detach cells from plastic after 5 min exposure at 37 °C. Then cells were washed twice with complete medium to inactivate trypsin. Cell pellet was washed with 1X PBS with 2% FCS to perform FACS analysis. Cells were stained for 15 min at 4 °C with anti-neuropilin monoclonal antibodies BDCA-4: AD5-17F6 clone (Miltenyi Biotec^®^, France) or 12C2 clone (BioLegend^®^, USA), and with appropriate irrelevant IgG as control, both labeled with –PE or –APC, respectively. Intracytoplasmic staining was performed on 15 min PFA 4% fixed cells which were then permeabilized and stained using Saponin 2× buffer with antibodies described above. After washing with PBS containing 2% FCS, stained cells were analyzed using BD FACSCalibur^TM^ (San Jose, CA, USA) and FlowJo^TM^ cell analysis software (FlowJo LLC, Ashland, OR, USA).

### 3.10. Labelled Urea–Peptide Hybrid Binding Assays at 4 °C

Harvested cells were plated in 1× PBS, 2% FCS buffer in the absence or presence of 5/6-FAM-hybrid (**10**) at several concentrations (100, 50, 25 and 12.5 µg/mL) during 15 min at 4 °C. Unstained and FITC-irrelevant IgG cells were used as controls. Then, cells were washed with 1× PBS, 2% FCS buffer before BD FACSCalibur^TM^ acquisition and FlowJo^TM^ software analysis. This binding experiment is associated with a sequential NRP-1 staining (using clones describes above) or with appropriate irrelevant IgG as control (15 min at 4 °C) when it is necessary to prove the lack of antigen competition access for antibodies. 

### 3.11. Labelled Urea–Peptide Hybrid Binding Assays at 37 °C

Cells were stained with 5/6-FAM labeled urea–peptide hybrid (**10**) (100 µg/mL) at 4 °C, as described above. Next, washed cells were sequentially incubated at 37 °C during 0, 5, 15, 30 and 60 min in complete culture medium. After an ultimate wash with PBS supplemented with 2% FCS, cells were stained with APC-NRP-1 antibody or APC-irrelevant IgG as a control (15 min at 4°C). BD FACSCaliburTM acquisition and FlowJoTM software analysis were then performed. 

## 4. Conclusions

In this work, we presented a new group of urea–peptide hybrids based on our previous active structure Lys (*h*Arg)-Dab-Oic-Arg with improved inhibition of VEGF_165_/NRP-1 complex formation. The aim of our SAR study was to optimize the urea unit position in the branched portion of the parent molecule, which is important for ligand–protein interactions, and at the same time is a major enzyme cleavage site. The obtained results indicate that one of the synthesized compounds, namely, the hybrid **6** with the substitution of the Lys (*h*Arg) fragment by the containing urea bond fragment Dab(*h*Arg^U^), compared to the parent compound, showed not only an increase in the affinity for NRP-1 12-fold, but also a significant decrease in enzymatic degradation, by eliminating enzymatic cleavage of the branching. The latter feature is very important, as the prolongation of the plasma half-life is one of the crucial parameters of prospective drugs, as it helps to extend the time between drug applications. While the performed molecular dynamic and 2D NMR experiments did not give a direct answer for the increase in affinity due to unstable conformations of the analyzed compounds, the data suggest that urea moiety may favor additional ligand–protein interactions. Moreover, the presented in vitro experiments allowed us to prove that 5/6-FAM-labeled compound **6** binds to NRP-1 on the cell membrane and very quickly induces intracytoplasmic NRP-1 stock trafficking to the cell surface. In the near future, we plan to examine the signal paths that accompany this phenomenon. In summary, all the above-mentioned properties make hybrid **6** a good candidate for further development as an inhibitor of VEGF_165_/NRP-1 interaction.

## Figures and Tables

**Table 1:** The general structure and VEGF-A_165_/NRP-1 complex inhibitory effect of X(Z)-Dab-Oic-Arg branched urea–peptide hybrids. Urea bonds are marked in green.

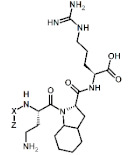 X(Z)-Dab-Oic-Arg			
Compound	X(Z) Branching	X(Z) Branching Structure	IC_50_ [µM] ^1^
**PP**	Lys(*h*Arg)	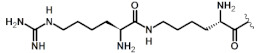	2.3 ^2^
**1**	Dab^U^(*h*Arg)	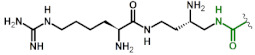	1.8 ^3^
**2**	Orn^U^(Arg)	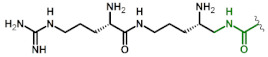	2.1 ^4^
**3**	Lys^U^(gDab)	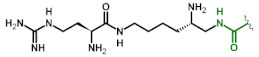	1.2 ^5^
**4**	Lys(gDab^U^)	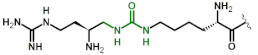	0.49 ^6^
**5**	Orn(Arg^U^)	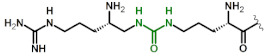	0.36 ^7^
**6**	Dab(*h*Arg^U^)	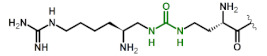	0.19 ^8^
**7**	Dap^U^(Arg^U^)	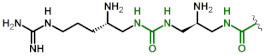	2.1 ^9^
**8**	Dab^U^(gDab^U^)	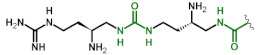	2.1 ^10^
**9**	Orn^U^(gDap^U^)	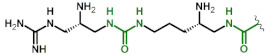	1.8 ^11^

^1^ R^2^ ranged between 0.992 and 0.999. ^2^ Data already published [44]. ^3^ logIC_50_ = −5.75 ± 0.03; ^4^ logIC_50_ = −5.67 ± 0.01; ^5^ logIC_50_ = −5.93 ± 0.03; ^6^ logIC_50_ = −6.306 ± 0.024; ^7^ logIC_50_ = −6.448 ± 0.031; ^8^ logIC_50_ = −6.726 ± 0.028; ^9^ logIC_50_ = −5.69 ± 0.02; ^10^ logIC_50_ = −5.69 ± 0.03; ^11^ logIC_50_ = −5.75 ± 0.03; compounds were tested in the concentration range 1–50 µM (compounds **1**–**3** and **7**–**9**) or 0.05–50 µM (compounds **4**–**6**). PP = parent peptide.

**Table 2:** Urea–peptide hybrid **6**’s inhibitory effect on VEGF-A_165_/receptor complex formation.

Compound	Sequence	IC_50_ [µM]			
		NRP-1	NRP-2	VEGF-R1	VEGF-R2
**6**	H-Dab(*h*Arg^U^)Dab-Oic-Arg-OH	0.19	0.48 ^1^	no affinity	no affinity

^1^ logIC50 = −6.323 ± 0.043; R2 = 0.984; compound was tested in the concentration range 0.1–10 µM.

## Supplementary Materials

Supplementary materials can be found at https://www.mdpi.com/1422-0067/22/1/72/s1.

## Abbreviations

anti-Tfr	anti-human transferrin receptor
5/6-FAM	5/6-carboxyfluorescein
Boc	tert-butoxycarbonyl
BSA	bovine serum albumin
bt	linear dichroism
Dab	2,4-diaminobutyric acid
Dap	2,3-diaminopropionic acid
DIPEA	*N,N*-diisopropylethylamine
ELISA	enzyme-linked immunosorbent assay
FACS	fluorescence-activated cell sorting
FCS	foetal calf serum
Fmoc	9-fluorenylmethoxycarbonyl
*h*Arg	homoarginine
HCTU	O-(1*H*-6-chlorobenzotriazole-1-yl)-1,1,3,3-tetramethyluronium hexafluorophosphate
NRP	neuropilin
Oic	octahydroindole-2-carboxylic acid
PBS	phosphate buffer saline
SAR	structure–activity relationship
strep-HRP	streptavidin–horseradish peroxidase conjugate
SWATH	sequential window acquisition of all theoretical mass spectra
TIS	triisopropylsilane
TFA	trifluoroacetic acid
VEGF	vascular endothelial grow factor
