# Supplementary material for: Urea-Peptide Hybrids as VEGF-A165/NRP-1 Complex Inhibitors with Improved Receptor Affinity and Biological Properties
Source: Int J Mol Sci. 2020 Dec 23;22(1):72. doi: 10.3390/ijms22010072 (PMC7793531; doi:10.3390/ijms22010072)
Supplement: Supplementary file 1 [file ijms-22-00072-s001.pdf]

SUPPLEMENTARY MATERIALS

Urea-peptide hybrids as VEGF-A_{165}/NRP-1 complex inhibitors with improved receptor affinity and biological properties

Anna K. Puszko[1,+,*], Piotr Sosnowski[2,+], Rachel Rignault-Bricard[3,4], Olivier Hermine[3,4], Gérard Hopfgartner[2], Karolina Pułka-Ziach[1], Yves Lepelletier[3,4] and Aleksandra Misicka[1,5,*]

[1] Faculty of Chemistry, University of Warsaw, Pasteura 1, 02-093 Warsaw, Poland; karola@chem.uw.edu.pl

[2] Department of Inorganic and Analytical Chemistry, University of Geneva, 24 Quai Ernest Ansermet, CH-1211 Geneva 4, Switzerland; piotr.sosnowski@unige.ch (P.S.); gerard.hopfgartner@unige.ch (G.H.)

[3] Université de Paris, Imagine Institute, 24 boulevard Montparnasse, 75015 Paris, France; rachel.rignault@parisdescartes.fr (R.R.B.); ohermine@gmail.com (O.H.); y.lepelletier@gmail.com (Y.L.)

[4] INSERM UMR 1163, Laboratory of cellular and molecular basis of normal hematopoiesis and hematological disorders: therapeutical implications, 24 boulevard Montparnasse, 75015 Paris, France;

[5] Department of Neuropeptides, Mossakowski Medical Research Centre, Polish Academy of Sciences, Pawinskiego 5, 02-106 Warsaw, Poland;

[*] Corresponding authors. Faculty of Chemistry, University of Warsaw, Pasteura 1, 02-093 Warsaw, Poland.
E-mail address: apuszko@chem.uw.edu.pl (A. K. Puszko), misicka@chem.uw.edu.pl (A. Misicka)
[+] These authors contributed equally to this work

Table of contents

1. General remarks **2**
2. General scheme of Boc-protected activated building block synthesis **2**
3. Building blocks synthesis and characterisation **3**
4. Analytical data of urea-peptide hybrids **16**
5. Dose-response curves of urea-peptide hybrids **31**
6. Inhibitory effect on VEGF-A_{165}/NRP-1 complex formation of hybrid **10** **31**
7. Analytical data of serum degradation **32**
8. 2D NMR characterisation of parent peptide H_2N-Lys(*h*Arg)-Dab-Oic-Arg-OH **63**
9. 2D NMR characterisation of hybrid **6** **66**
10. Molecular dynamics **69**
11. References **83**

1. General remarks. Fmoc-Arg(Pbf) Wang resin was purchased from Activotec (Cambridge, UK). Amino acids and coupling reagents were obtained from Iris Biotech (Marktredwitz, Germany). Solvents and reagents for building block synthesis were purchased from Merck (Darmstadt, Germany). Recombinant human receptors and biotinylated human VEGF-A_{165} were purchased from R&D Systems (Minneapolis, MN, USA). Chemiluminescent substrate was purchased from Thermo Scientific (Waltham, MA, USA). Microwave-assisted solid phase synthesis was carried out using CEM DiscoveryBio microwave (Matthews, NC, USA). Synthesized compounds were purified on Shimadzu Prominence semi-preparative HPLC system (Duisburg, Germany) equipped with a Phenomenex Jupiter Proteo C12 90Å 4 µm 250 x 10 mm column (Torrance, CA, USA). High resolution mass spectra (HRMS) and fragmentation spectra (MS/MS) were recorded on a SCIEX 6600TOF instrument with ESI ionization source and using infusion. The resolution power was of about 30'000 at *m/z* 300. The mass reported is containing the most abundant isotopes with mass error < 10 ppm. Luminescence was measured using a Tecan Infinite F200Pro microplate reader (Männedorf, Switzerland). ^{1}H NMR spectra were recorded on Bruker AVANCE 300 MHz. Chemical shifts are reported in parts per million (ppm). ^{1}H NMR splitting patterns with observed first-order coupling are designated as singlet (s), doublet (d), triplet (t) or multiplets (m). Broad peaks are designated as (b). 2D NMR spectra (COSY, TOCSY, ROESY, HSQC) were recorded on Agilent DD2 600 MHz spectrometer.

2. General scheme of Boc-protected activated building block synthesis.

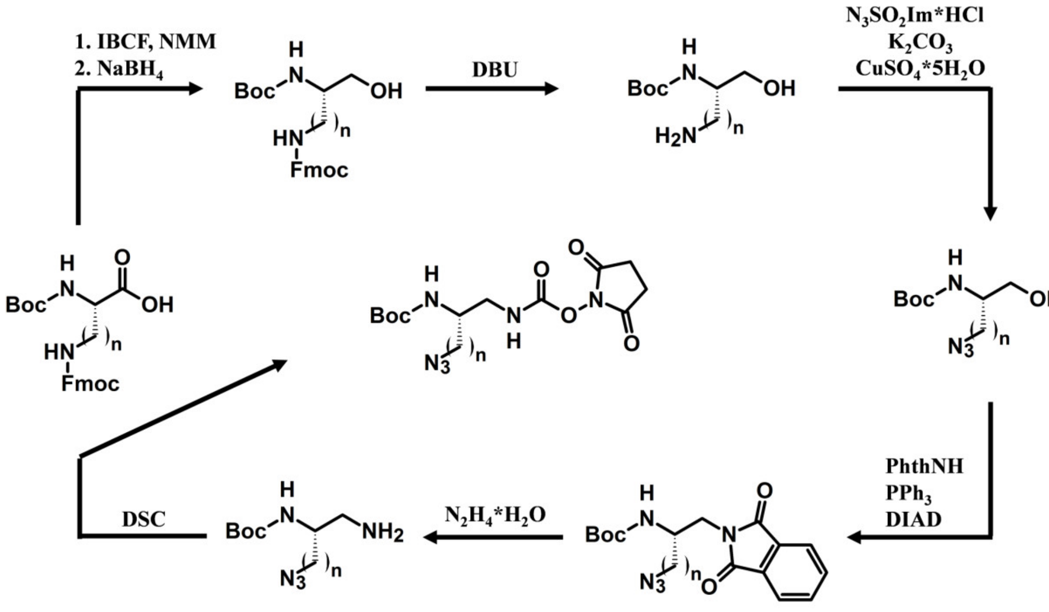

Scheme S1. General scheme of the synthesis of activated building blocks with Boc-protected α-amino group.

3. Building blocks synthesis and characterisation. Thin layer chromatography (TLC) was performed on silica gel 60 F254 (Merck) with detection by UV light and charring with ninhydrin in ethanol (1g in 200mL EtOH) followed by heating. Flash column chromatography was carried out on silica gel (63-200μm).

Building blocks were prepared according to the previously described procedures [1]. The starting substrates were Fmoc or Boc protected amino acids. Briefly, the first step in the synthesis was the conversion of an amino acid into an unsymmetrical anhydride and next reduction to the corresponding amino alcohol. Then the α-amino group (Fmoc-aminoalcohols) or side chain amino group (Boc-aminoalcohols) was deprotected and converted to the -N_3 group, according to the Wong method with diazotransfer reagent. The next step in the synthesis was to the conversion of the hydroxyl group to a protected amino group by the Mitsunobu reaction with phthalimide as a nitrogen source. After removal of the phthaloyl group with hydrazine hydrate, reaction with N,N'-disuccinimidyl carbonate (DSC) was performed to obtain activated carbamates.

2,5-dioxopyrrolidin-1-yl (*S*)-(2-azido-5-(3-((2,2,4,6,7-pentamethyl-2,3-dihydrobenzofuran-5-yl)sulfonyl)guanidino)pentyl)carbamate; N_3-Arg(Pbf) BB was characterized previously [2].

(*S*)-3-((((9H-fluoren-9-yl)methoxy)carbonyl)amino)-2-((tert-butoxycarbonyl)amino)propanoic acid; Boc-Dap(Fmoc)-ol: white solid; yield: 91% (flash chromatography: DCM/MeOH 95:5 v:v); ^{1}H NMR ($CDCl_3$, 300 MHz): δ 7.78-7.74 (m, 2H), 7.59-7.54 (m, 2H), 7.43-7.28 (m, 4H), 5.12 (bs, 1H), 5.03 (bs, 1H), 4.54-4.38 (m, 2H), 4.20 (t, *J* = 6.7 Hz, 1H), 3.60 (d, *J* = 9.8 Hz), 3.53-3.47 (t, overlapped with -CH_2 signal from Et_2O, 1H), 2.04 (bs, 2H), 1.43 (s, 9H).

***Tert*-butyl (*S*)-(1-azido-3-hydroxypropan-2-yl)carbamate; Boc-Dap(N_3)-ol:** oil, solidified to white solid; yield: 81% (flash chromatography: AcOEt:cycloheksane 9:1, 8:2, 7:3, 1:1 v:v); ^{1}H NMR ($CDCl_3$, 300 MHz): δ 4.97 (bs, 1H), 3.83-3.63 (m, 3H), 3.52 (m, 2H), 2.02 (s, 1H), 1.45 (s, 9H).

***Tert*-butyl (2,5-dioxopyrrolidin-1-yl) (3-azidopropane-1,2-diyl)(*R*)-dicarbamate; Boc-Dap(N_3) BB:** white solid; yield: 42%; total yield: 31%; ^{1}H NMR ($CDCl_3$, 300 MHz): δ 6.00 (t, *J* = 5.5 Hz, 1H), 4.95 (d, *J* = 8.1 Hz, 1H), 3.87 (d, *J* = 5.6 Hz, 1H), 3.51 (d, *J* = 4.6 Hz, 1H), 3.44-3.38 (m, 2H), 2.82 (s, 4H)1.71 (bs, 1H), 1.46 (s, 9H). HRMS calculated for $C_{13}H_{20}N_6O_6$ $[M+H]^+$: 357.1517 *m/z*, found:.357.1516 *m/z*, Δ: -0.3 ppm.

(9*H*-fluoren-9-yl)methyl *tert*-butyl (4-hydroxybutane-1,3-diyl)(*S*)-dicarbamate; Boc-Dab(Fmoc)-ol: white solid; yield: 92% (flash chromatography: DCM/MeOH 95:5 v:v); ^{1}H NMR ($CDCl_3$, 300 MHz): δ 7.78-7.73 (m, 2H), 7.63-7.57 (m, 2H), 7.43-7.27 (m, 4H), 5.52 (bs, 1H), 4.85 (bs, 1H), 4.39 (d, *J* = 6.0 Hz, 2H), 4.22 (t, *J* = 7.0 Hz, 1H), 3.71 (d, *J* = 8.2 Hz, 2H), 3.07 (s, 1H), 1.97 (bs, 2H), 1.75-1.55 (m, 2H), 1.46 (s, 9H).

***Tert*-butyl (*S*)-(4-azido-1-hydroxybutan-2-yl)carbamate; Boc-Dab(N_3)-ol:** oil, solidified to white solid; yield: 87% (flash chromatography: AcOEt:cycloheksane 9:1, 8:2, 7:3, 1:1 v:v); ^{1}H NMR ($CDCl_3$,

300 MHz): δ 4.80 (bs, 1H), 3.78-3.57 (m, 3H), 3.42 (t, J = 6.1 Hz, 2H), 2.07 (bs, 1H), 1.89-1.65 (m, 2H), 1.45 (s, 9H).

***Tert*-butyl (2,5-dioxopyrrolidin-1-yl) (4-azidobutane-1,2-diyl)(*S*)-dicarbamate; Boc-Dab(N_3) BB:** white solid; yield: 60%; total yield: 54%; ^{1}H NMR ($CDCl_3$, 300 MHz): δ 6.06 (t, J = 5.4 Hz, 1H), 4.77 (bs, 1H), 3.81 (bs, 1H), 3.49-3.3.28 (m, 4H), 2.82 (s, 4H), 1.85-1.62 (m, 2H), 1.45 (s, 9H). HRMS calculated for $C_{14}H_{22}N_6O_6$ [M+H]$^+$: 371.1673 *m/z*, found: 371.1705 *m/z*, Δ: 7.6 ppm.

(9*H*-fluoren-9-yl)methyl *tert*-butyl (5-hydroxypentane-1,4-diyl)(*S*)-dicarbamate; Boc-Orn(Fmoc)-ol: white solid; yield: 85% (flash chromatography: DCM/MeOH 95:5 v:v); ^{1}H NMR ($CDCl_3$, 300 MHz): δ 7.78-7.73 (m, 2H), 7.61-7.58 (m, 2H), 7.44-7.27 (m, 4H), 4.92 (bs, 1H), 4.66 (bs, 1H), 4.41 (d, J = 6.6 Hz, 2H), 4.21 (t, J = 6.8 Hz, 1H), 3.66 (d, J = 9.3 Hz, 2H, overlapped with -CH_2 signal from Et_2O), 3.22 (s, 1H), 1.87 (bs, 2H), 1.67-1.48 (m, 4H), 1.45 (s, 9H).

***Tert*-butyl (*S*)-(5-azido-1-hydroxypentan-2-yl)carbamate; Boc-Orn(N_3)-ol:** oil, solidified to white solid; yield: 82% (flash chromatography: AcOEt:cycloheksane 9:1, 8:2, 7:3 v:v); ^{1}H NMR ($CDCl_3$, 300 MHz): δ 4.64 (bs, 1H), 3.72-3.52 (m, 3H), 3.35-3.28 (t, J = 6.2 Hz, 2H), 1.91 (s, 1H), 1.73-1.49 (m, 4H), 1.45 (s, 9H).

***Tert*-butyl (2,5-dioxopyrrolidin-1-yl) (5-azidopentane-1,2-diyl)(*S*)-dicarbamate Boc-Orn(N_3) BB:** white solid; yield: 42%; total yield: 29%; ^{1}H NMR ($CDCl_3$, 300 MHz): δ 6.01 (s, 1H), 4.59 (d, J = 8.2 Hz, 1H), 3.72 (s, 1H), 3.43-3.21 (m, 4H), 2.82 (s, 4H), 1.74-1.54 (m, 4H), 1.45 (s, 9H). HRMS calculated for $C_{15}H_{24}N_6O_6$ [M+H]$^+$: 385.1830 *m/z*, found:.385.1850 *m/z*, Δ: 7.6 ppm.

Benzyl *tert*-butyl (6-hydroxyhexane-1,5-diyl)(*S*)-dicarbamate; Boc-Lys(Z)-ol was characterized previously [3].

***Tert*-butyl (*S*)-(6-azido-1-hydroxyhexan-2-yl)carbamate; Boc-Lys(N_3)-ol:** oil, solidified to white solid; yield: 98% (flash chromatography: AcOEt:cycloheksane 9:1, 8:2, 1:1 v:v); ^{1}H NMR ($CDCl_3$, 300 MHz): δ 4.66 (bs, 1H), 3.71-3.50 (m, 3H), 3.28 (t, J = 6.7 Hz, 2H), 2.30 (bs, 1H), 1.73-1.45 (m, 6H), 1.45 (s, 9H).

***Tert*-butyl (2,5-dioxopyrrolidin-1-yl) (6-azidohexane-1,2-diyl)(*S*)-dicarbamate; Boc-Lys(N_3) BB:** white solid; yield: 50%; total yield: 46%; ^{1}H NMR ($CDCl_3$, 300 MHz): δ 6.03 (s, 1H), 4.55 (d, J = 7.7 Hz, 1H), 3.69 (bs, 1H), 3.42-3.32 (m, 1H), 3.28 (t, J = 6.6 Hz, 3H), 2.82 (s, 4H), 1.77-1.47 (m, 6H), 1.45 (s, 9H). HRMS calculated for $C_{16}H_{26}N_6O_6$ [M+H]$^+$: 399.1986 *m/z*, found: 399.1976 *m/z*, Δ: -2.5 ppm.

(9*H*-fluoren-9-yl)methyl (*S*)-(1-hydroxy-6-(3-((2,2,4,6,7-pentamethyl-2,3-dihydrobenzofuran-5-yl)sulfonyl)guanidino)hexan-2-yl)carbamate; Fmoc-*h*Arg(Pbf)-ol: white solid; yield: 85% (flash chromatography: DCM/MeOH 95:5 v:v); ^{1}H NMR ($CDCl_3$, 300 MHz): δ 7.65 (d, J = 7.4 Hz, 2H), 7.49 (d, J = 7.4 Hz, 2H), 7.29 (t, J = 7.4 Hz, 2H), 7.22-7.18 (m, 2H, signal overlapped with $CHCl_3$), 6.46 (s, 1H), 5.40 (d, J = 7.2 Hz, 1H), 4.27 (d, J = 7.0 Hz, 2H), 4.11-4.04 (m, 1H), 3.57-3.47 (m, 2H), 3.40 (t, J = 7.0 Hz, 2H), 3.11 (bs, 2H), 2.84 (s, 2H), 2.48 (s, 3H), 2.41 (s, 3H), 1.99 (s, 3H), 1.57-1.37 (m, 6H), 1.36 (s, 6H).

(*S*)-N-(N-(5-azido-6-hydroxyhexyl)carbamimidoyl)-2,2,4,6,7-pentamethyl-2,3-dihydrobenzofuran-5-sulfonamide; N_3-*h*Arg(Pbf)-ol: white foam; yield: 73% (flash chromatography: AcOEt:cycloheksane 9:1, 8:2, 7:3 v:v); ^{1}H NMR ($CDCl_3$, 300 MHz): δ 6.40 (s+bs, 3H), 3.69-3.52 (m, 2H), 3.43-3.35 (m, 1H), 2.23-2.14 (m, 2H), 2.95 (s, 2H), 2.55 (s, 3H), 2.48 (s, 3H), 2.09 (s, 3H), 1.63-1.1.47 (m, 6H), 1.46 (s, 6H).

2,5-dioxopyrrolidin-1-yl (*S*)-(2-azido-6-(3-((2,2,4,6,7-pentamethyl-2,3-dihydrobenzofuran-5-yl)sulfonyl)guanidino)hexyl)carbamate; N_3-*h*Arg (Pbf) BB: white solid; yield: 35%; total yield: 18%; ^{1}H NMR ($CDCl_3$, 300 MHz): δ 6.48 (bs, 1H), 3.62-3.57 (m, 1H), 3.46-3.39 (m, 1H), 3.35-3.20 (m, 3H), 2.98 (s, 2H), 2.85 (s, 4H), 2.58 (s, 3H), 2.52 (s, 3H), 2.12 (s, 3H), 1.74-1.56 (m, 6H), 1.27 (s, 6H). HRMS calculated for $C_{25}H_{36}N_8O_7S$ $[M+H]^+$: 593.2500 *m/z*, found:593.2473 *m/z*, Δ: -4.6 ppm.

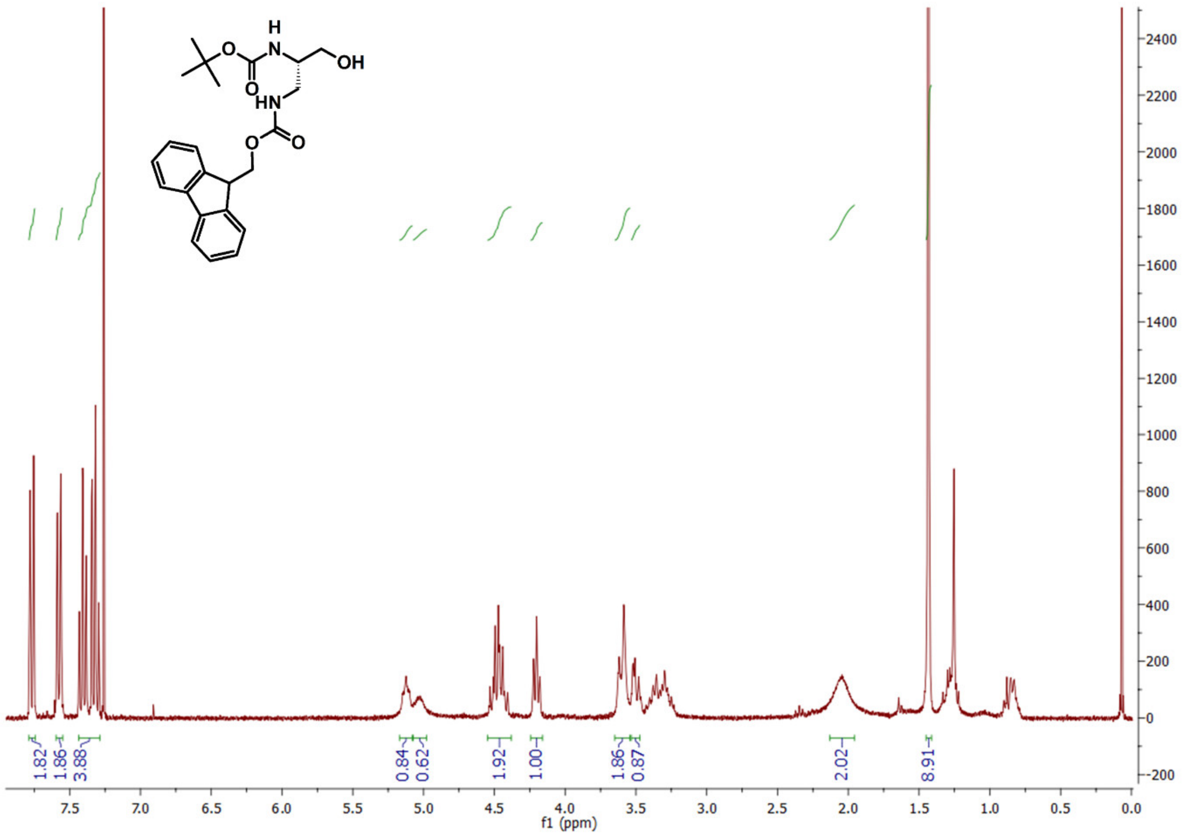

H
N
O
O
OH
HN
O
O
1.82
1.86
3.88
0.84
0.62
1.92
1.00
1.86
0.87
2.02
8.91
f1 (ppm)

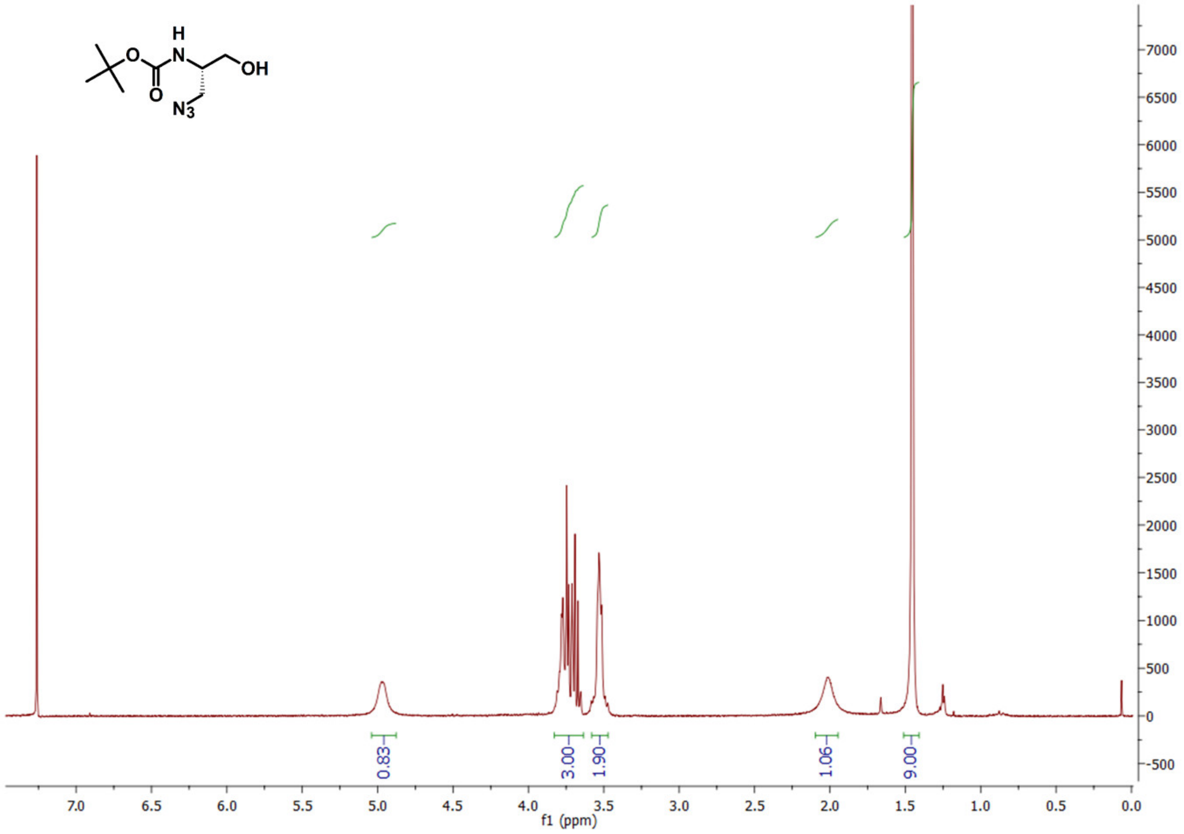

H
N
O
O
OH
N3
0.83
3.00
1.90
1.06
9.00
f1 (ppm)

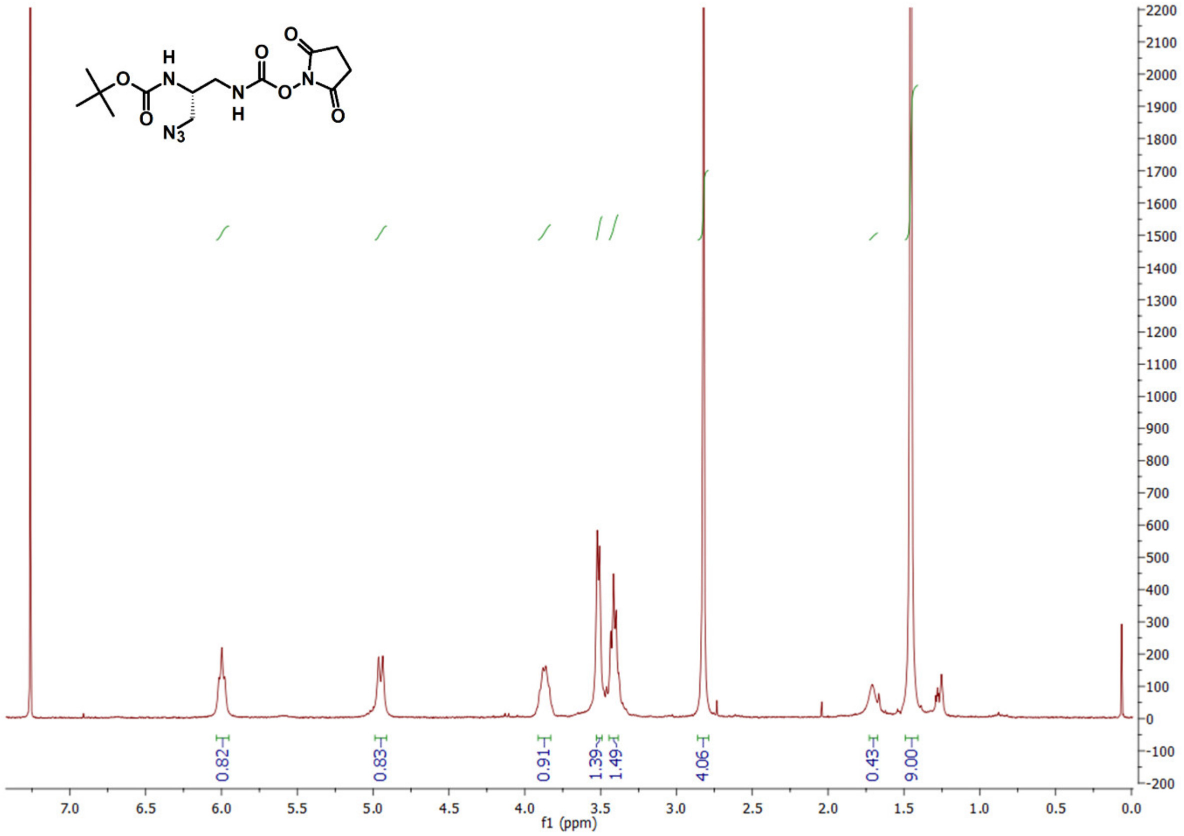

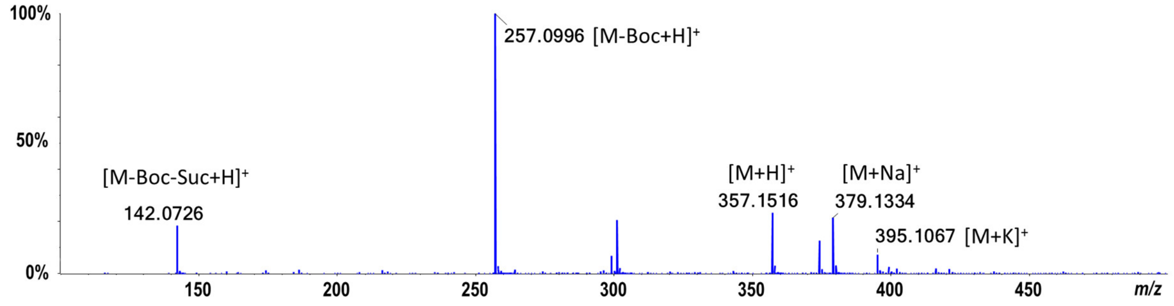

Figure S1. ^{1}H NMR (300MHz, $CDCl_3$) of Boc-Dap(Fmoc)-ol, Boc-Dap(N_3)-ol and Boc-Dap(N_3) BB and HRMS spectrum of Boc-Dap(N_3) BB.

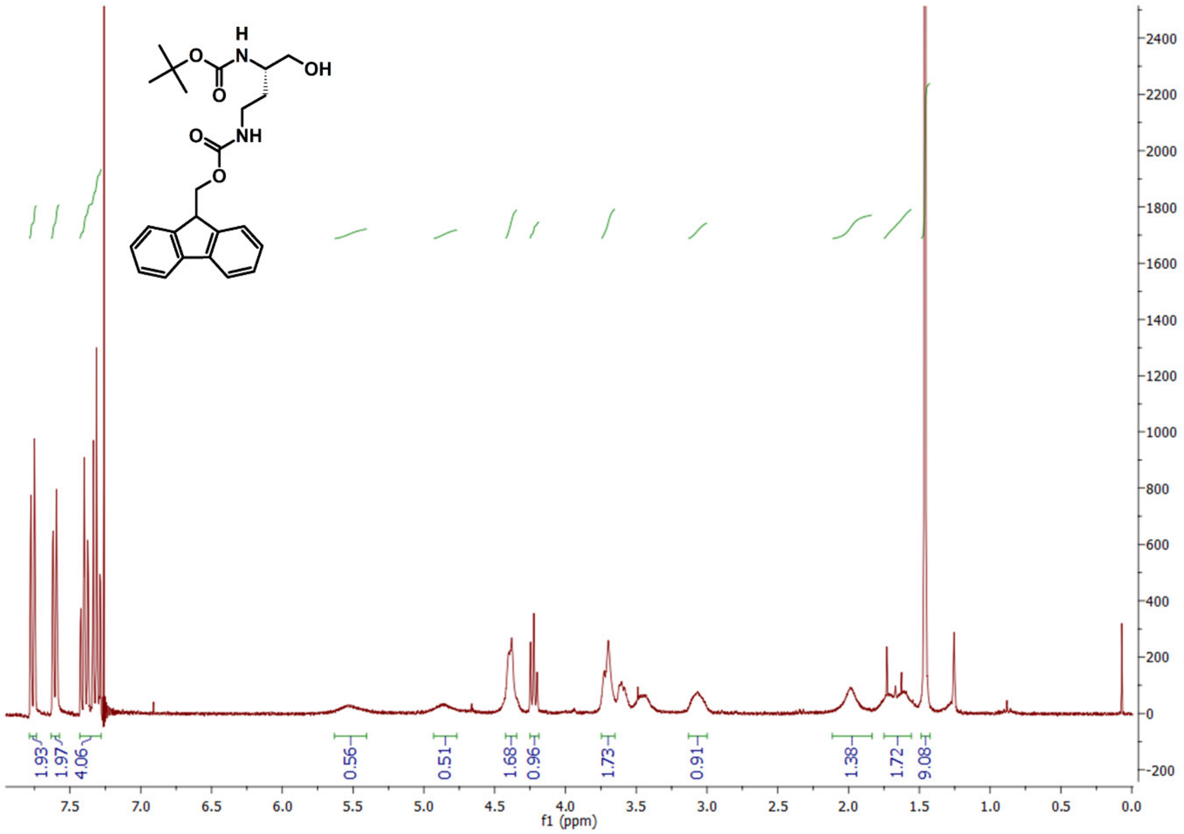
1.93
1.97
4.06
0.56
0.51
1.68
0.96
1.73
0.91
1.38
1.72
9.08
f1 (ppm)

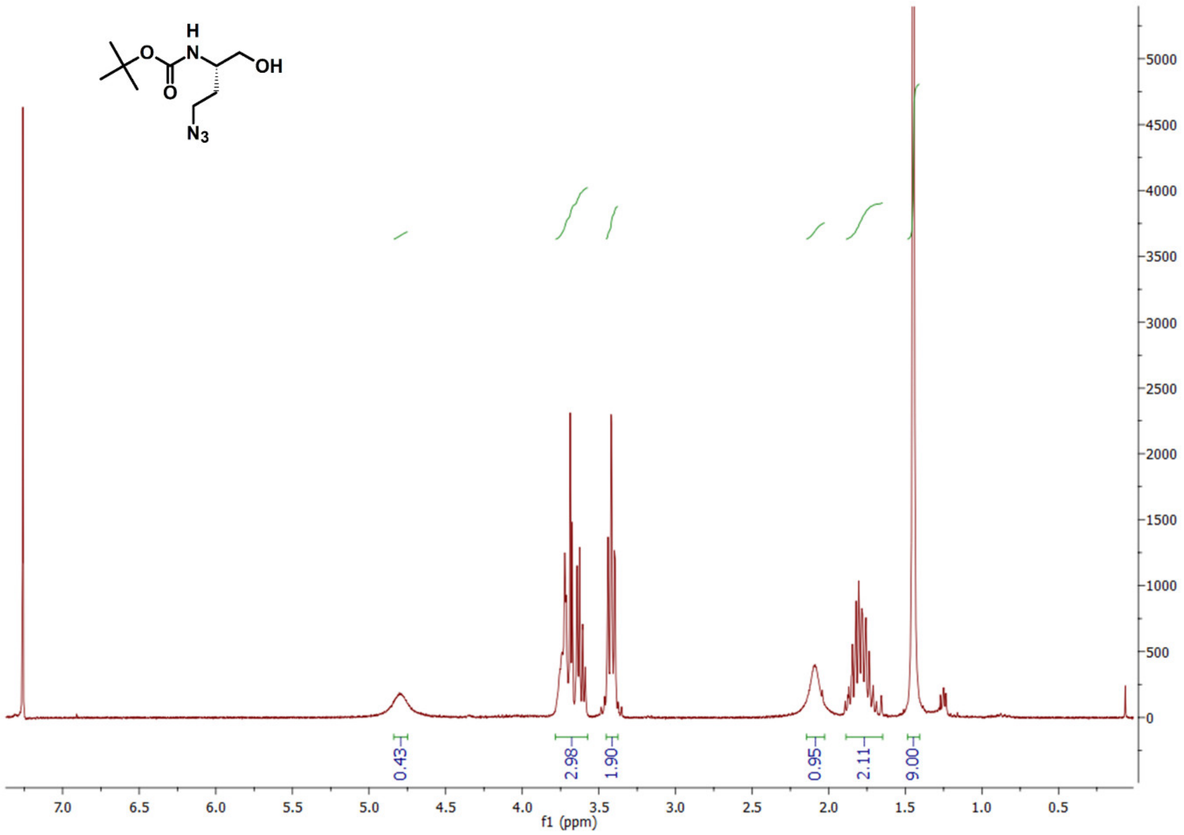
0.43
2.98
1.90
0.95
2.11
9.00
f1 (ppm)

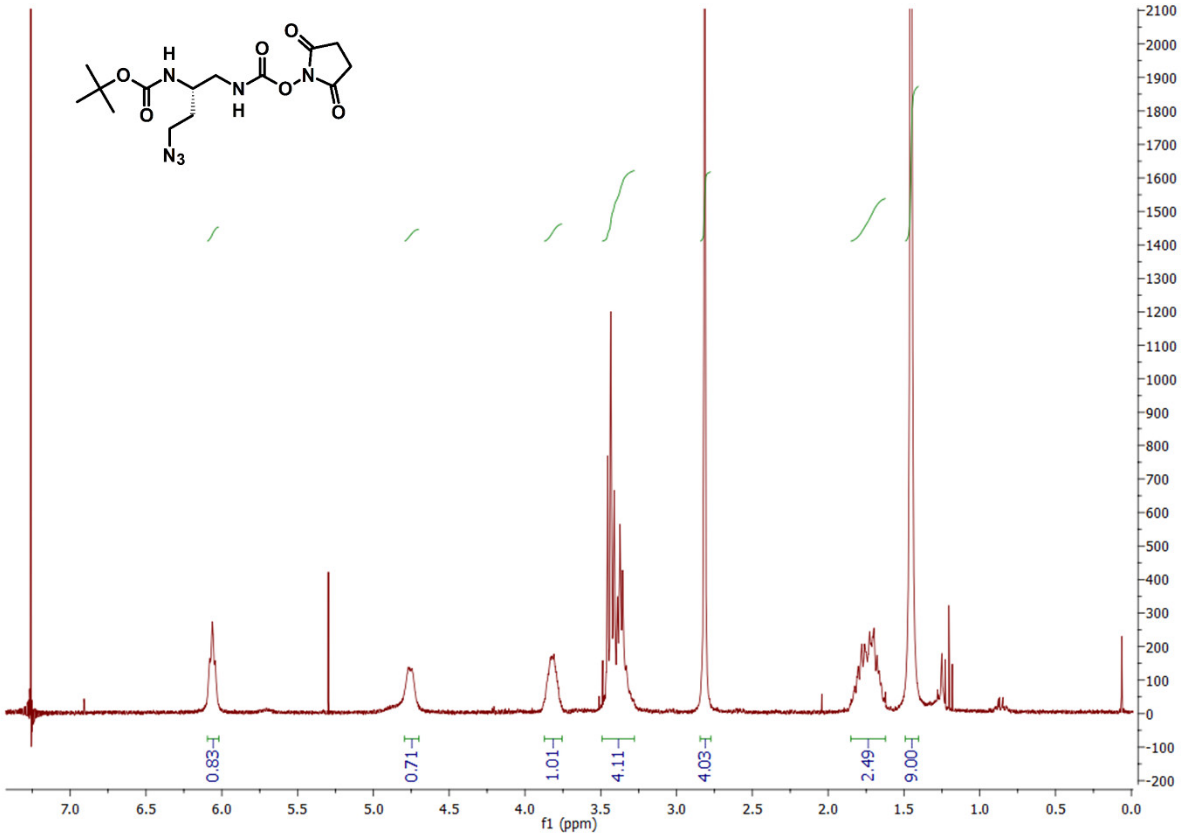

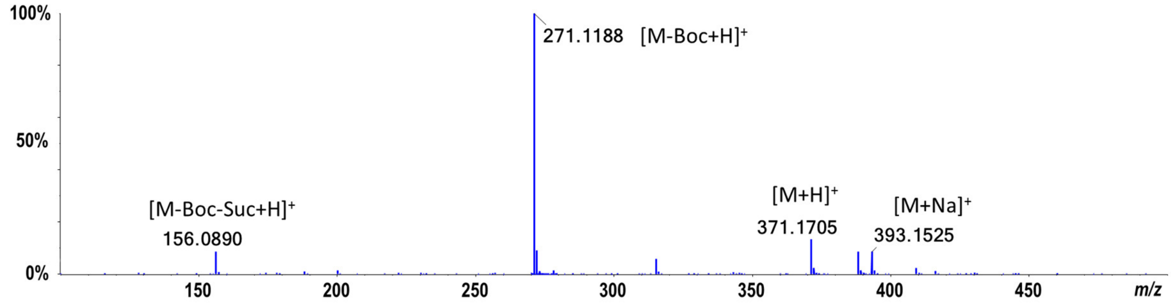

Figure S2. ^{1}H NMR (300MHz, $CDCl_3$) of Boc-Dab(Fmoc)-ol, Boc-Dab(N_3)-ol and Boc-Dab(N_3) BB and HRMS spectrum of Boc-Dab(N_3) BB.

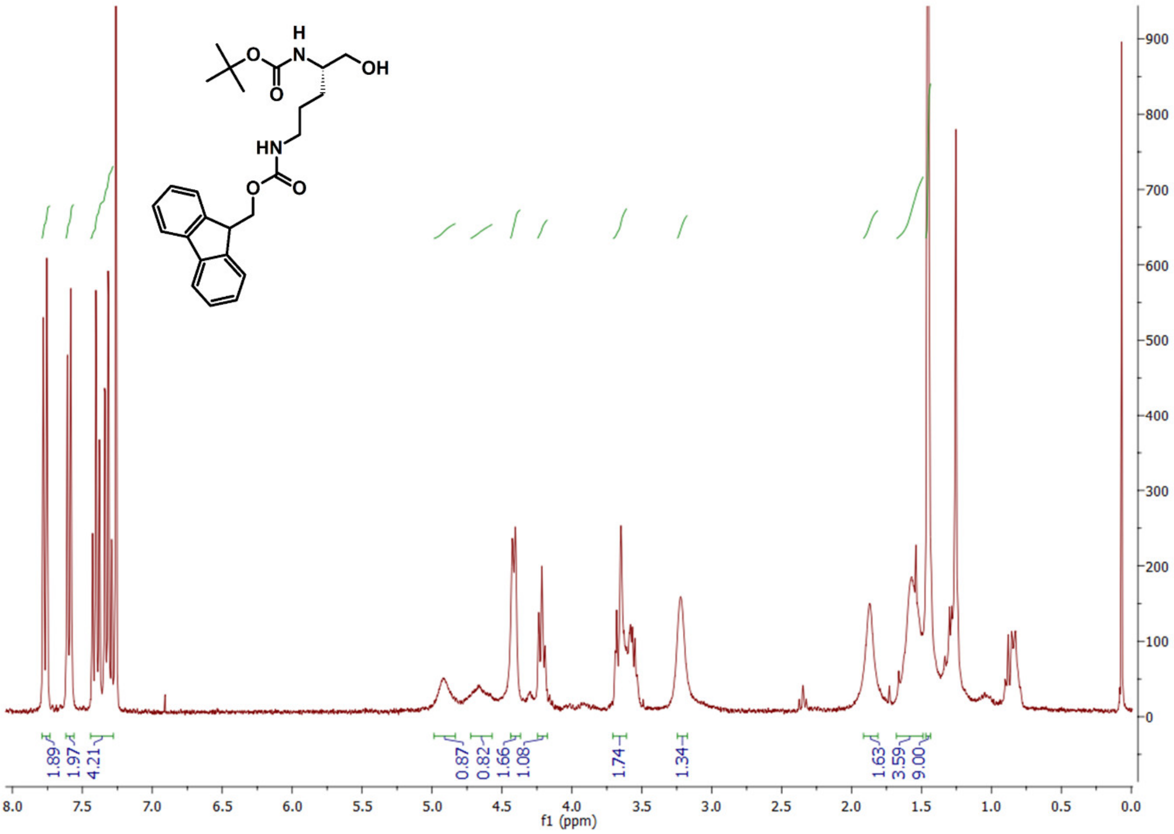

H
N
O
OH
HN
O
O
O
1.89
1.97
4.21
0.87
0.82
1.66
1.08
1.74
1.34
1.63
3.59
9.00
f1 (ppm)

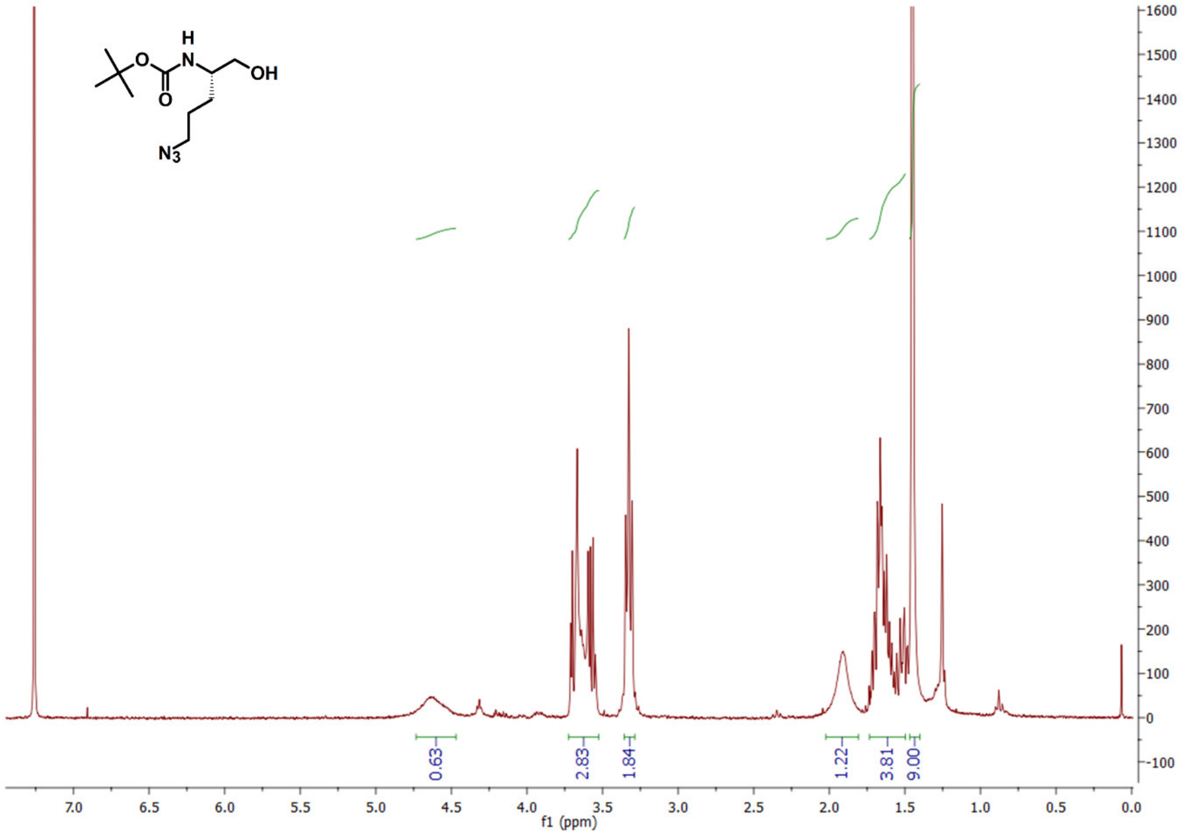

H
N
O
OH
O
N_3
0.63
2.83
1.84
1.22
3.81
9.00
f1 (ppm)

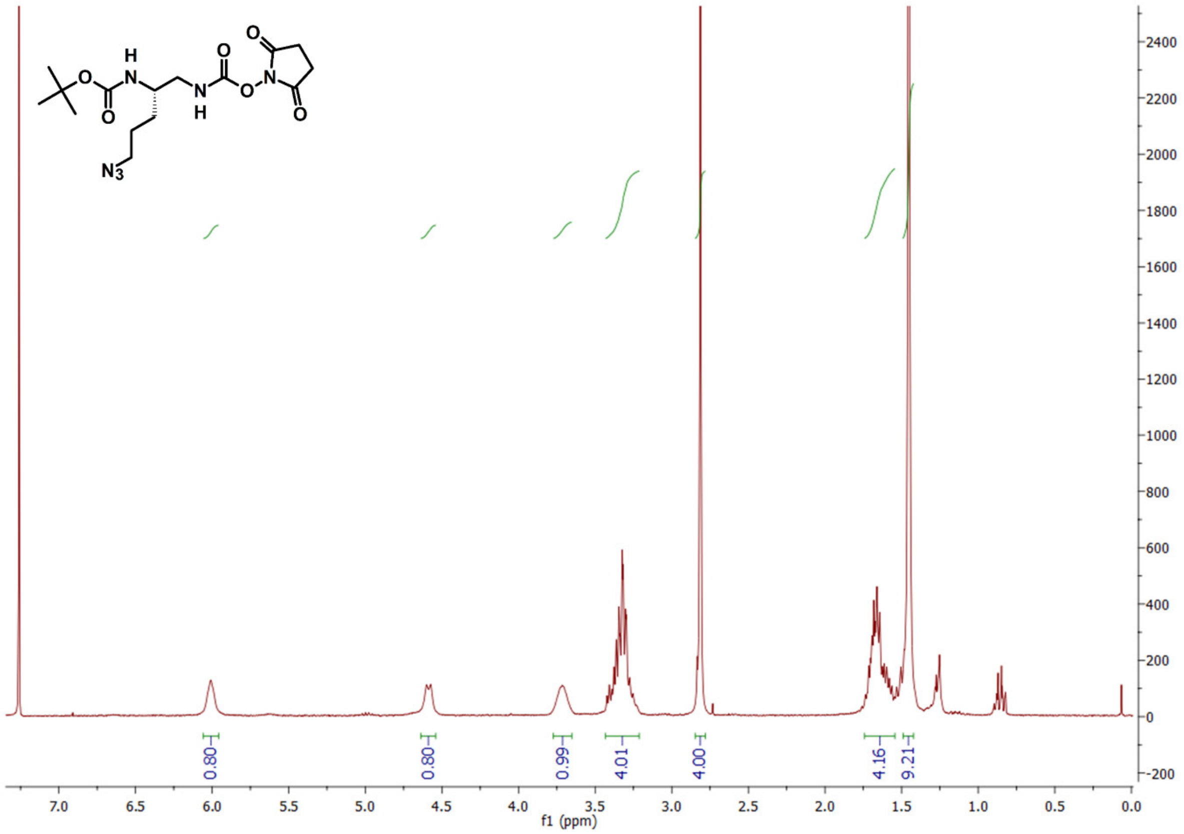

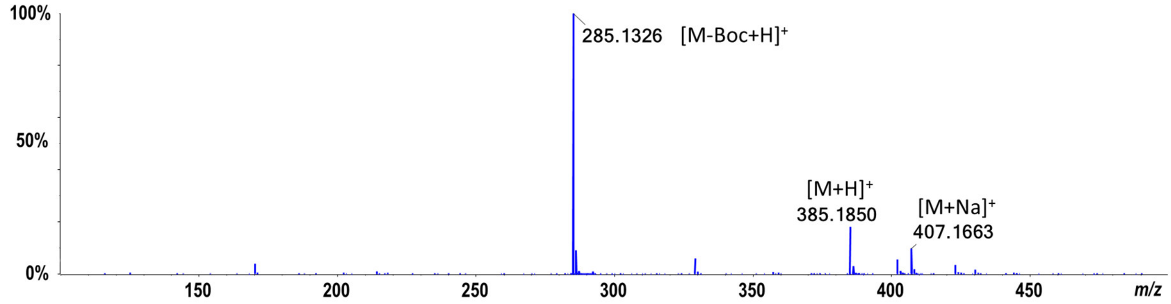

Figure S3. ^{1}H NMR (300MHz, $CDCl_3$) of Boc-Orn(Fmoc)-ol, Boc-Orn(N_3)-ol and Boc-Orn(N_3) BB and HRMS spectrum of Boc-Orn(N_3) BB**.**

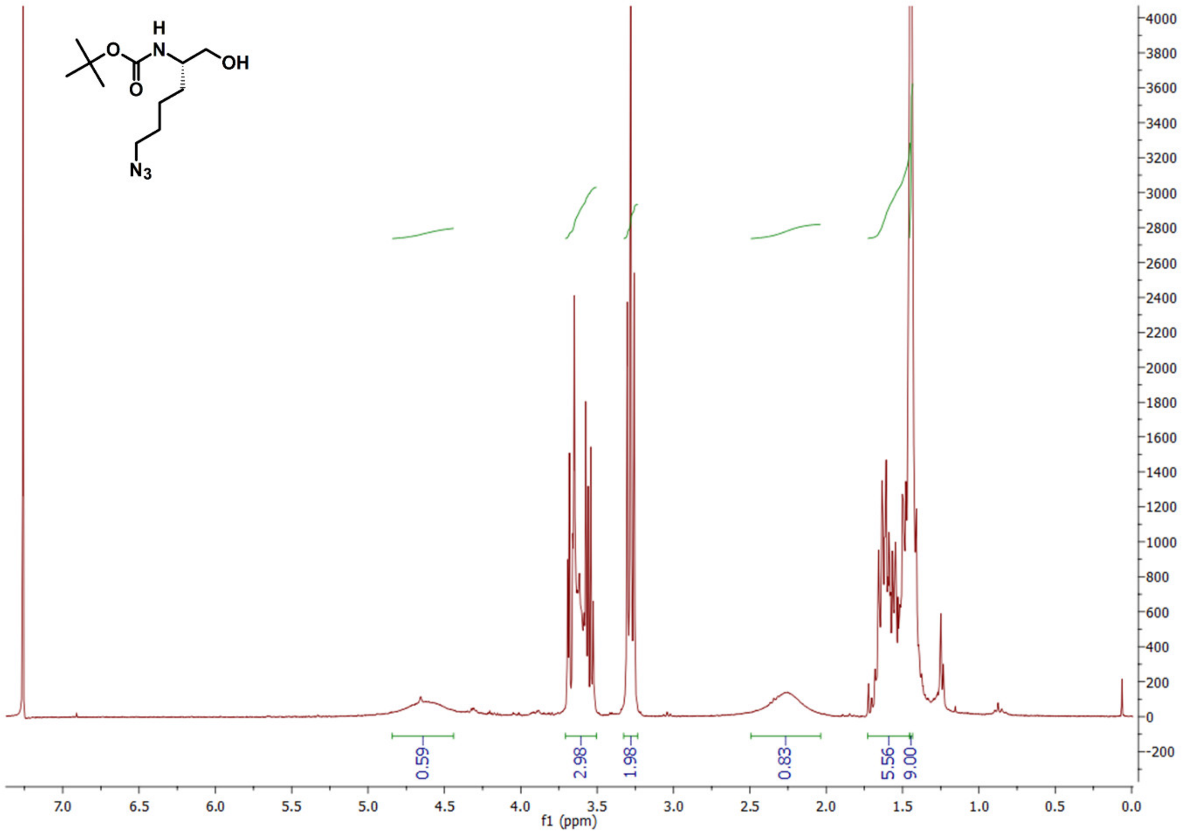

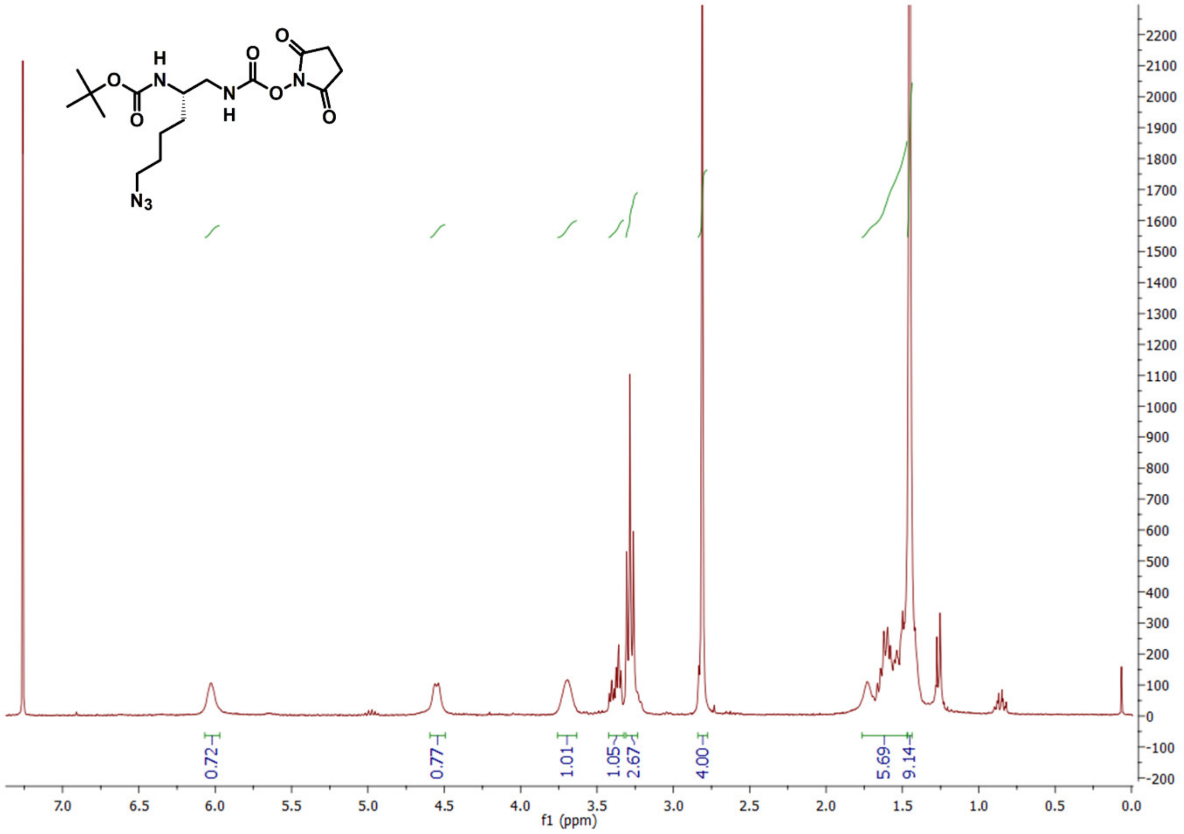

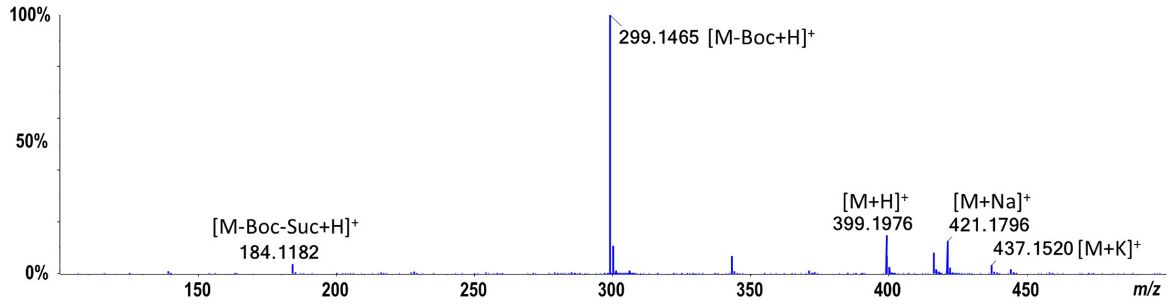

Figure S4. ^{1}H NMR (300MHz, $CDCl_3$) of Boc-Lys(N_3)-ol and Boc-Lys(N_3) BB and HRMS spectrum of Boc-Lys(N_3) BB.

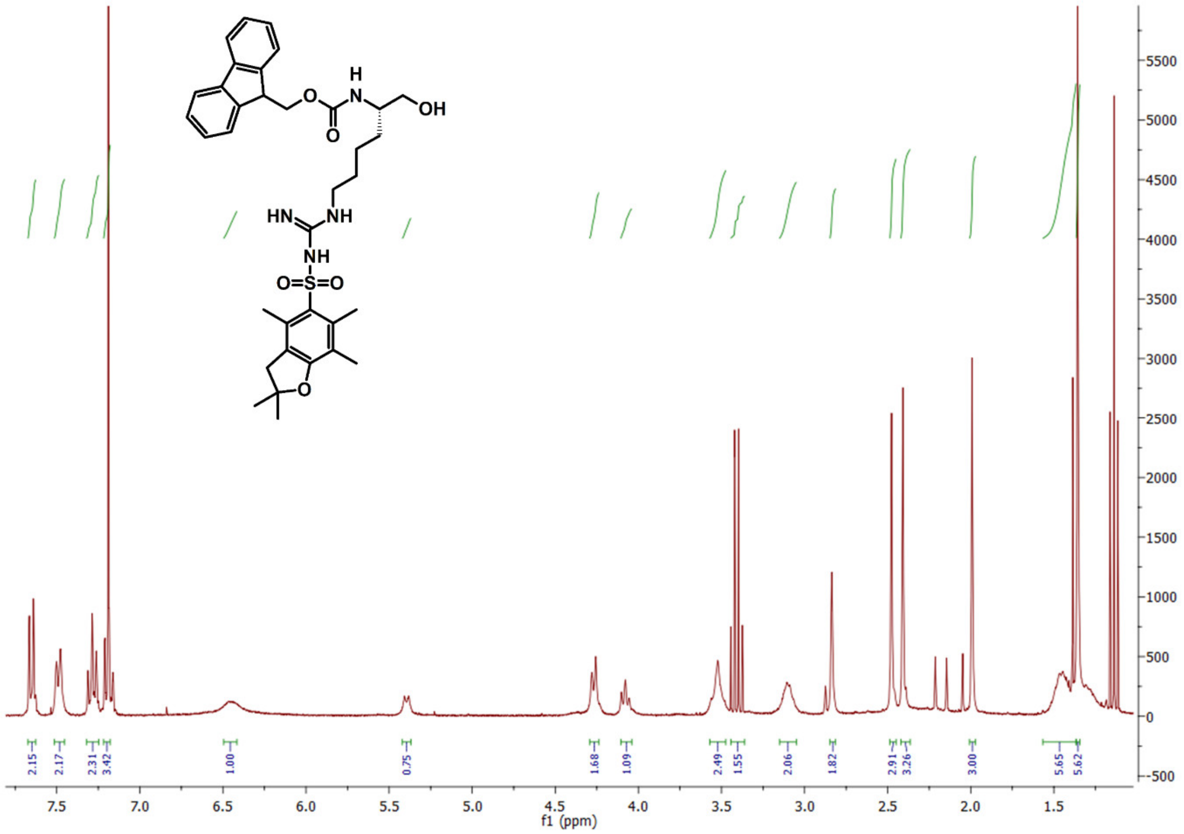

H
N
O
O
OH
HN
NH
NH
O=S=O
O
f1 (ppm)

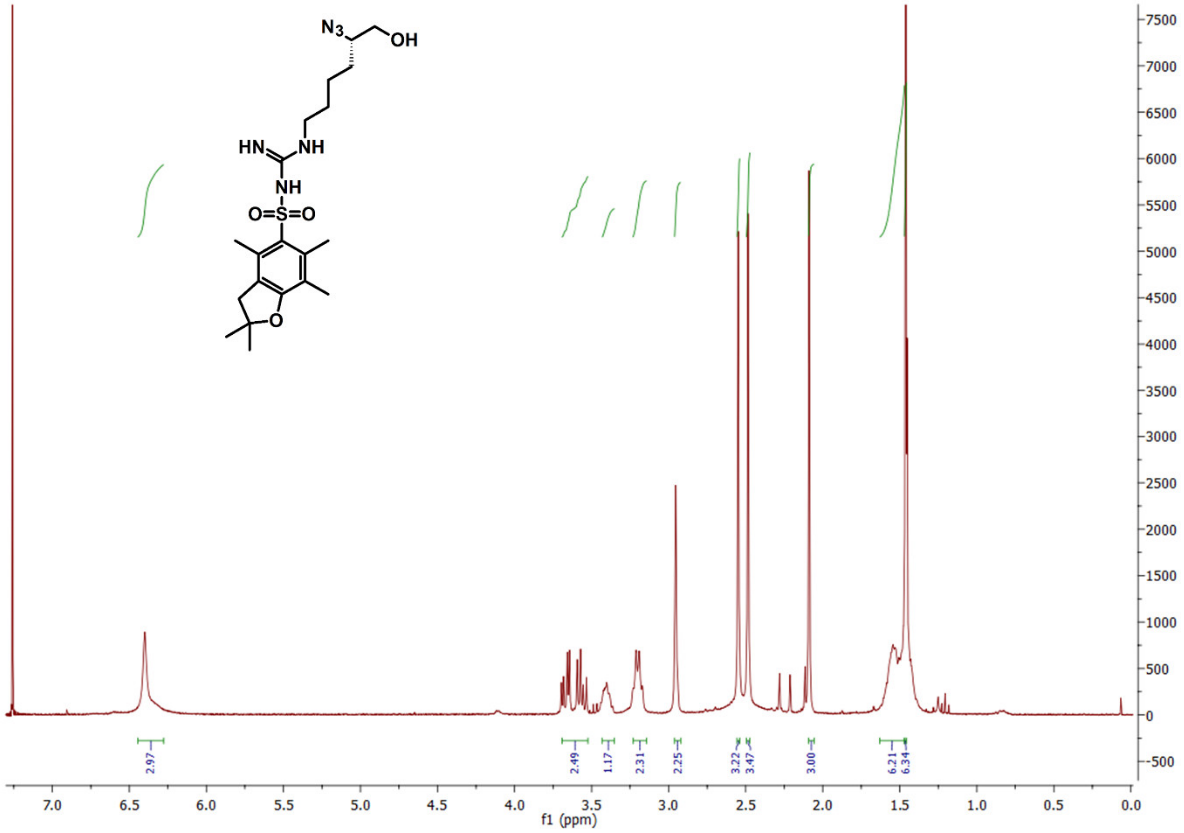

N_3
OH
HN
NH
NH
O=S=O
O
f1 (ppm)

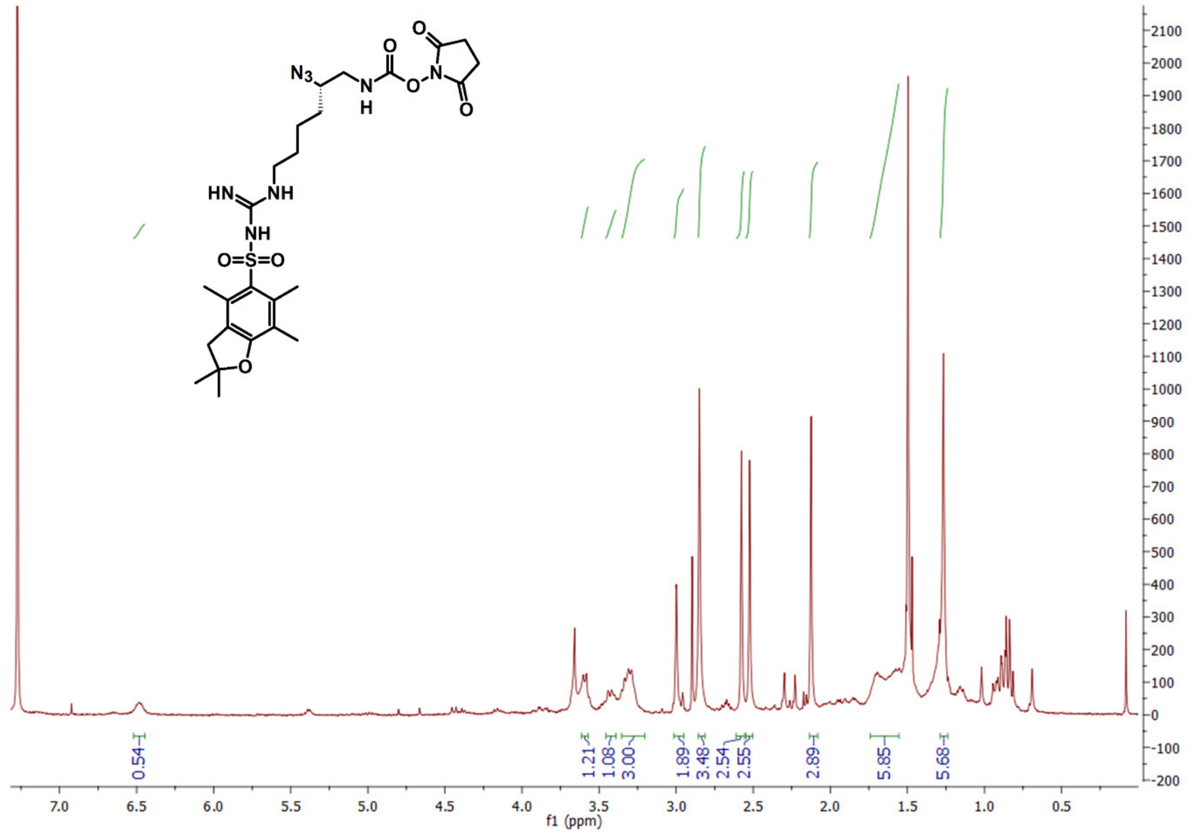

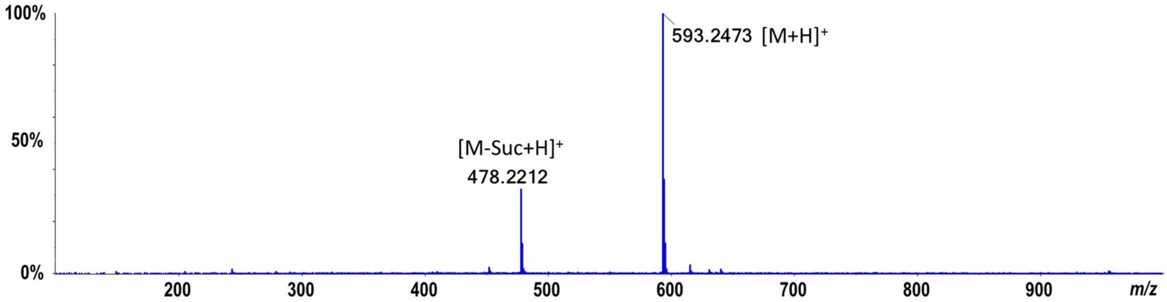

Figure S5. ^{1}H NMR (300MHz, $CDCl_3$) of Fmoc-*h*Arg(Pbf)-ol, N_3-*h*Arg(Pbf)-ol and N_3-*h*Arg(Pbf) BB and HRMS spectrum of N_3-*h*Arg(Pbf) BB.

4. Analytical data of urea-peptide hybrids. Purity of compounds (> 98%) was determined using RP-HPLC with UV detection. Analysis of pure products was carried out by HPLC with a Shimazu Prominence HPLC system (Duisburg, Germany) with binary pump system LC-20AD and autosampler SIL-20AC HT coupled to a SPD-20A UV detector. Chromatographic separation was achieved on Phenomenex Jupiter Proteo C12 column (250 × 4.6 mm) (Torrance, CA, USA) at 35˚C. Mobile phases consisted of H_2O:TFA (99.9:0.1 v/v, phase A) and ACN:TFA (99.9:0.1 v/v, phase B) at a flow rate of 1 mL/min. Elution was performed with gradient as follows: 0 – 20% B in 20 min.

1 mg/ml solution of each compound was prepared in H_2O and 10 μL was injected. UV detection was performed at λ = 200 nm. Purity of compounds was estimated using peak area.

Background substracted high resolution resolution mass spectra (HRMS) and high resolution fragmentation spectra (MS/MS) were recorded on a SCIEX TripleTOF 6600 instrument with ESI ionization source and by infusion at 10 μL/min. 0.1 mg/ml solutions of each compound were prepared in 50% MeOH 0.1% FA.

The electrospray ionization (ESI) was operated in positive mode. Curtain gas (CUR) was set to 25 psi. Nebulizing gas (GS1) was set to 20 psi and drying gas (GS2) was set to 15 psi. Needle voltage (ISVF) was set to 5kV and temperature (TEM) was set to 50ºC. Declustering potential (DP) was set to 80 V. To induce fragmentation, collision energy voltage (CE) was set to 30 V and collision energy spread voltage (CES) was set to 15 V. Mass spectrometer was operated in TOFMS mode in range the 100-2000 *m*/*z* and in Product Ion (MS/MS) mode in range the 100-1000 *m/z.*

Theoretical $[M+nH]^{n+}$ values and errors were calculated using Mass Calculators tool integrated with spectrometer operating software.

Table S1. Molecular weight, reaction yields and HPLC analytical data of compounds **1-10**.

Compound	MW [g/mol]	MW_{+TFA} [g/mol]	Yield	RT [min]
**1**	724.9	1294.9	59%	18.66
**2**	724.9	1294.9	63%	18.46
**3**	724.9	1294.9	51%	18.84
**4**	724.9	1294.9	65%	18.51
**5**	724.9	1294.9	55%	18.31
**6**	724.9	1294.9	59%	18.98
**7**	725.9	1295.9	55%	18.51
**8**	725.9	1295.9	63%	18.40
**9**	725.9	1295.9	51%	18.51
**10**	1083.2	1539.2	39%	19.90

Table S2. HRMS analytical data of compounds **1-10**.

Compound	Molecular formula	$[M+H]^+$ calculated	$[M+H]^+$ found	Error [ppm]	$[M+2H]^{2+}$ calculated	$[M+2H]^{2+}$ found	Error [ppm]	$[M+3H]^{3+}$ calculated	$[M+3H]^{3+}$ found	Error [ppm]
**1**	$C_{31}H_{60}N_{14}O_6$	725.4893	725.4894	0.2	363.2483	363.2500	4.7	242.5013	-	-
**2**	$C_{31}H_{60}N_{14}O_6$	725.4893	725.48692	-3.5	363.2483	363.24852	0.4	242.5013	-	-
**3**	$C_{31}H_{60}N_{14}O_6$	725.4893	725.4871	-3.0	363.2483	363.24840	1.7	242.5013	-	-
**4**	$C_{31}H_{60}N_{14}O_6$	725.4893	725.4886	-0.9	363.2483	363.2483	0.0	242.5013	242.5014	0.4
**5**	$C_{31}H_{60}N_{14}O_6$	725.4893	725.4883	-1.4	363.2483	363.2482	-0.3	242.5013	242.5013	0.0
**6**	$C_{31}H_{60}N_{14}O_6$	725.4893	725.4871	-3.1	363.2483	363.2479	-1.0	242.5013	242.5012	-0.2
**7**	$C_{30}H_{59}N_{15}O_6$	726.4846	726.4811	-4.8	363.7459	363.7459	0.0	242.8330	242.8328	-0.8
**8**	$C_{30}H_{59}N_{15}O_6$	726.4846	726.4811	-4.8	363.7459	363.7459	0.0	242.8330	242.8326	-1.6
**9**	$C_{30}H_{59}N_{15}O_6$	726.4846	726.4818	-3.9	363.7459	363.7461	0.5	242.8330	242.8325	-2.1
**10**	$C_{52}H_{70}N_{14}O_{12}$	1083.5370	1083.5359	-1.0	542.2722	542.2725	0.6	361.8505	361.8516	3.0

Table S3. MS/MS analytical data of compounds **1-10**.

**1**	**fragment formula**	***m/z* calculated**	***m/z* found**	**Error [ppm]**
	$C_6H_{15}N_4O_2^+$	175.1190	175.1197	4.0
	$C_{11}H_{28}N_7O^+$	274.2350	274.2370	7.3
	$C_{12}H_{26}N_7O_2^+$	300.2143	300.2162	6.3
	$C_{15}H_{28}N_5O_3^+$	326.2187	326.2206	5.8
	$C_{16}H_{34}N_9O_3^+$	400.2779	400.2794	3.7
	$C_{19}H_{36}N_7O_4^+$	426.2823	426.2848	5.9
	$C_{20}H_{34}N_7O_5^+$	452.2616	452.2640	5.3

**2**	**fragment formula**	***m/z* calculated**	***m/z* found**	**Error [ppm]**
	$C_6H_{15}N_4O_2^+$	175.1190	175.1184	-3.4
	$C_{11}H_{28}N_7O^+$	274.2350	274.2347	-1.1
	$C_{12}H_{26}N_7O_2^+$	300.2143	300.2139	-1.3
	$C_{15}H_{28}N_5O_3^+$	326.2187	326.2184	-0.9
	$C_{16}H_{34}N_9O_3^+$	400.2779	400.2773	-1.5
	$C_{19}H_{36}N_7O_4^+$	426.2823	426.2818	-1.2
	$C_{20}H_{34}N_7O_5^+$	452.2616	452.2616	-1.1

**3**	**fragment formula**	***m/z* calculated**	***m/z* found**	**Error [ppm]**
	$C_5H_{11}N_4O^+$	143.0927	143.0927	0.0
	$C_6H_{15}N_4O_2^+$	175.1190	175.1193	1.7
	$C_{11}H_{28}N_7O^+$	274.2350	274.2358	2.9
	$C_{12}H_{26}N_7O_2^+$	300.2143	300.2153	3.3
	$C_{15}H_{28}N_5O_3^+$	326.2187	326.2200	4.0
	$C_{16}H_{34}N_9O_3^+$	400.2779	400.2790	2.7
	$C_{19}H_{36}N_7O_4^+$	426.2823	426.2835	2.8
	$C_{20}H_{34}N_7O_5^+$	452.2616	452.2629	2.9
	$C_{26}H_{51}N_{10}O_5^+$	583.4038	583.4056	3.1
	$C_{31}H_{58}N_{13}O_6^+$	708.4628	708.4657	4.1

**4**	**fragment formula**	***m/z* calculated**	**$[M+H]^+$ found**	**Error [ppm]**
	$C_6H_{14}N_5O^+$	172.1193	172.1194	0.6
	$C_6H_{15}N_4O_2^+$	175.1190	175.1193	1.7
	$C_{15}H_{25}N_4O_3^+$	309.1921	309.1931	3.2
	$C_{15}H_{28}N_5O_3^+$	326.2187	326.2200	4.0
	$C_{25}H_{48}N_9O_5^+$	554.3773	554.3790	3.1
	$C_{26}H_{26}N_9O_6^+$	580.3566	580.3585	3.3
	$C_{31}H_{58}N_{13}O_6^+$	708.4628	708.4656	4.0

**5**	**fragment formula**	***m/z* calculated**	***m/z* found**	**Error [ppm]**
	$C_6H_{15}N_4O_2^+$	175.1190	175.1194	2.3
	$C_7H_{16}N_5O^+$	186.1349	186.1355	3.2
	$C_{15}H_{25}N_4O_3^+$	309.1921	309.1935	4.5
	$C_{15}H_{28}N_5O_3^+$	326.2187	326.2202	4.6
	$C_{16}H_{34}N_9O_3^+$	400.2779	400.2790	2.7
	$C_{19}H_{36}N_7O_4^+$	426.2823	426.2834	2.6
	$C_{24}H_{46}N_9O_5^+$	540.3616	540.3636	3.7
	$C_{25}H_{44}N_9O_6^+$	566.3410	566.3431	3.7
	$C_{31}H_{58}N_{13}O_6^+$	708.4628	708.4659	4.4

**6**	**fragment formula**	***m/z* calculated**	***m/z* found**	**Error [ppm]**
	$C_6H_{15}N_4O_2^+$	175.1190	175.1181	-5.1
	$C_8H_{18}N_5O^+$	200.1506	200.1499	-3.5
	$C_{15}H_{28}N_5O_3^+$	326.2187	326.2178	-2.5
	$C_{16}H_{34}N_9O_3^+$	400.2779	400.2772	-1.7
	$C_{19}H_{36}N_7O_4^+$	426.2823	426.2834	-2.1
	$C_{23}H_{44}N_9O_5^+$	526.3460	526.3449	-2.1
	$C_{24}H_{42}N_9O_6^+$	552.3253	552.3240	-2.4
	$C_{31}H_{58}N_{13}O_6^+$	708.4628	708.4616	-1.7

7	fragment formula	*m/z* calculated	*m/z* found	Error [ppm]
	$C_6H_{15}N_4O_2^+$	175.1190	175.1184	-3.4
	$C_7H_{16}N_5O^+$	186.1349	186.1345	-2.5
	$C_{10}H_{27}N_8O^+$	275.2302	275.2300	-0.7
	$C_{11}H_{25}N_8O_2^+$	301.2095	301.2091	-1.3
	$C_{15}H_{28}N_5O_3^+$	326.2187	326.2184	-0.9
	$C_{19}H_{36}N_7O_4^+$	426.2823	426.2823	-1.4
	$C_{20}H_{34}N_7O_5^+$	452.2616	452.2609	-1.5
	$C_{23}H_{45}N_{10}O_5^+$	541.3569	541.3563	-1.1
	$C_{24}H_{43}N_{10}O_6^+$	567.3362	567.3355	-1.2

8	fragment formula	*m/z* calculated	*m/z* found	Error [ppm]
	$C_6H_{14}N_5O^+$	172.1193	172.1192	-0.6
	$C_6H_{15}N_4O_2^+$	175.1190	175.1190	0.0
	$C_{10}H_{27}N_8O^+$	275.2302	275.2310	2.9
	$C_{11}H_{25}N_8O_2^+$	301.2095	301.2100	1.7
	$C_{15}H_{28}N_5O_3^+$	326.2187	326.2194	2.1
	$C_{19}H_{36}N_7O_4^+$	426.2823	426.2828	1.2
	$C_{20}H_{34}N_7O_5^+$	452.2616	452.2616	1.1
	$C_{24}H_{47}N_{10}O_5^+$	555.3725	555.3736	2.0
	$C_{25}H_{45}N_{10}O_6^+$	581.3518	581.3526	1.4
	$C_{30}H_{57}N_{14}O_6$	709.4580	709.4597	2.4

9	fragment formula	*m/z* calculated	*m/z* found	Error [ppm]
	$C_5H_{12}N_5O^+$	158.1036	158.1036	**0.0**
	$C_6H_{15}N_4O_2^+$	175.1190	175.1194	2.3
	$C_{15}H_{28}N_5O_3^+$	326.2187	326.2200	4.0
	$C_{19}H_{36}N_7O_4^+$	426.2823	426.2834	2.6
	$C_{20}H_{34}N_7O_5^+$	452.2616	452.2628	2.7
	$C_{25}H_{49}N_{10}O_5^+$	569.3882	569.3897	2.6
	$C_{26}H_{47}N_{10}O_6^+$	595.3675	595.3695	3.4
	$C_{30}H_{57}N_{14}O_6$	709.4580	709.4609	4.1

10	fragment formula	*m/z* calculated	*m/z* found	Error [ppm]
	$C_7H_{20}N_5^+$	174.1713	174.1713	0.0
	$C_6H_{15}N_4O_2^+$	175.1190	175.1190	-0.6
	$C_{15}H_{25}N_4O_3^+$	309.1921	309.1925	1.3
	$C_{15}H_{28}N_5O_3^+$	326.2187	326.2190	0.9
	$C_{15}H_{28}N_5O_3^+$	326.2187	326.2190	0.9
	$C_{25}H_{19}N_2O_7^+$	459.1187	459.1187	0.0
	$C_{29}H_{27}N_4O_8^+$	559.1823	559.1815	-1.4
	$C_{37}H_{44}N_9O_9^+$	758.3247	758.3257	-1.3
	$C_{31}H_{58}N_{13}O_6^+$	708.4628		

<u>Compound 1</u>: H$_2$N-Dab^U(*h*Arg)-Dab-Oic-Arg-OH

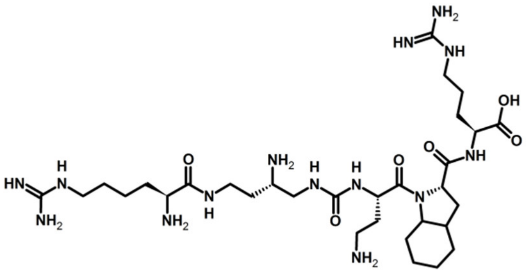

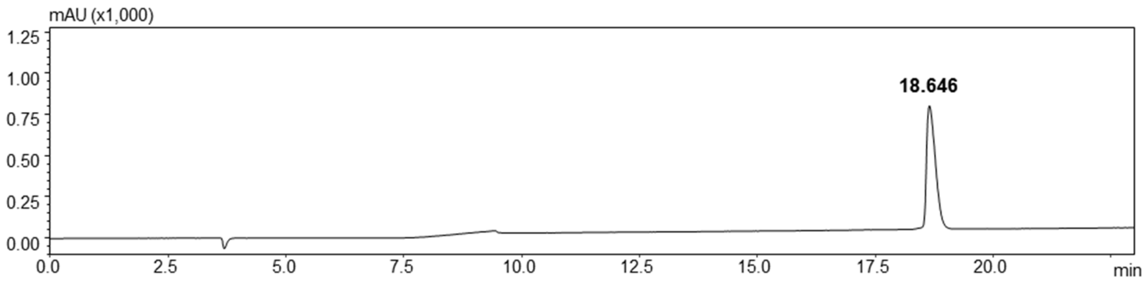

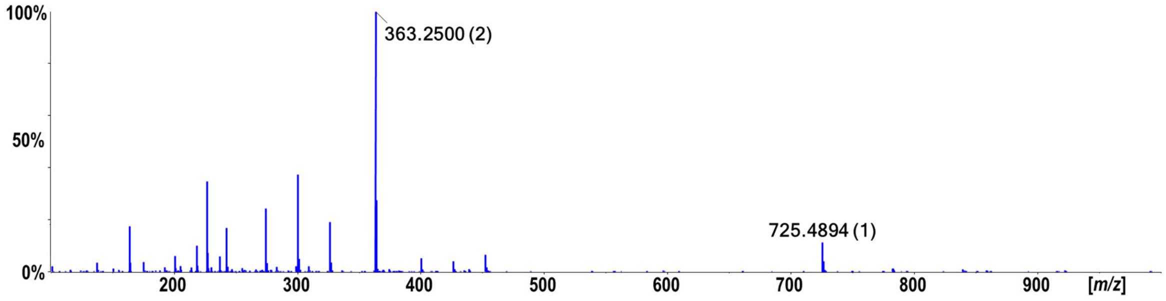

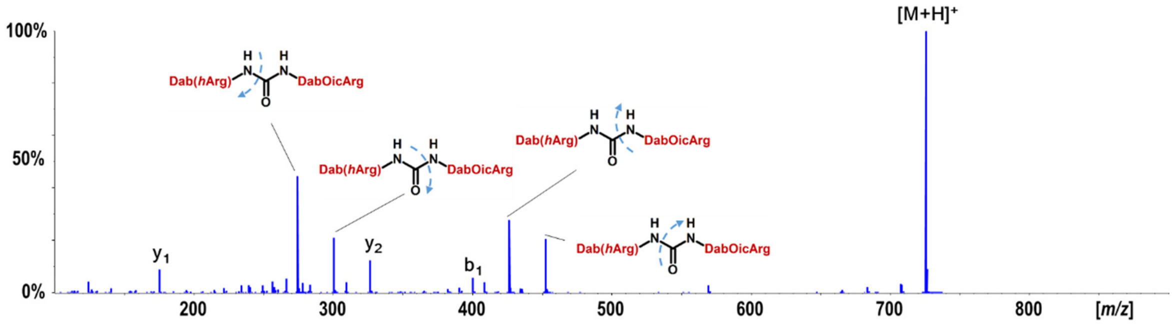

Figure S6. HPLC chromatogram of hybrid **1** at 200 nm, HRMS spectrum (value in bracket represent the charge state) and MS/MS fragmentation of $[M+H]^+$.

Compound 2: H_2N-Orn^U(Arg)-Dab-Oic-Arg-OH

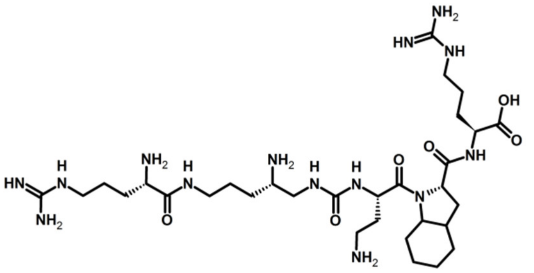

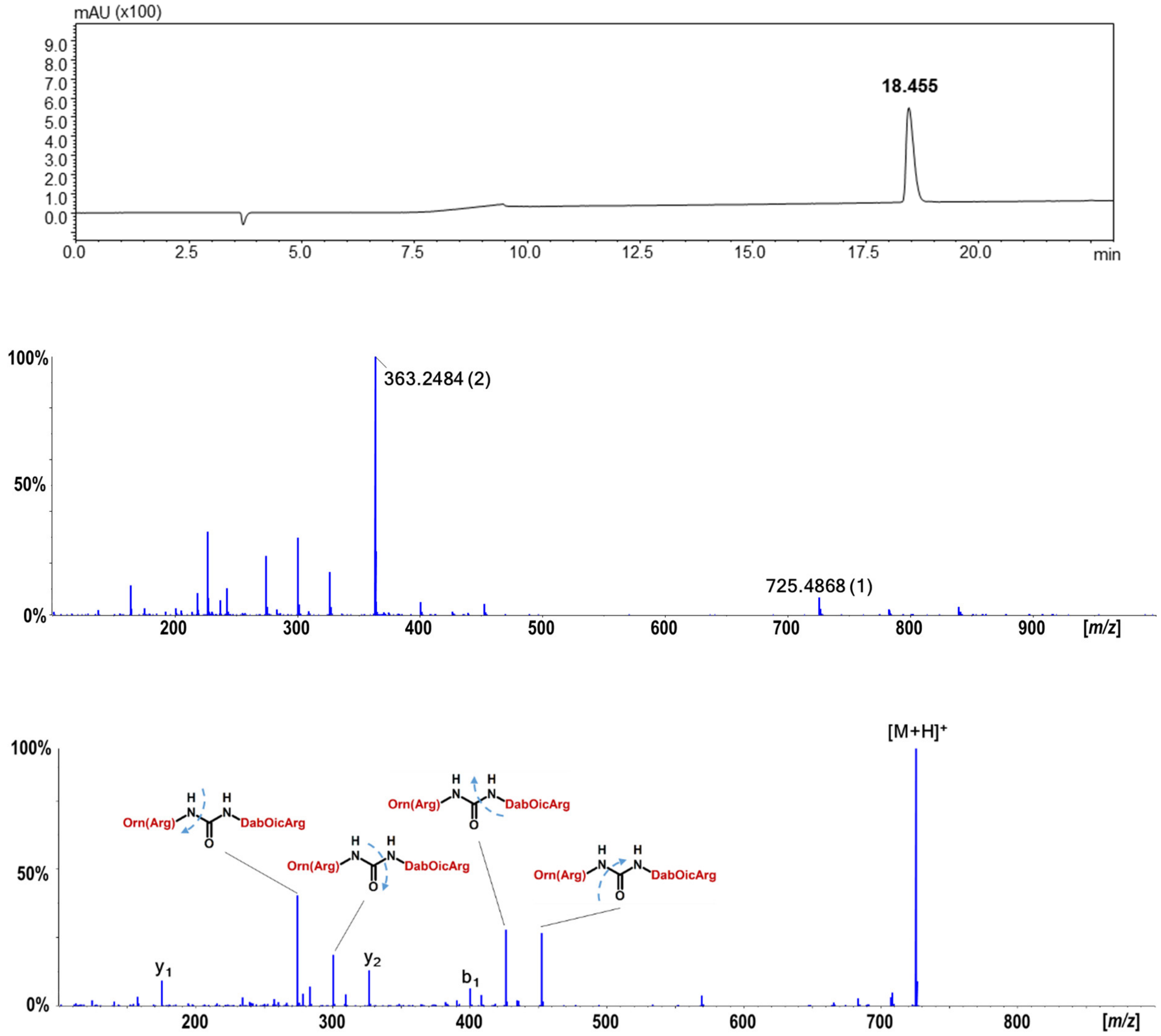

Figure S7. HPLC chromatogram of hybrid **2** at 200 nm, HRMS spectrum (value in bracket represent the charge state) and MS/MS fragmentation of $[M+H]^+$.

Compound 3: H_2N-Lys^{U}(gDab)-Dab-Oic-Arg-OH

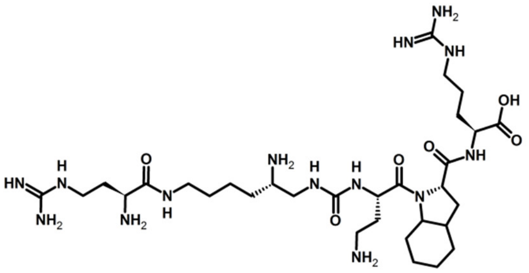

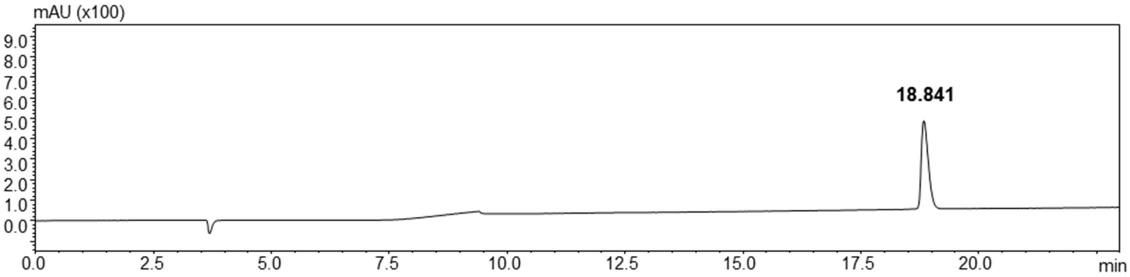

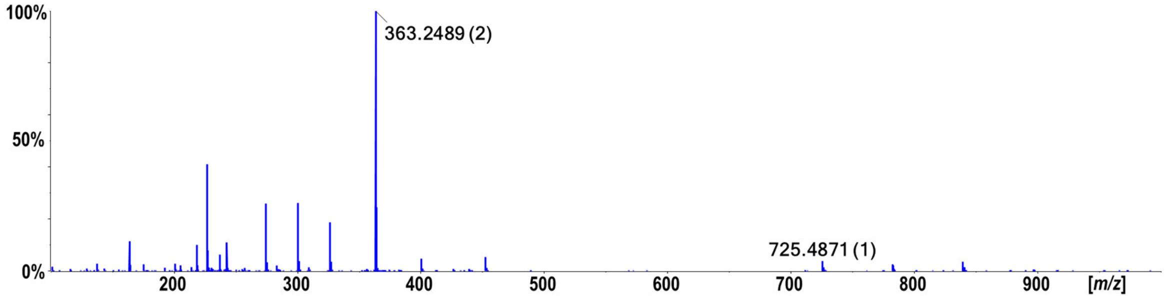

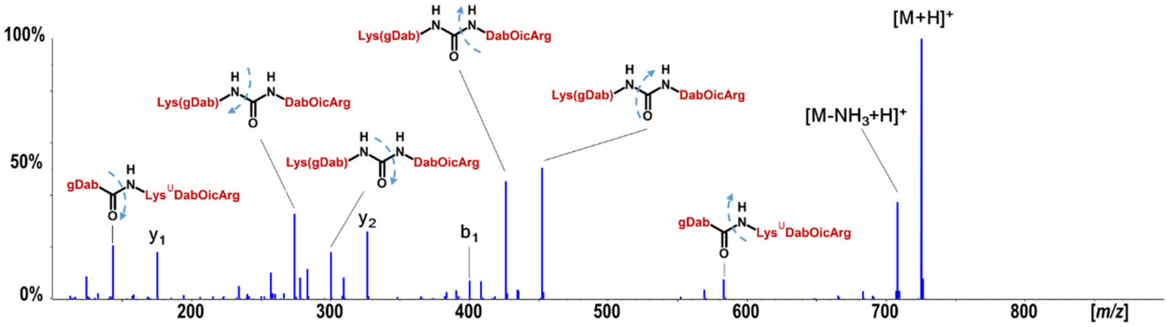

Figure S8. HPLC chromatogram of hybrid **3** at 200 nm, HRMS spectrum (value in bracket represent the charge state) and MS/MS fragmentation of $[M+H]^+$.

<u>Compound 4</u>: H_2N-Lys(gDab^U)-Dab-Oic-Arg-OH

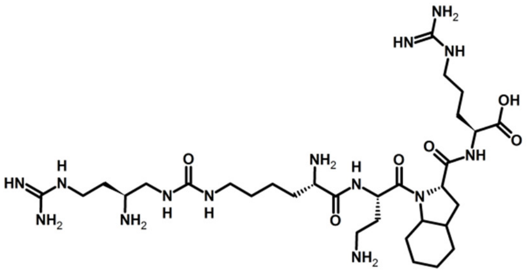

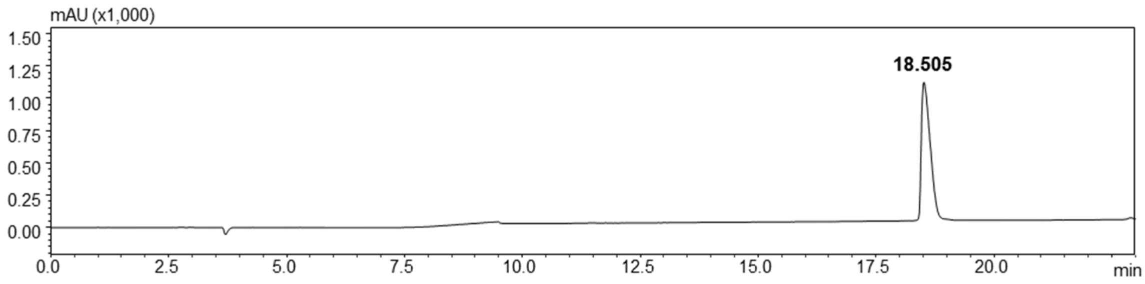

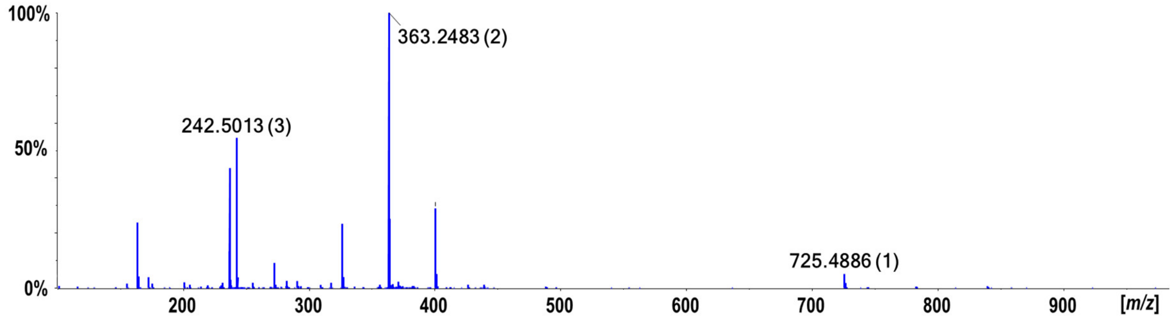

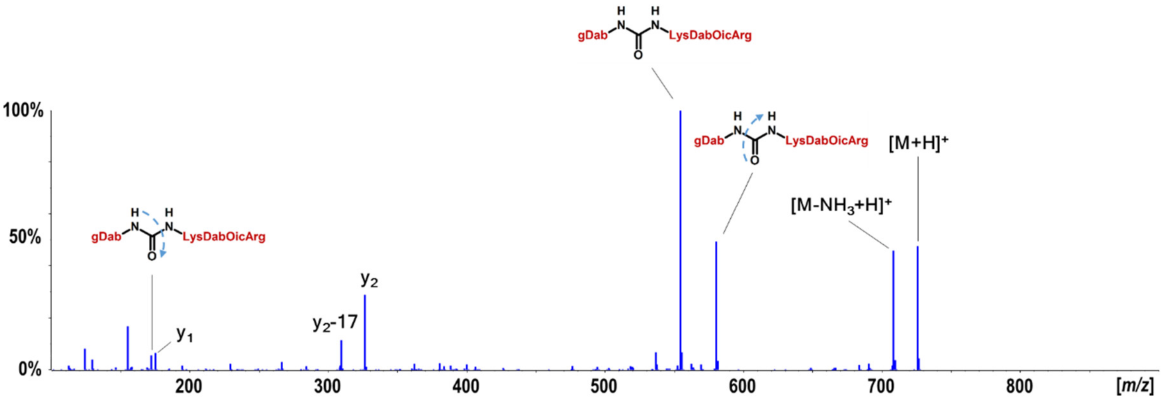

Figure S9. HPLC chromatogram of hybrid **4** at 200 nm, HRMS spectrum (value in bracket represent the charge state) and MS/MS fragmentation of $[M+H]^+$.

Compound 5: H_2N-Orn(Arg^U)-Dab-Oic-Arg-OH

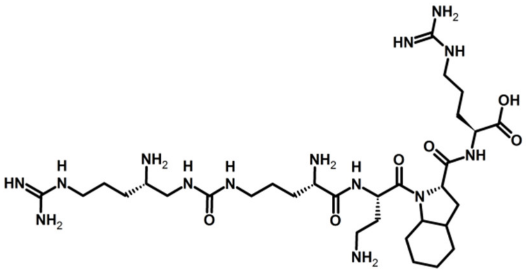

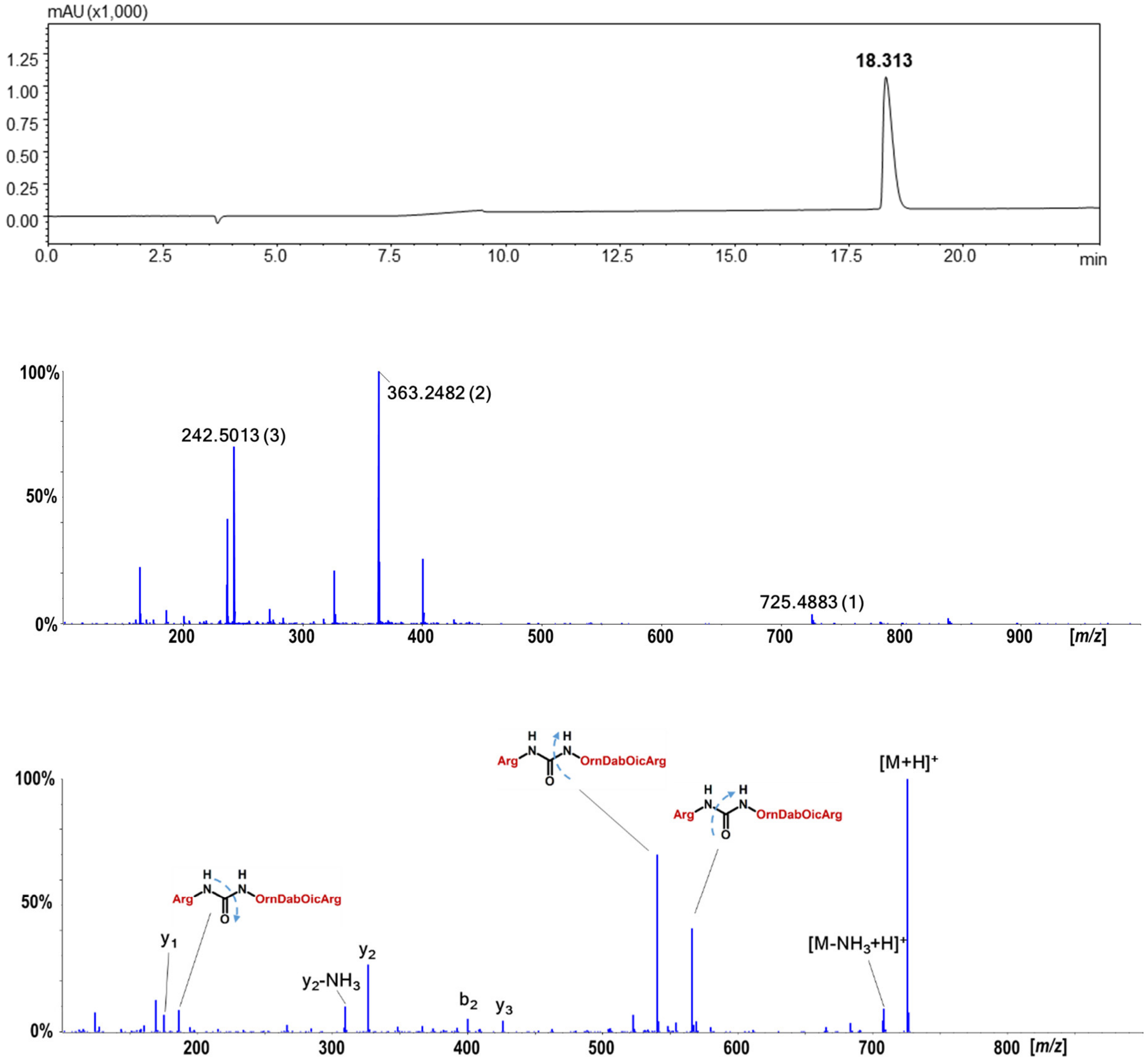

Figure S10. HPLC chromatogram of hybrid **5** at 200 nm, HRMS spectrum (value in bracket represent the charge state) and MS/MS fragmentation of $[M+H]^+$.

Compound 6: **H_2N-Dab(*h*Arg^{U})-Dab-Oic-Arg-OH**

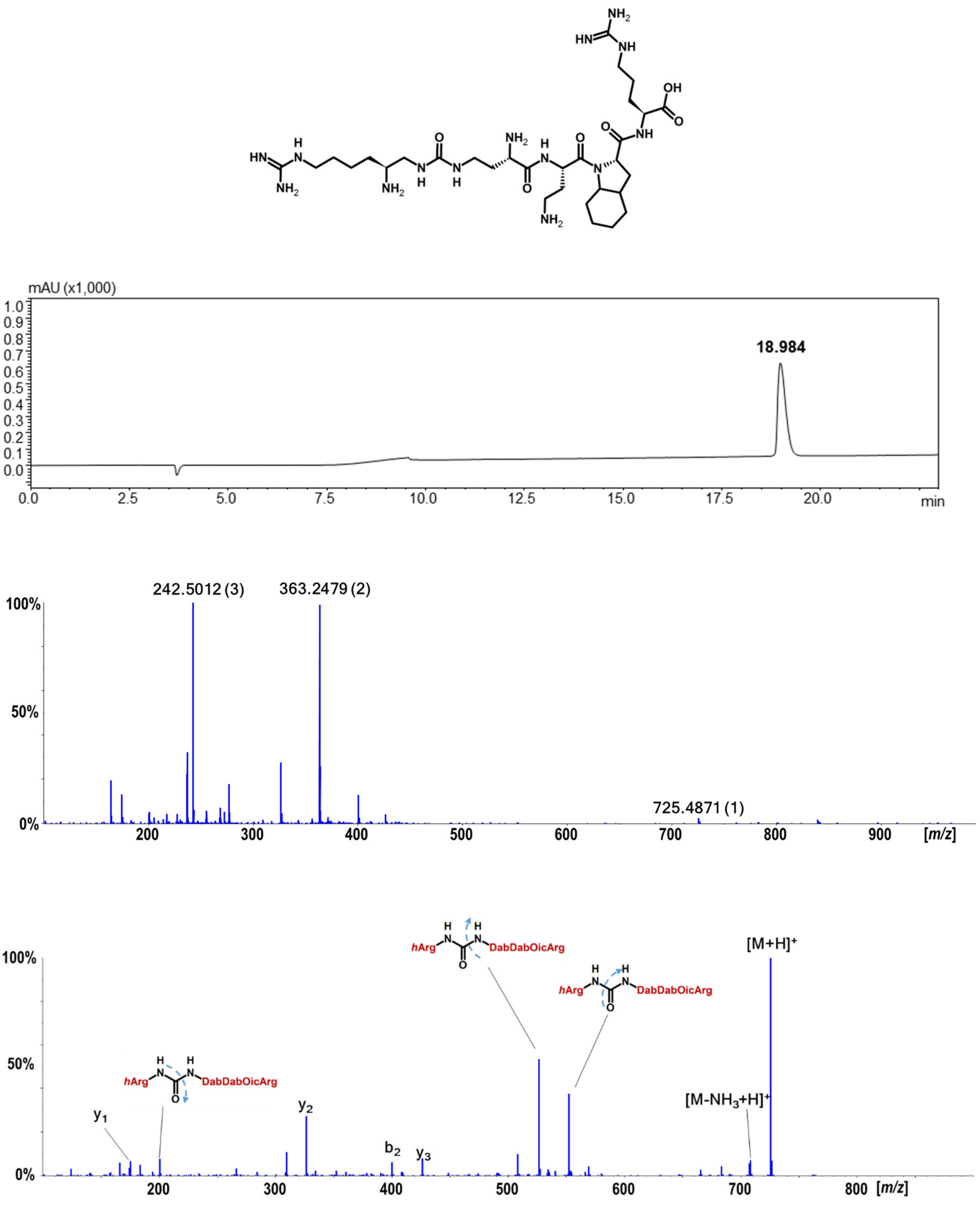

Figure S11. HPLC chromatogram of hybrid **6** at 200 nm, HRMS spectrum (value in bracket represent the charge state) and MS/MS fragmentation of $[M+H]^{+}$.

Compound 7: H₂N-Dap^U(Arg^U)-Dab-Oic-Arg-OH

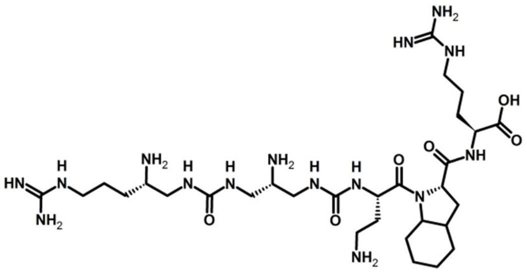

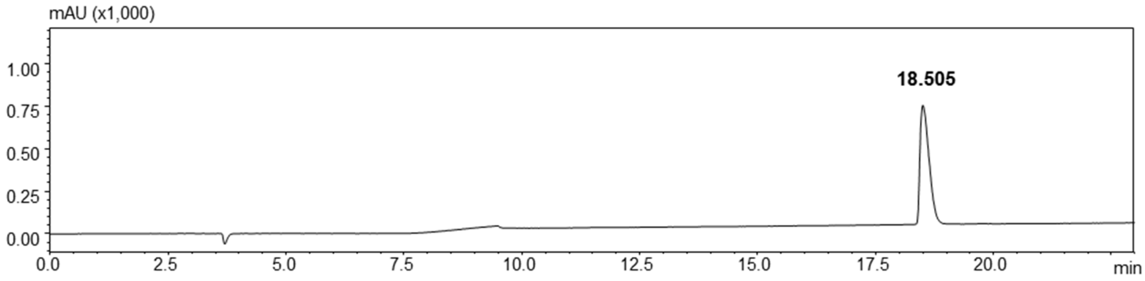

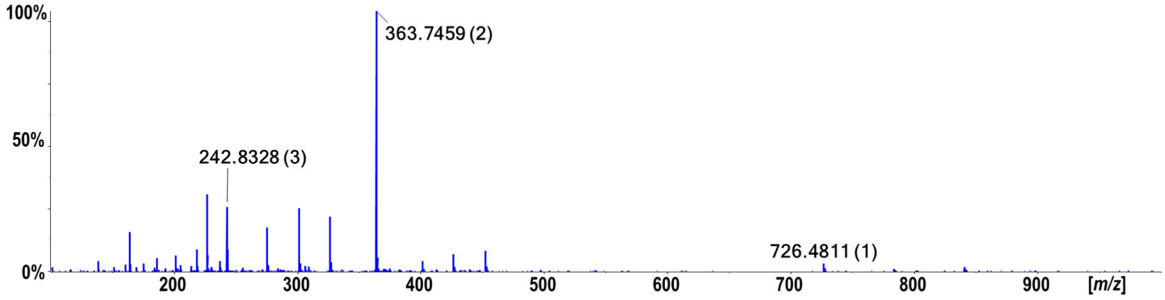

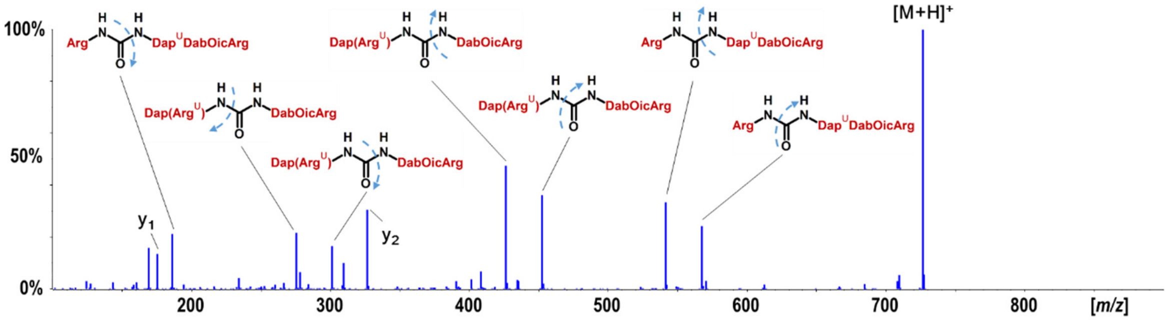

Figure S12. HPLC chromatogram of hybrid **7** at 200 nm, HRMS spectrum (value in bracket represent the charge state) and MS/MS fragmentation of $[M+H]^+$.

Compound 8: H₂N-Dab[U](gDab[U])-Dab-Oic-Arg-OH

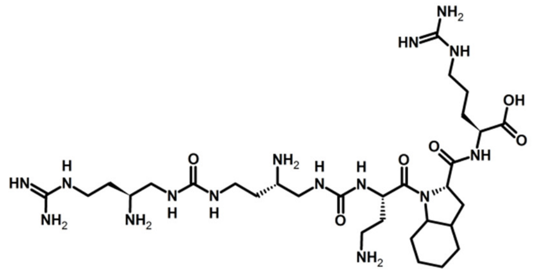

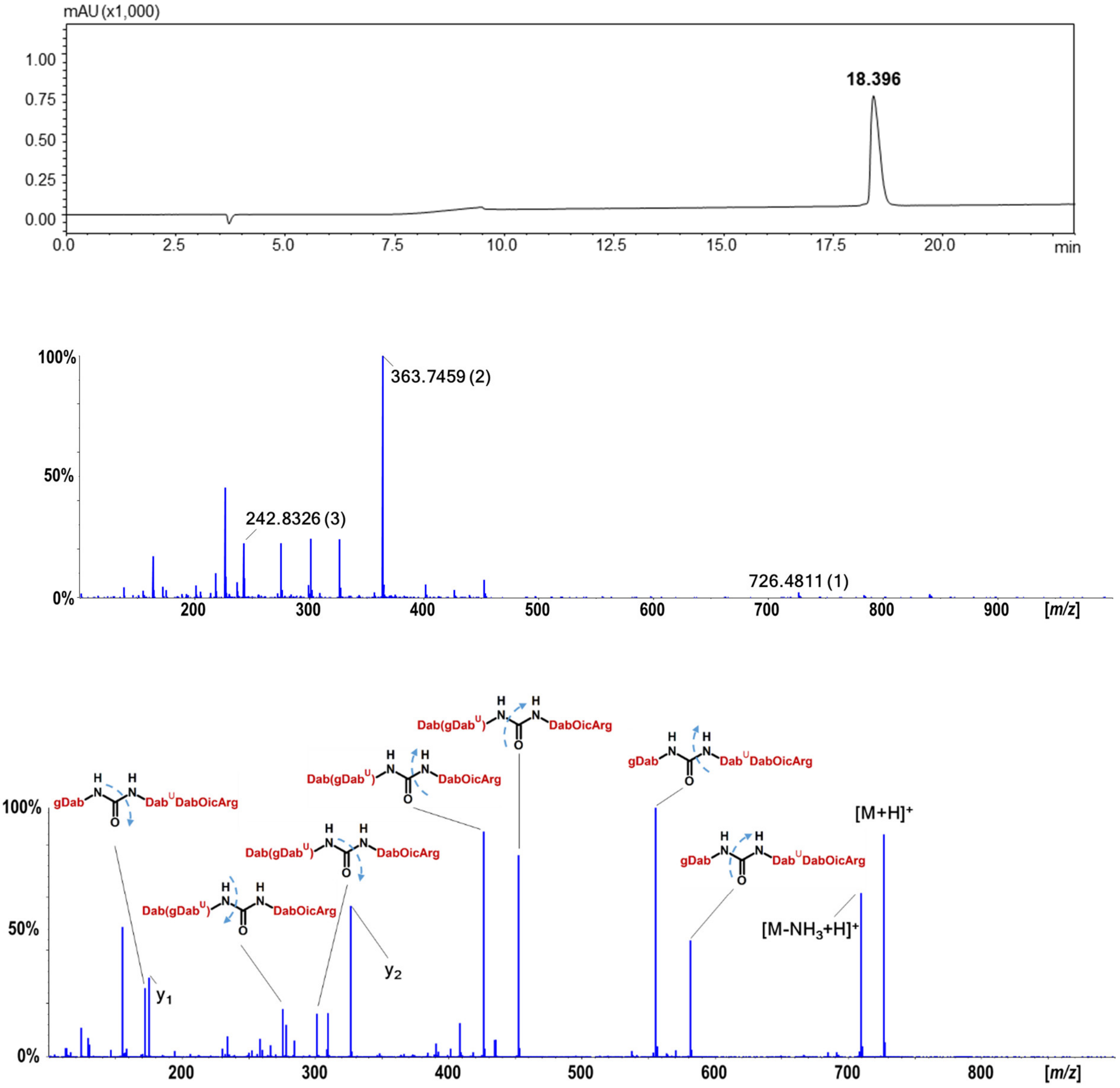

Figure S13. HPLC chromatogram of hybrid **8** at 200 nm, HRMS spectrum (value in bracket represent the charge state) and MS/MS fragmentation of $[M+H]^+$.

Compound 9: H_2N-Orn^U(gDap^U)-Dab-Oic-Arg-OH

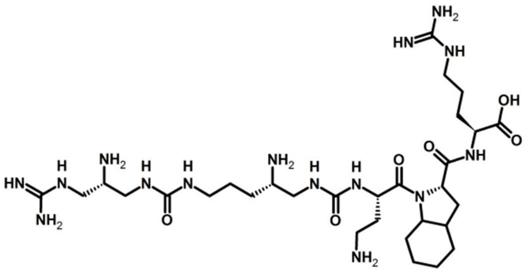

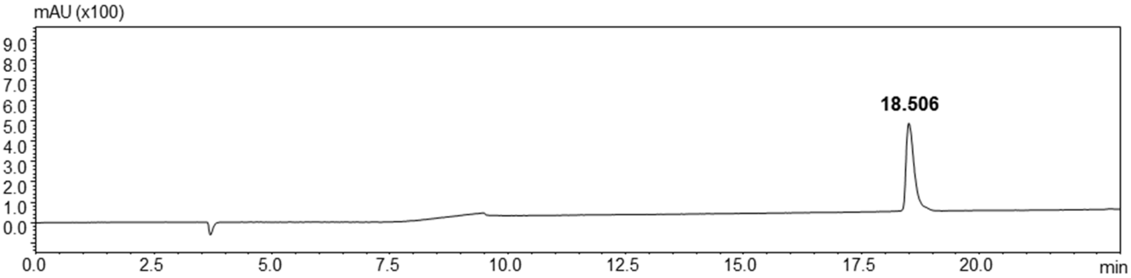

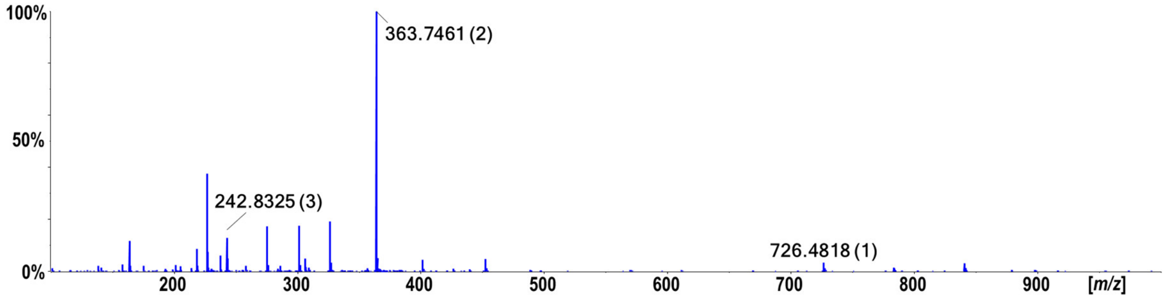

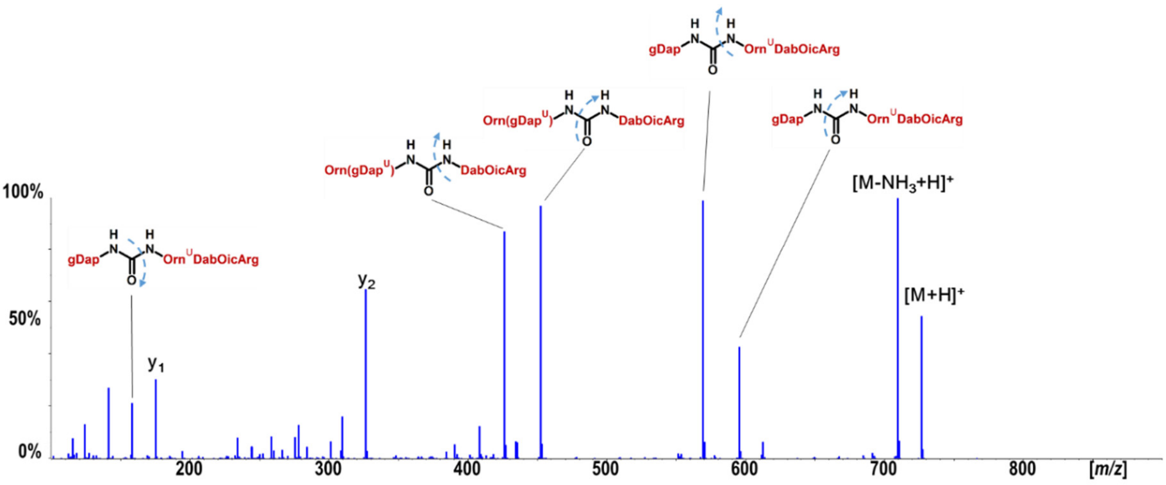

Figure S14. HPLC chromatogram of hybrid **9** at 200 nm, HRMS spectrum (value in bracket represent the charge state) and MS/MS fragmentation of $[M+H]^+$.

<u>Compound 10</u>: 5/6-FAM-Dab(*h*Arg^{U})-Dab-Oic-Arg-OH

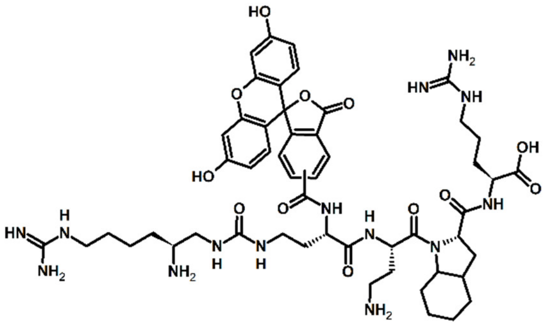

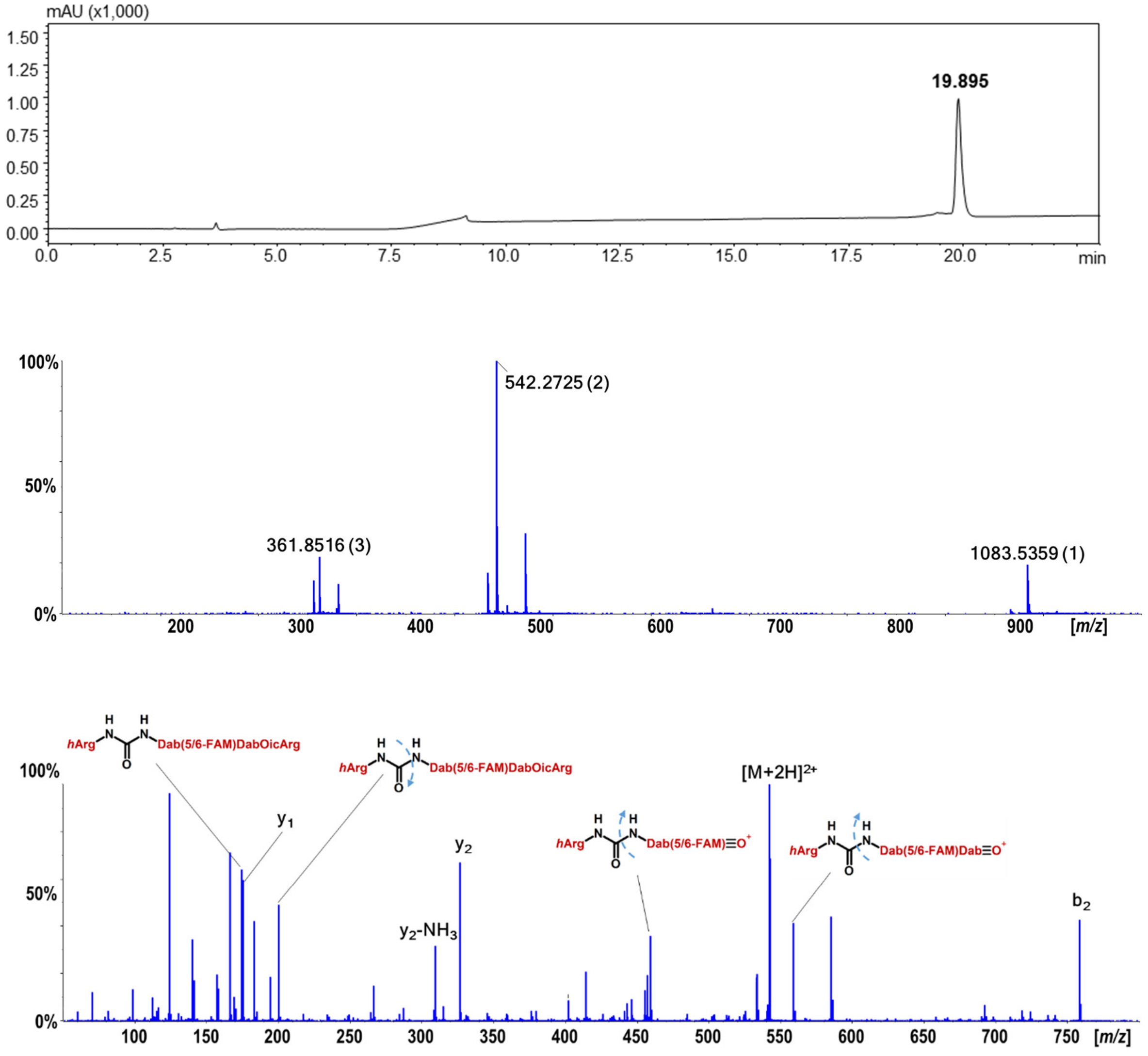

Figure S15. HPLC chromatogram of hybrid **10** at 200 nm, HRMS spectrum (value in bracket represent the charge state) and MS/MS fragmentation of $[M+H]^{+}$.

5. Dose-response curves of urea-peptide hybrids. The concentration-dependent inhibitory dose-curve data were plotted as percentage inhibition normalized to controls with applied curve fits calculated using GraphPad Prism (Version-5.01, GraphPad software). Data are presented as log(inhibitor) versus normalized response-variable slope. Error bars are representing means +/- SEM for 2 or 3 independent experiments. Top and bottom plateau of each curve was constrained to be a constant value equal to the mean of the positive control values and to the mean of the NS values, respectively.

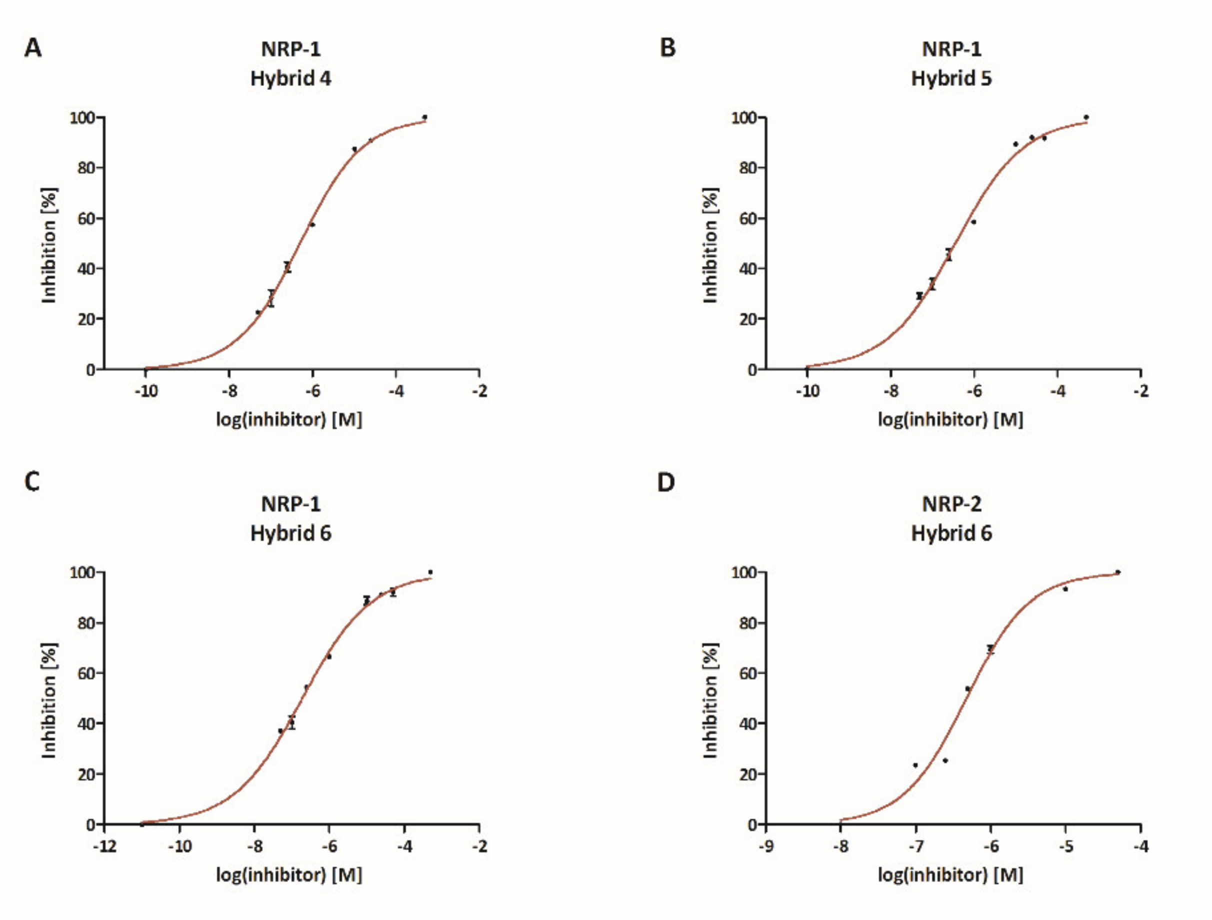

Figure S16. Dose-response curves of the best urea-peptide hybrids **4**-**6**.

6. Inhibitory effect on VEGF-A_{165}/NRP-1 complex formation of hybrid 10.

Table S4. Urea-peptide hybrid **10** inhibitory effect on VEGF-A_{165}/NRP-1 complex formation.

Compound	Sequence	$logIC_{50}$ ± SEM	IC_{50} [µM]
**10**	5(6)-FAM-Dab(*h*Arg^{U})Dab-Oic-Arg-OH	-5.39 ± 0.03	4.04

R^2=0.99; compound was tested in the concentrations range 0.05 – 10 µM.

7. Analytical data of serum degradation. Analysis of plasma degradation products was carried out by HPLC-ESI-Q-TOF-MS with a Shimadzu Nexera HPLC system consisting of LC-30AD quaternary pump (LPGE), autosampler SIL-30AC and CTO30A column oven controlled by CBM20Alite controller. HPLC system was coupled to TripleTOF®5600 mass spectrometer equipped with a DuoSpray™ ion source. Chromatographic separation was achieved on Waters ACQUITY UPLC CSH130 C18 1.7µ column (150 × 2.1 mm) at 40˚C. Mobile phases were 10 mM ammonium formate and 0.1% FA in water (phase A), 10 mM ammonium formate and 0.1% FA in 80% acetonitrile (phase B) at total flow rate of 0.3 mL/min.

Elution was performed with a gradient as follows: 0-1min 0%B, 1-7 min 0-30% B, 7-8 min 30-100% B, 8-9 min 10% B. The column was reconditioned at 0%B for 7 min, resulting in total time of analysis t=17min. The injection volume was 30 µL. Retention time is only given for untouched compound and identified degradation products. Other signals were not identified and are supposedly from plasma and its natural degradation in time.

The electrospray ionization (ESI) was operated in positive mode. Curtain gas (CUR) was set to 25 psi. Nebulizing gas (GS1) was set to 30 psi and drying gas (GS2) was set to 40 psi. Needle voltage (ISVF) was set to 5kV and temperature (TEM) was set to 500ºC. Declustering potential was set to 80V.

Mass spectrometer was operated in mixed HRMS/SWATH-MS/MS acquisition mode with total cycle time of 510 ms. MS1 experiment was performed in TOFMS mode in mass range 50-1000 with 100 ms accumulation time. MS2 experiment consisted of 12 independent 50 Da SWATH windows covering mass range of 150-750, with 30 ms accumulation time per each window. To induce fragmentation, collision energy voltage (CE) was set to 35 V and collision energy spread voltage (CES) was set to 15 V.

Table S5. Identified substrate and products of parent peptide after 8h of incubation in human serum.

RT [min]	$[M+H]^+_{found}$	$[M+H]^+_{calc.}$	Error [ppm]	Formula	Predicted structure
5.27	724.4939	724.4941	-0.3	$C_{32}H_{62}N_{13}O_6^+$	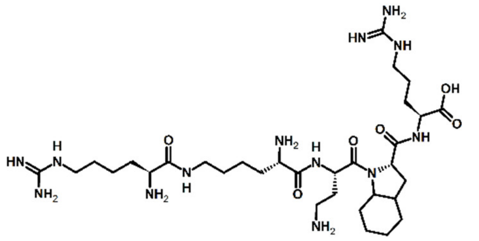
5.39	568.3919	569.3929	-1.8	$C_{26}H_{50}N_9O_5^+$	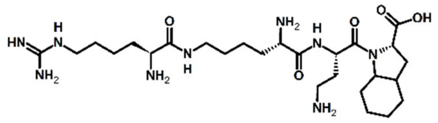
2.87	554.3761	554.3773	-2.2	$C_{25}H_{48}N_9O_5^+$	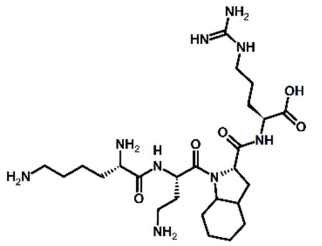
3.54	426.2814	426.2823	-2.1	$C_{19}H_{36}N_7O_4^+$	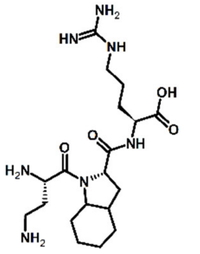
6.06	170.1177	170.1176	0.6	$C_9H_{16}NO_2^+$	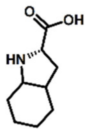

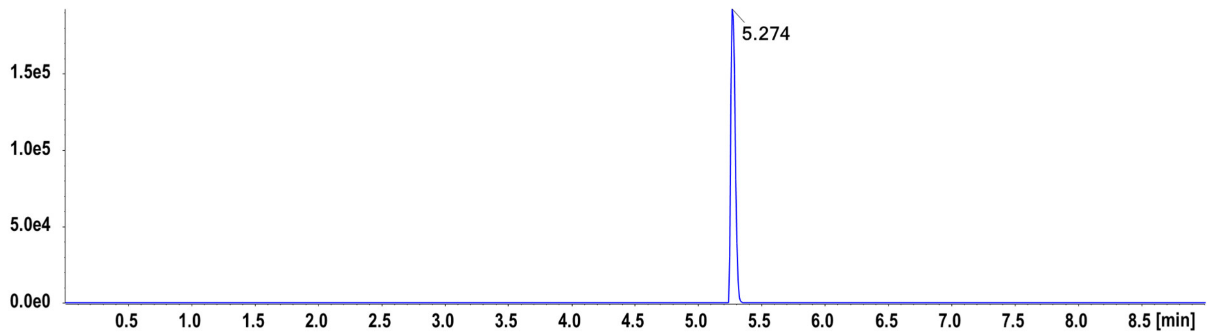

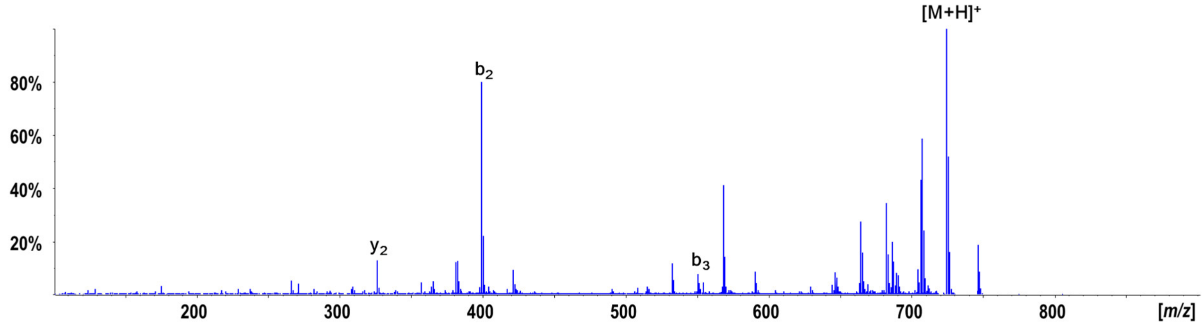

Fragment formula	*m/z* calculated	*m/z* found	Error [ppm]
$C_{15}H_{28}N_5O_3^+$	326.2187	326.2186	-0.3
$C_{17}H_{35}N_8O_3^+$	399.2827	399.2825	-0.5
$C_{26}H_{48}N_9O_4^+$	550.3825	550.3840	-2.7

Figure S17. XIC, MS/MS spectra and MS/MS analytical data of parent peptide after 8h degradation in human serum.

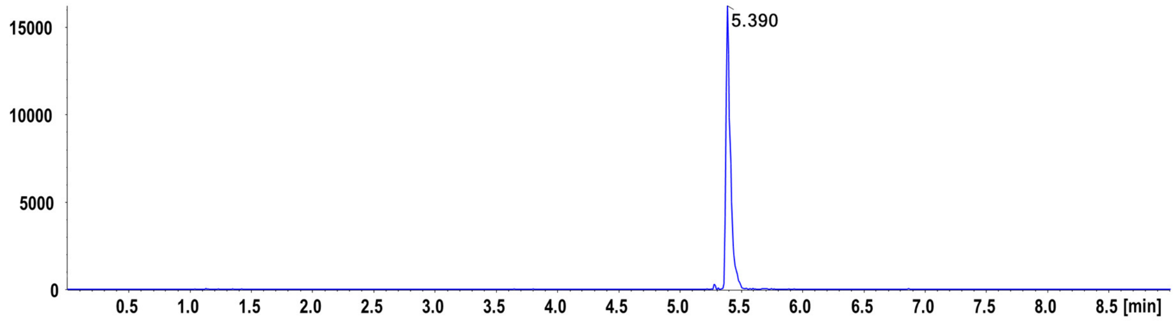

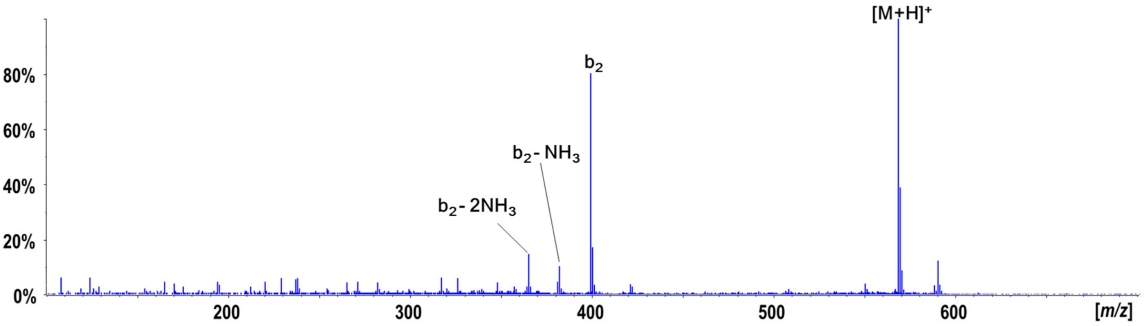

Fragment formula	*m/z* calculated	*m/z* found	Error [ppm]
$C_{17}H_{29}N_6O_3^+$	365.2296	365.2276	-5.5
$C_{17}H_{32}N_7O_3^+$	382.2544	382.2561	-4.4
$C_{17}H_{35}N_8O_3^+$	399.2827	399.2813	-3.5

Figure S18. XIC, MS/MS spectra and MS/MS analytical data of enzymatic hydrolysis product 568.3919 *m/z*.

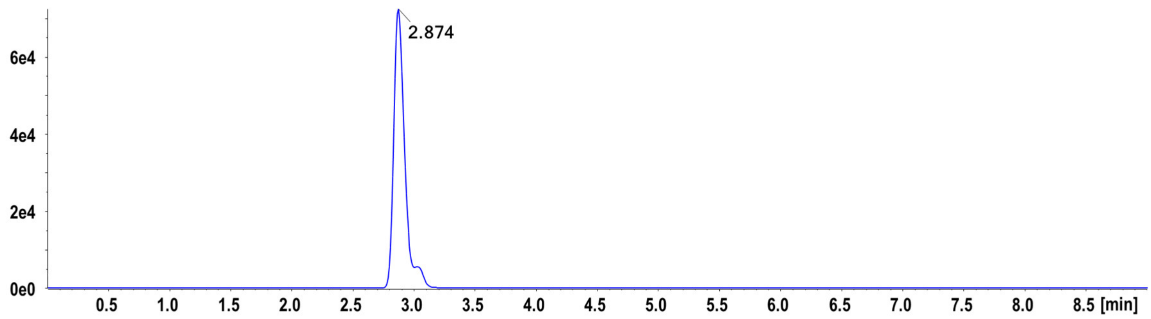

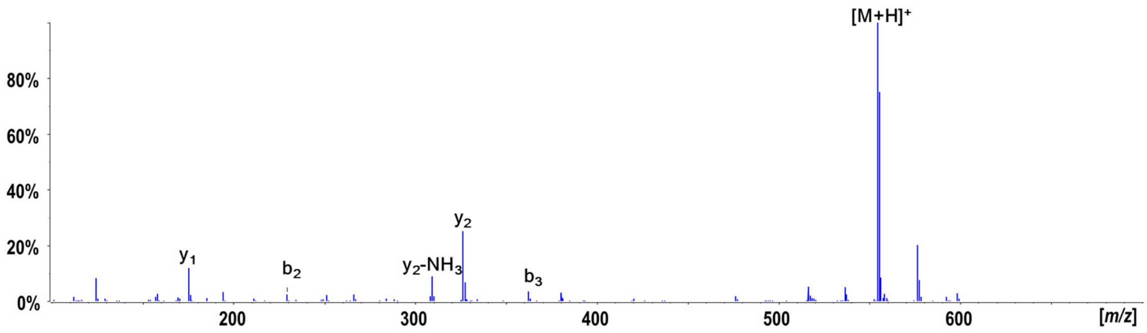

Fragment formula	*m/z* calculated	*m/z* found	Error [ppm]
$C_6H_{16}N_4O_2^+$	175.1190	175.1184	-3.4
$C_{10}H_{21}N_4O_2^+$	229.1659	229.1649	-4.4
$C_{15}H_{25}N_4O_3^+$	309.1921	309.1924	1.0
$C_{15}H_{28}N_5O_3^+$	326.2187	326.2187	0.0
$C_{19}H_{34}N_5O_3^+$	380.2656	380.2648	-2.1

Figure S19. XIC, MS/MS spectra and MS/MS analytical data of 554.3761 *m/z*.

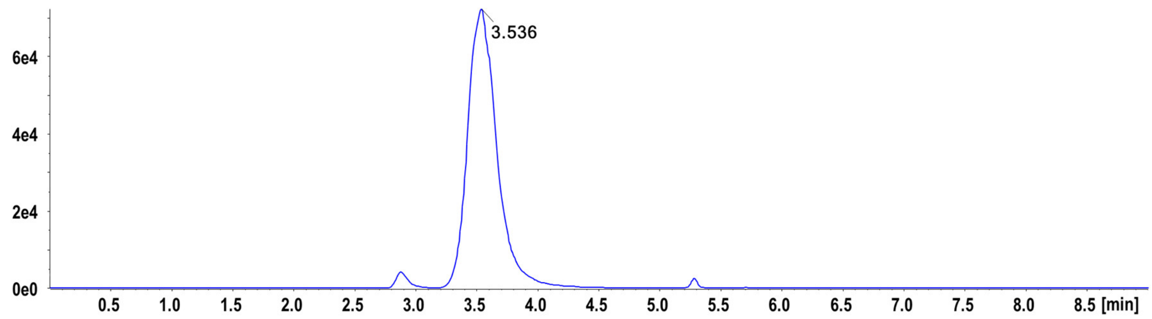

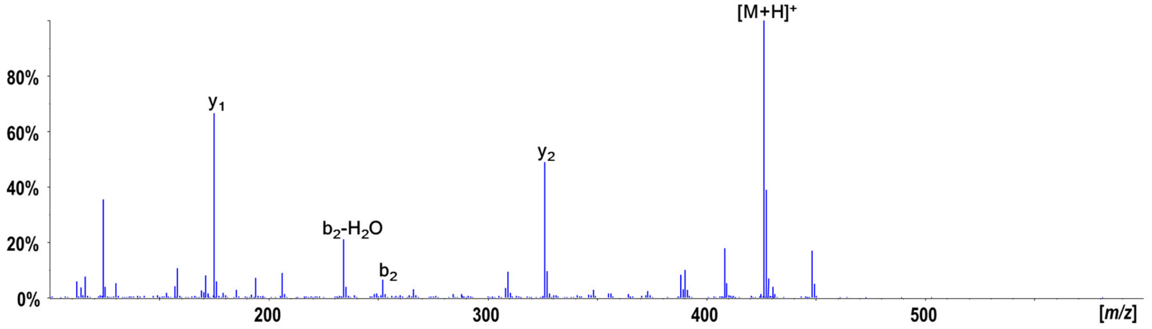

Fragment formula	*m/z* calculated	*m/z* found	Error [ppm]
$C_6H_{16}N_4O_2^+$	175.1190	175.1186	-2.3
$C_{13}H_{20}N_3O^+$	234.1601	234.1594	-3.0
$C_{13}H_{22}N_3O_2^+$	252.1707	252.1699	-3.2
$C_{15}H_{28}N_5O_3^+$	326.2187	326.2181	1.8

Figure S20. XIC, MS/MS spectra and MS/MS analytical data of 426.2814 *m/z*.

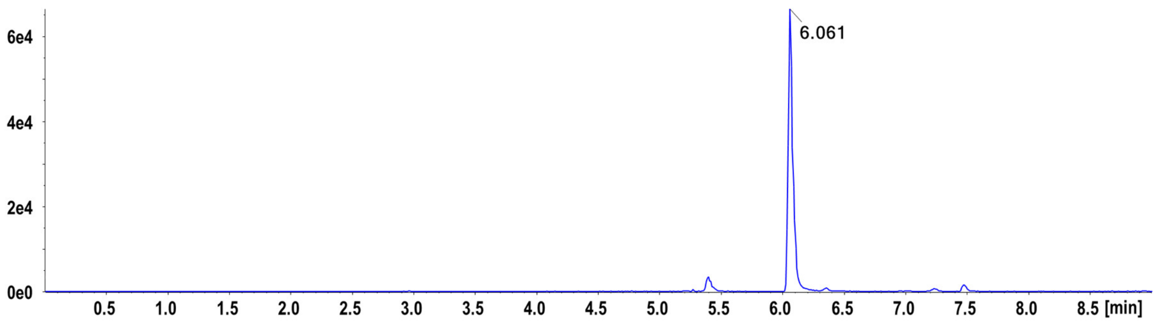

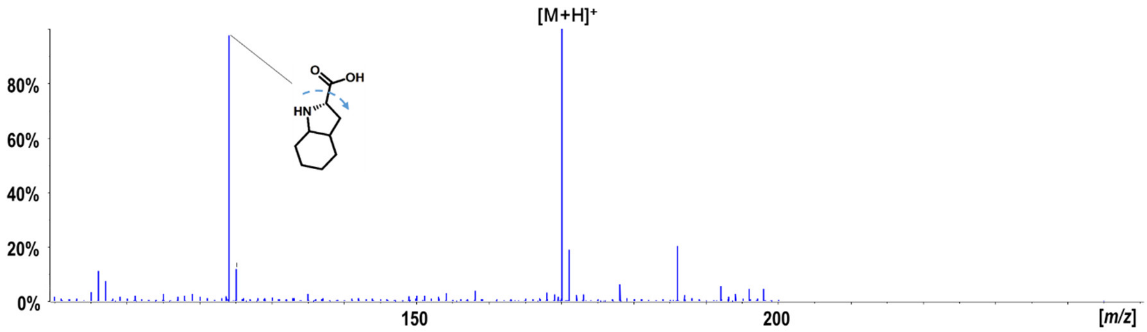

Fragment formula	*m/z* calculated	*m/z* found	Error [ppm]
$C_8H_{14}N^+$	124.1121	124.1119	-1.6

Figure S21. XIC, MS/MS spectra and MS/MS analytical data of 170.1177 *m/z*.

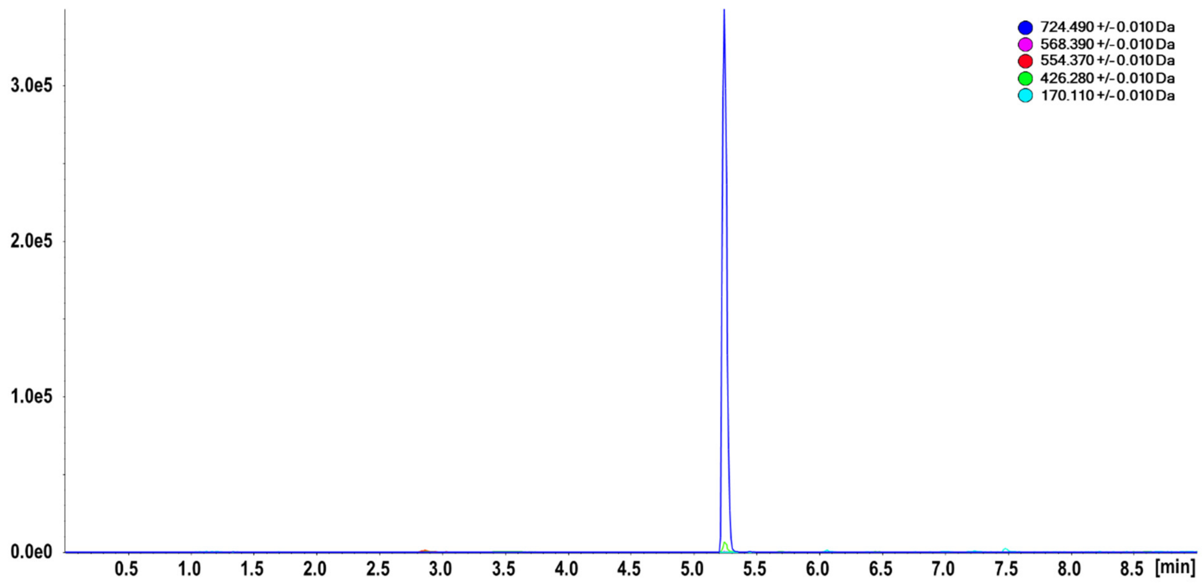

Figure S22. XICs of parent peptide and its degradation products after 0h degradation time.

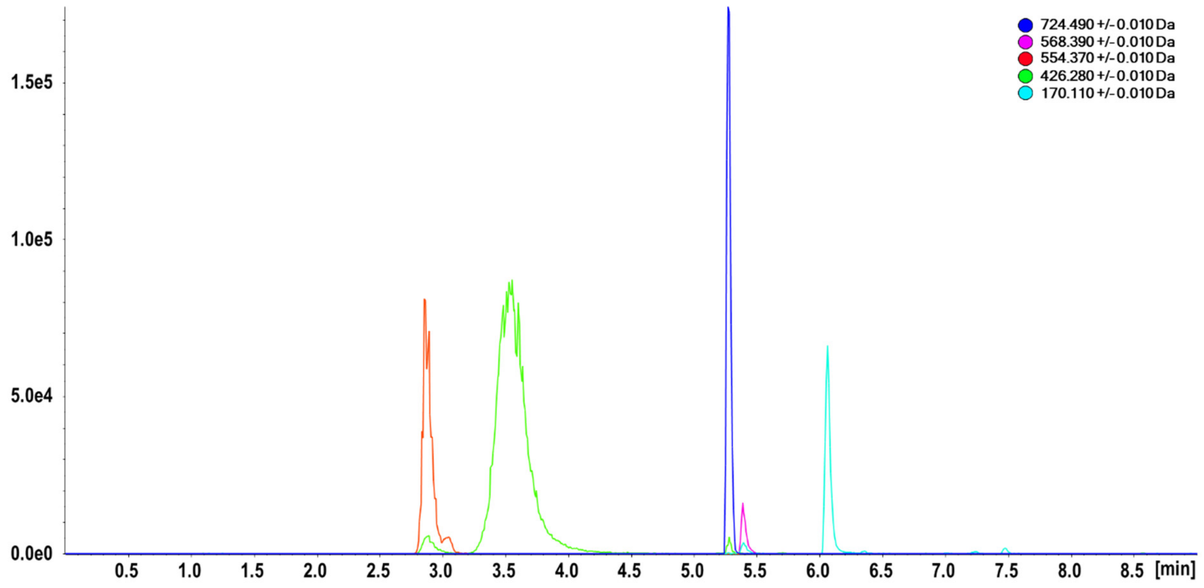

Figure S23. XICs of parent peptide and its degradation products after 8h degradation time.

Table S6. Identified substrate and products of hybrid **3** after 96h of incubation in human serum.

RT [min]	$[M+H]^+_{found}$	$[M+H]^+_{calc.}$	Error [ppm]	Formula	Predicted structure
5.27	725.4879	725.4893	-1.9	$C_{31}H_{60}N_{14}O_6^+$	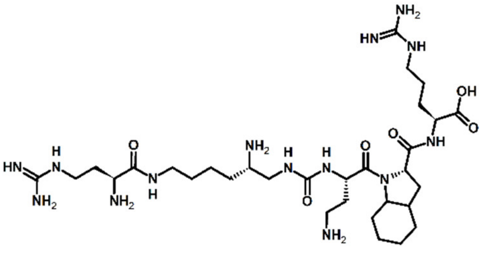
5.43	569.3880	569.3882	-0.4	$C_{25}H_{49}N_{10}O_5^+$	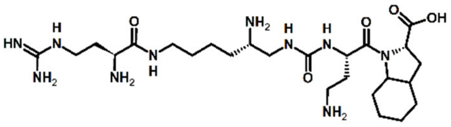
5.28	583.4024	583.4038	-2.4	$C_{25}H_{49}N_{10}O_5^+$	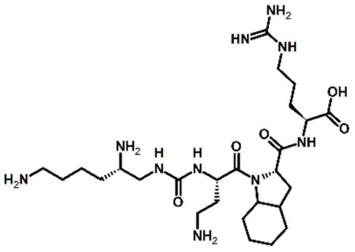
5.46	427.3031	427.3027	0.9	$C_{29}H_{39}N_6O_4^+$	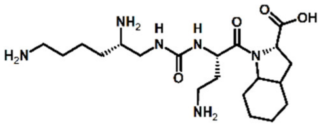
6.06	170.1176	170.1176	0.0	$C_9H_{16}NO_2^+$	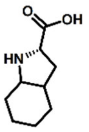

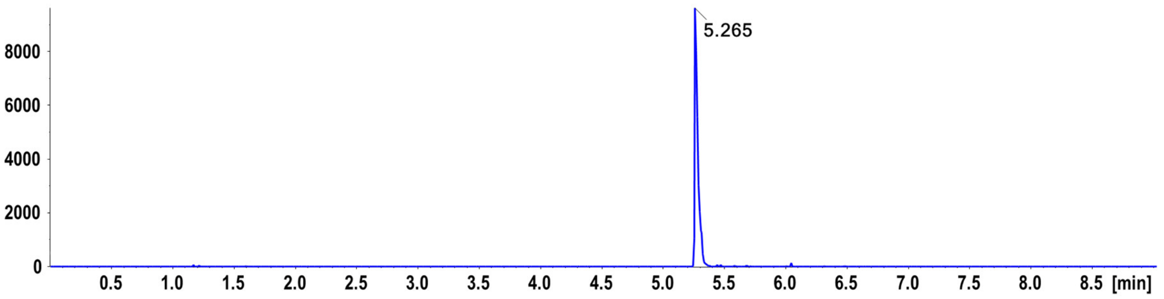

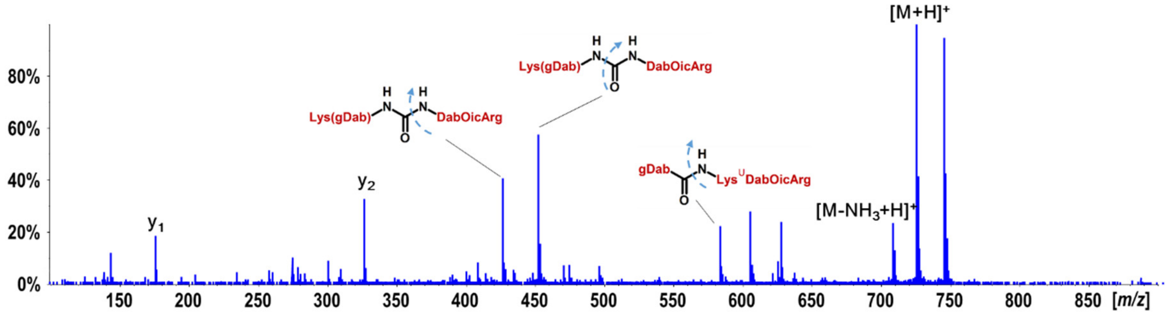

Fragment formula	*m/z* calculated	*m/z* found	Error [ppm]
$C_6H_{15}N_4O_2^+$	175.1190	175.1188	-1.1
$C_{15}H_{28}N_5O_3^+$	326.2187	326.2173	-4.3
$C_{19}H_{36}N_7O_4^+$	426.2823	426.2819	-0.9
$C_{20}H_{34}N_7O_5^+$	452.2616	452.2610	-1.3
$C_{26}H_{51}N_{10}O_5^+$	583.4038	583.4056	3.1
$C_{31}H_{58}N_{13}O_6^+$	708.4628	708.4628	-2.5

Figure S24. XIC, MS/MS spectra and MS/MS analytical data of compound **3** after 96h degradation in human serum.

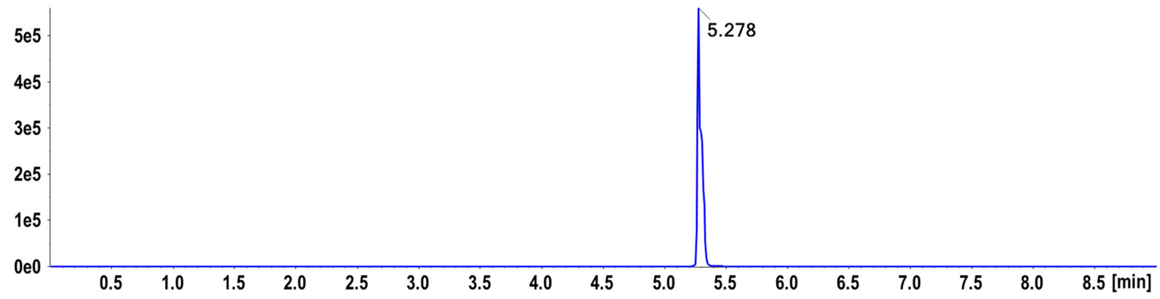

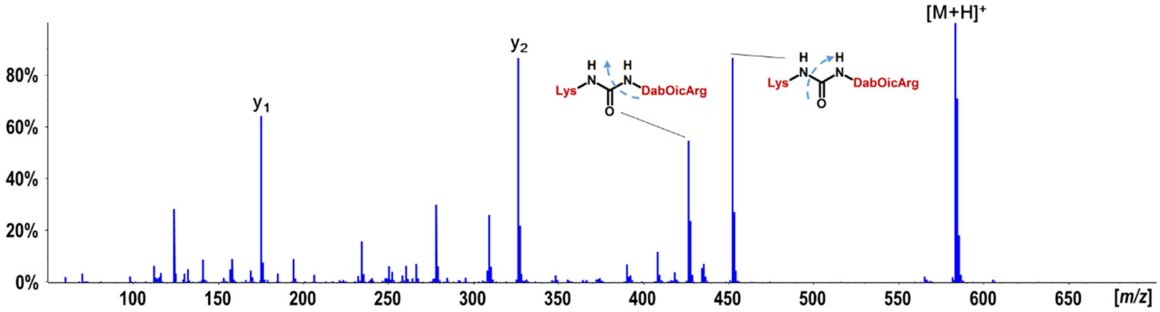

Fragment formula	*m/z* calculated	*m/z* found	Error [ppm]
$C_6H_{15}N_4O_2^+$	175.1189	175.1190	0.6
$C_{15}H_{28}N_5O_3^+$	326.2187	326.2187	0.0
$C_{19}H_{36}N_7O_4^+$	426.2823	426.2823	0.0
$C_{20}H_{34}N_7O_5^+$	452.2616	452.2612	-0.9

Figure S25. XIC, MS/MS spectra and MS/MS analytical data of enzymatic hydrolysis product 583.4024 *m/z*.

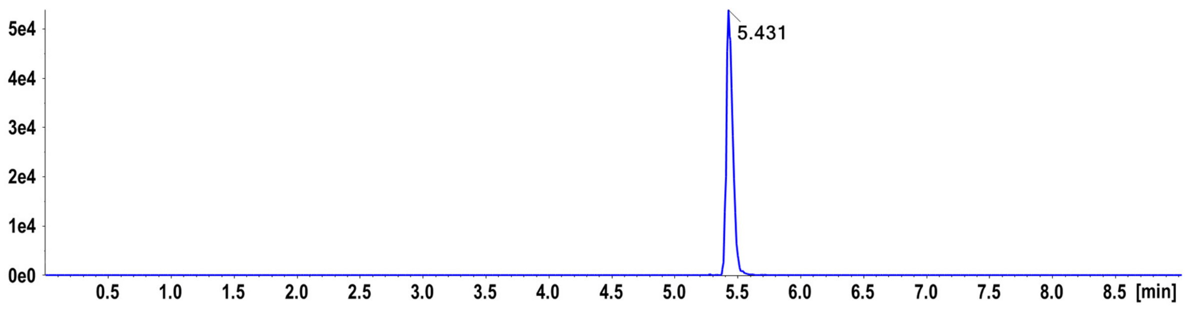

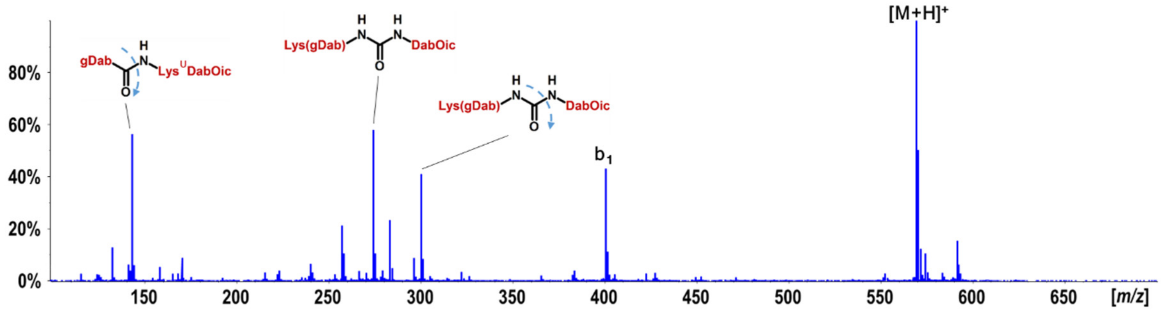

Fragment formula	*m/z* calculated	*m/z* found	Error [ppm]
$C_5H_{11}N_4O^+$	143.0927	143.0926	-0.7
$C_{11}H_{28}N_7O^+$	274.2350	274.2354	1.5
$C_{12}H_{26}N_7O_2^+$	300.2143	300.2146	1.0
$C_{16}H_{34}N_9O_3^+$	400.2779	400.2780	0.2

Figure S26. XIC, MS/MS spectra and MS/MS analytical data of enzymatic hydrolysis product 569.3880 *m/z*.

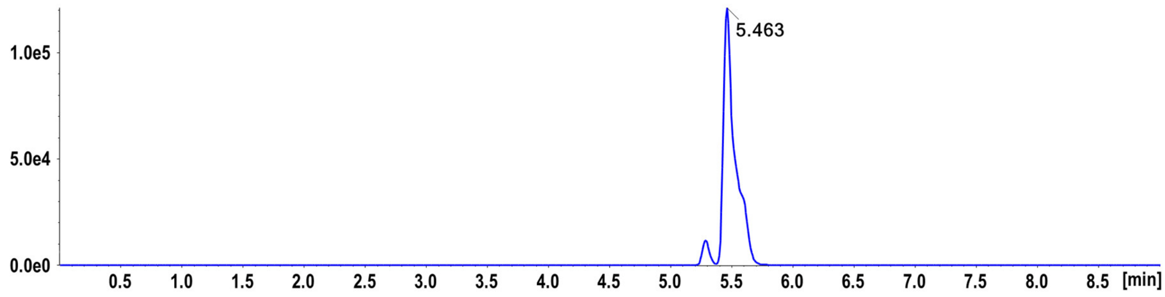

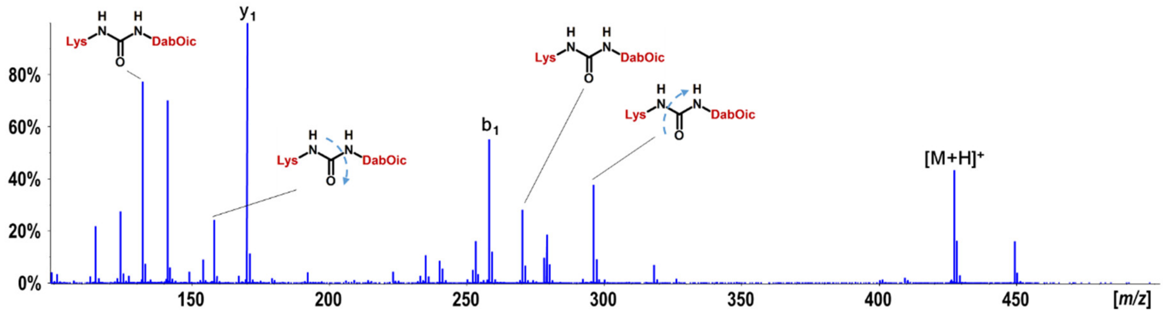

Fragment formula	*m/z* calculated	*m/z* found	Error [ppm]
$C_6H_{17}N_3^+$	132.1495	132.1490	-3.8
$C_7H_{16}N_3O^+$	158.1288	158.1286	-1.3
$C_9H_{16}NO_2^+$	170.1176	170.1175	-0.6
$C_{11}H_{24}N_5O_2^+$	258.1925	258.1927	0.8
$C_{13}H_{24}N_3O_3^+$	270.1812	270.1817	1.9
$C_{14}H_{22}N_3O_4^+$	296.1605	296.1608	1.0

Figure S27. XIC, MS/MS spectra and MS/MS analytical data of enzymatic hydrolysis product 427.3031 *m/z*.

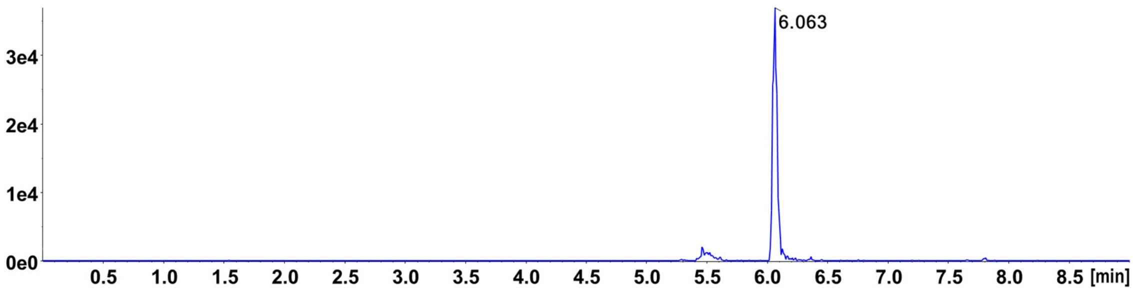

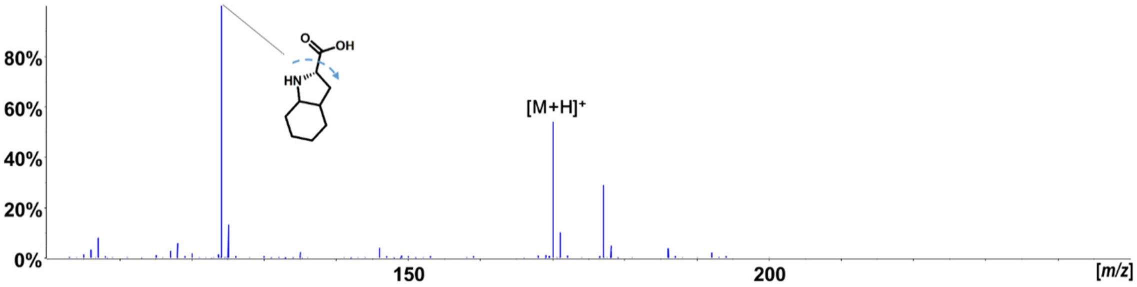

Fragment formula	*m/z* calculated	*m/z* found	Error [ppm]
$C_8H_{14}N^+$	124.1121	124.1123	1.6

Figure S28. XIC, MS/MS spectra and MS/MS analytical data of enzymatic hydrolysis product 170.1176 *m/z*.

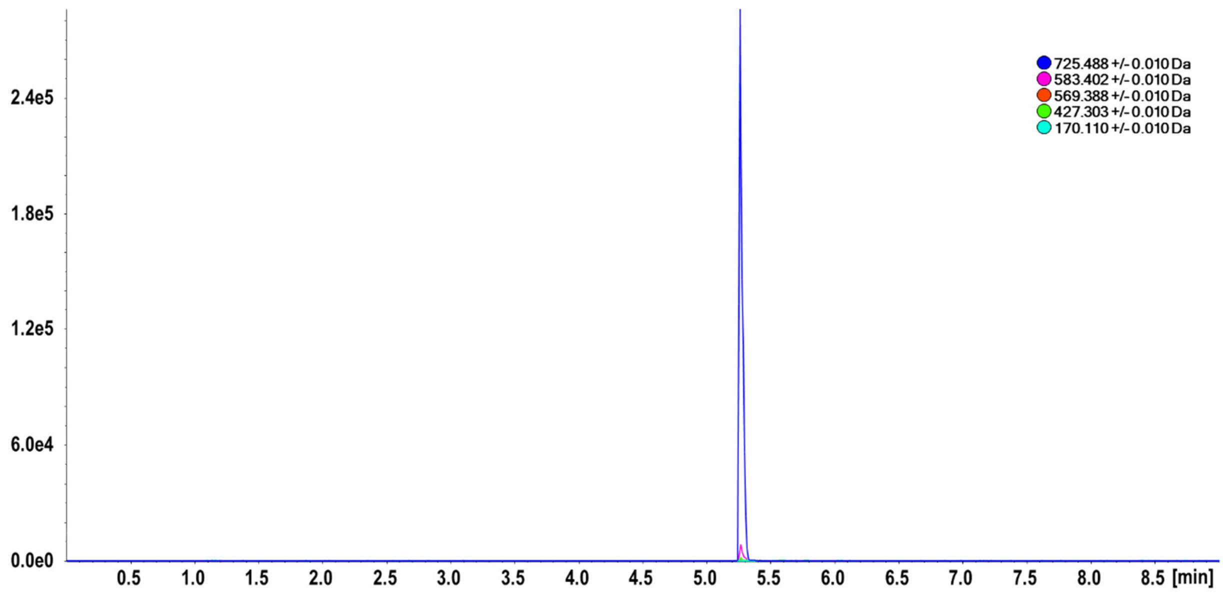

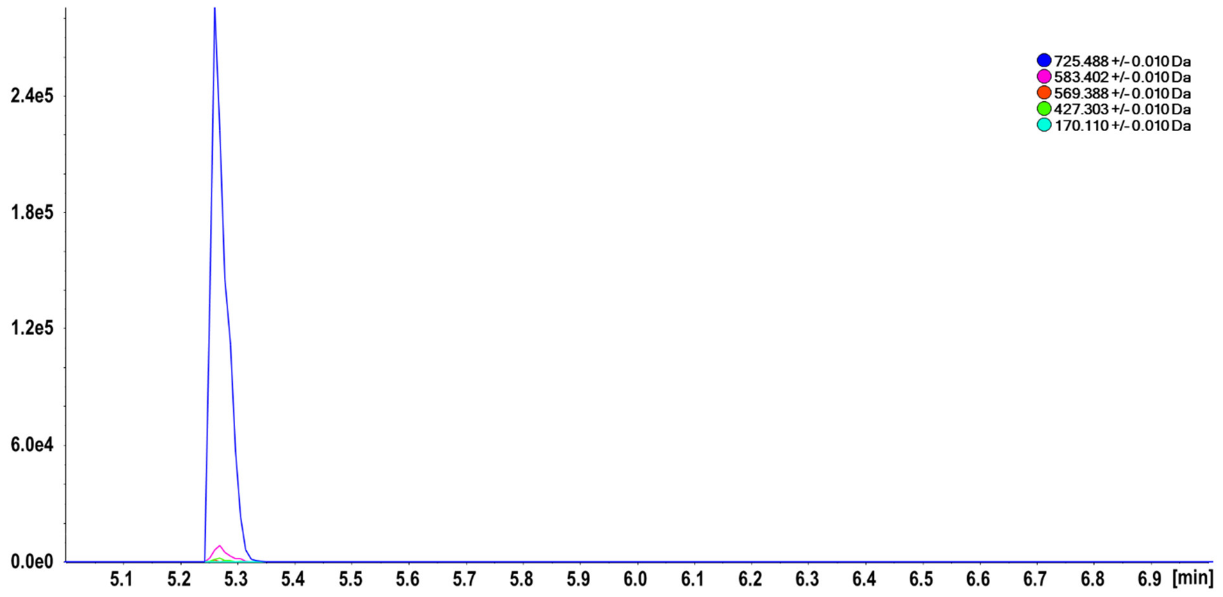

Figure S29. XICs of compound **3** and its degradation products after 0h degradation time.

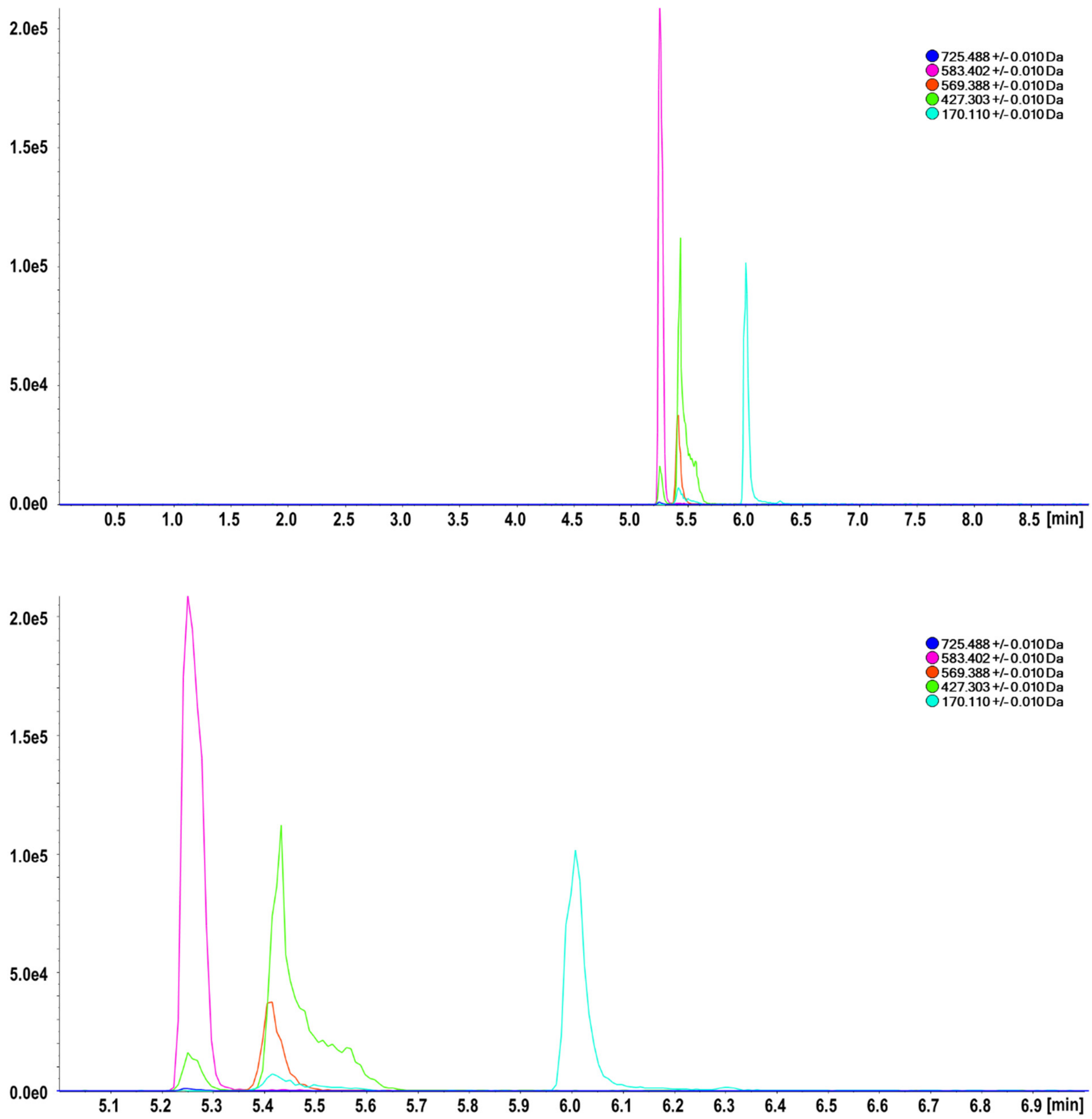

Figure S30. XICs of compound **3** and its degradation products after 96h degradation time.

Table S7. Identified products of hybrid **6** after 96h of incubation in human serum.

RT [min]	$[M+H]^+_{found}$	$[M+H]^+_{calc.}$	Error [ppm]	Formula	Predicted structure
5.26	725.4893	725.4893	0.0	$C_{52}H_{70}N_{14}O_{12}$	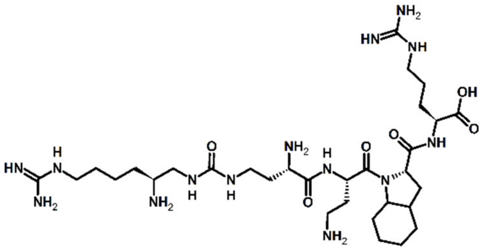
5.36/5.72	569.3886	569.3882	0.7	$C_{25}H_{49}N_{10}O_5^+$	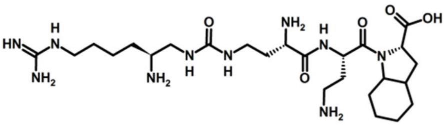
6.44/6.64	551.3783	551.3776	1.3	$C_{25}H_{46}N_{10}O_4^+$	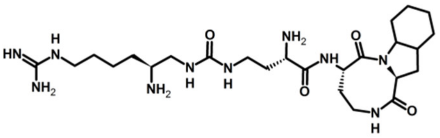
3.53	426.2821	426.2823	-0.5	$C_{19}H_{36}N_7O_4^+$	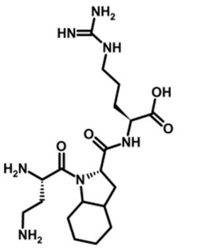
1.21	318.2255	318.2248	2.2	$C_{12}H_{27}N_7O_3^+$	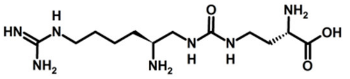
6.07	170.1170	170.1176	-3.5	$C_9H_{16}NO_2^+$	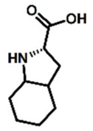

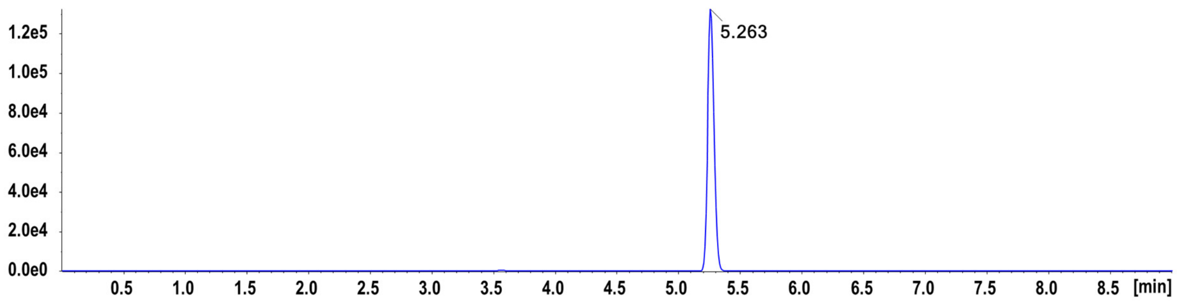

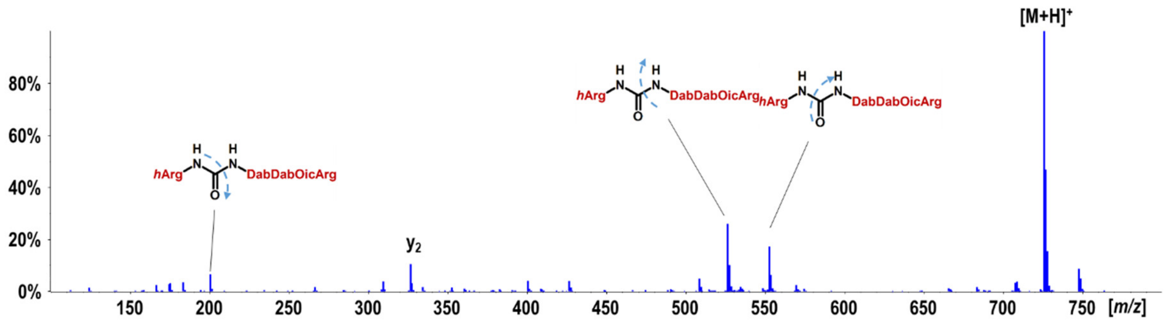

Fragment formula	m/z calculated	m/z found	Error [ppm]
$C_8H_{18}N_5O^+$	200.1506	200.1507	0.5
$C_{15}H_{28}N_5O_3^+$	326.2187	326.2194	2.1
$C_{23}H_{44}N_9O_5^+$	526.3460	526.3460	0.0
$C_{24}H_{42}N_9O_6^+$	552.3253	552.3257	0.7

Figure S31. XIC, MS/MS spectra and MS/MS analytical data of compound **6** after 96h degradation in human serum.

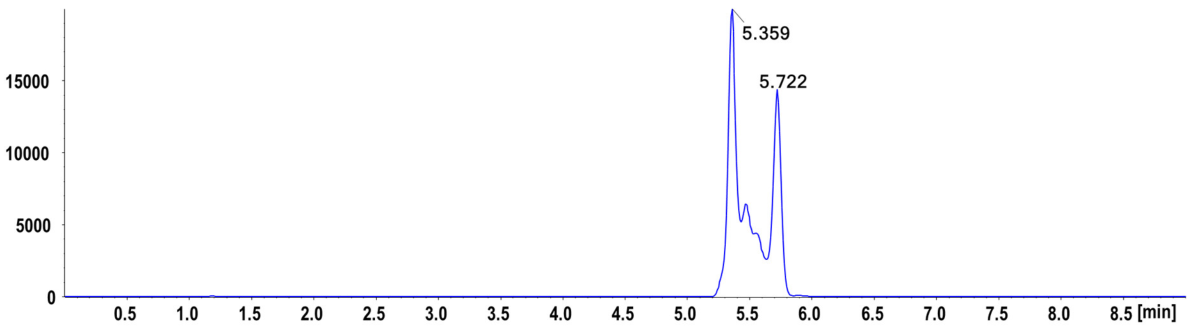

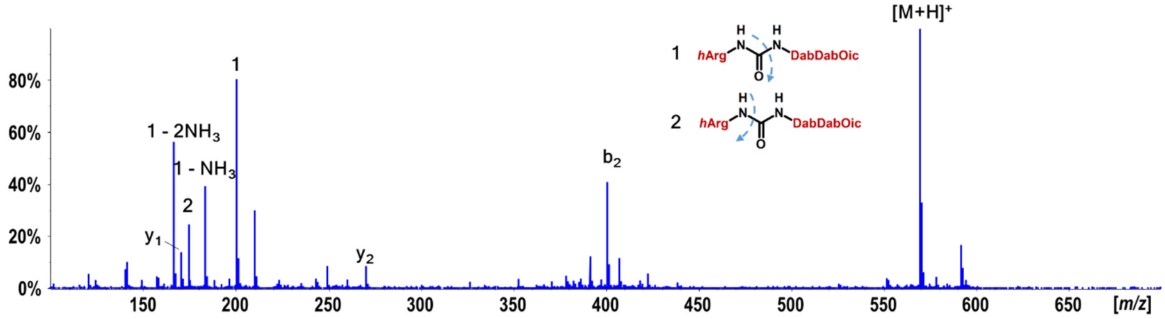

Fragment formula	*m/z* calculated	*m/z* found	Error [ppm]
$C_7H_{20}N_5^+$	174.1713	174.1717	-2.3
$C_9H_{16}NO_2^+$	170.1176	170.1171	-2.9
$C_8H_{12}N_3O^+$	166.0974	166.0971	-1.8
$C_8H_{15}N_4O^+$	183.1240	183.1233	-3.8
$C_8H_{18}N_5O^+$	200.1506	200.1504	-1.0
$C_{13}H_{24}N_3O_3$	270.1812	270.1810	-0.7
$C_{16}H_{34}N_9O_3^+$	400.2779	400.2770	-2.2

Figure S32. XIC, MS/MS spectra and MS/MS analytical data of enzymatic hydrolysis product 569.3886 *m/z*.

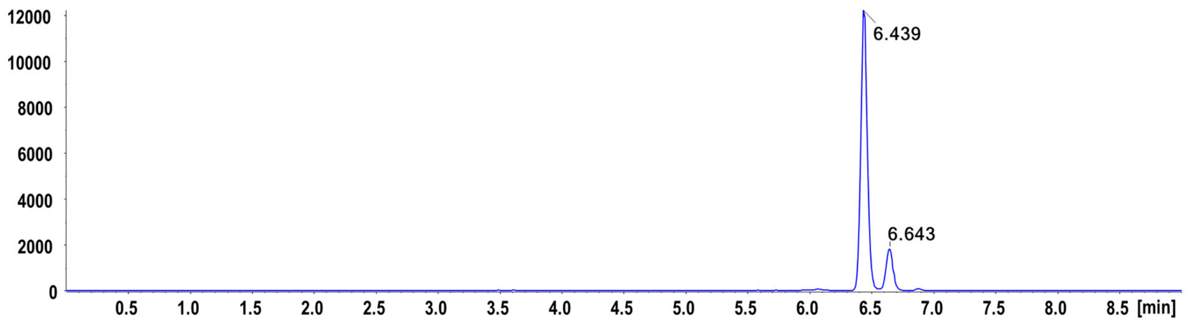

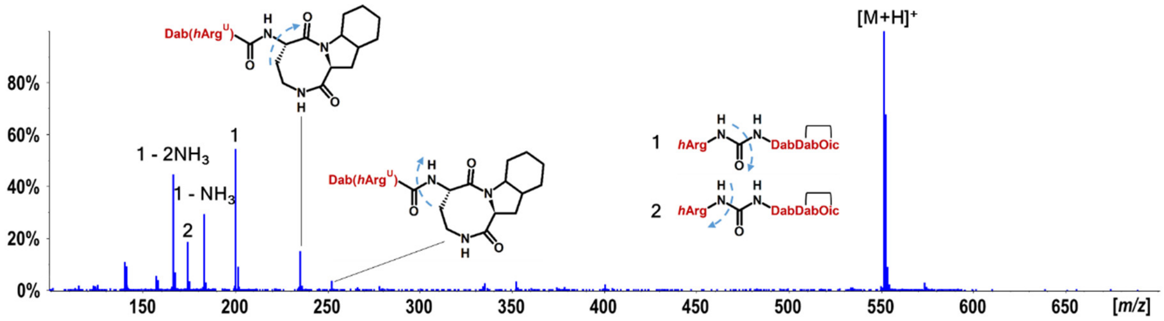

Fragment formula	*m/z* calculated	*m/z* found	Error [ppm]
$C_8H_{12}N_3O^+$	166.0774	166.0973	-0.6
$C_7H_{20}N_5^+$	174.1713	174.1712	-0.6
$C_8H_{15}N_4O^+$	183.1240	183.1240	0.0
$C_8H_{18}N_5O^+$	200.1506	200.1505	-0.5
$C_{13}H_{19}N_2O_2^+$	235.1441	235.1437	-1.7
$C_{13}H_{22}N_3O_2^+$	252.1707	252.1707	0.0

Figure S33. XIC, MS/MS spectra and MS/MS analytical data of enzymatic hydrolysis product 551.3783 *m/z*.

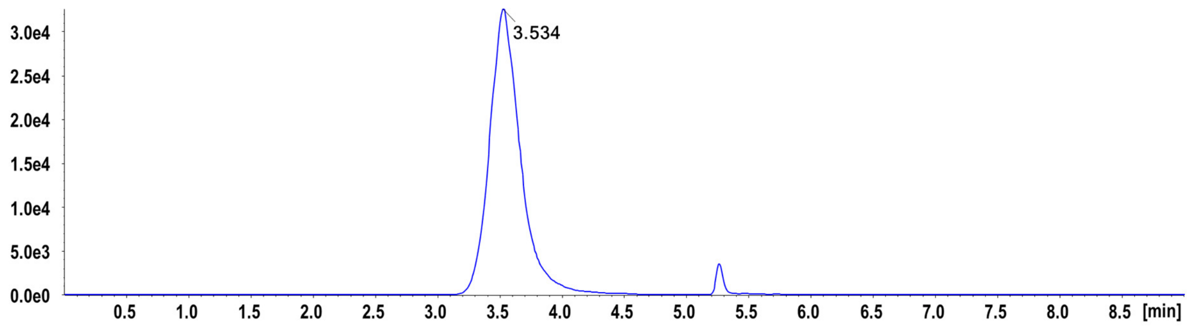

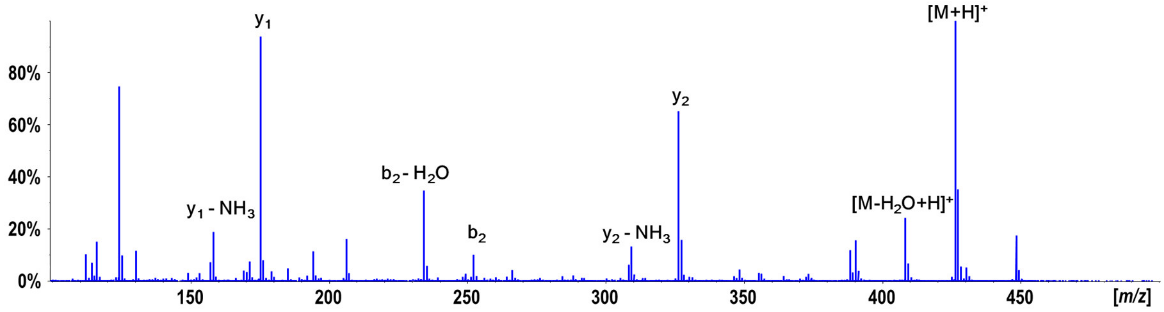

Fragment formula	*m/z* calculated	*m/z* found	Error [ppm]
$C_6H_{15}N_4O_2^+$	158.0924	158.0924	-2.5
$C_6H_{15}N_4O_2^+$	175.1190	175.1190	-0.6
$C_{13}H_{20}N_3O^+$	234.1601	234.1600	-0.4
$C_{13}H_{22}N_3O_2^+$	252.1707	252.1711	1.6
$C_{15}H_{25}N_4O_3^+$	309.1921	309.1921	2.3
$C_{15}H_{28}N_5O_3^+$	326.2187	326.2189	0.6
$C_{19}H_{34}N_7O_3^+$	408.2718	408.2716	-0.5

Figure S34. XIC, MS/MS spectra and MS/MS analytical data of enzymatic hydrolysis product 426.2823 *m/z*.

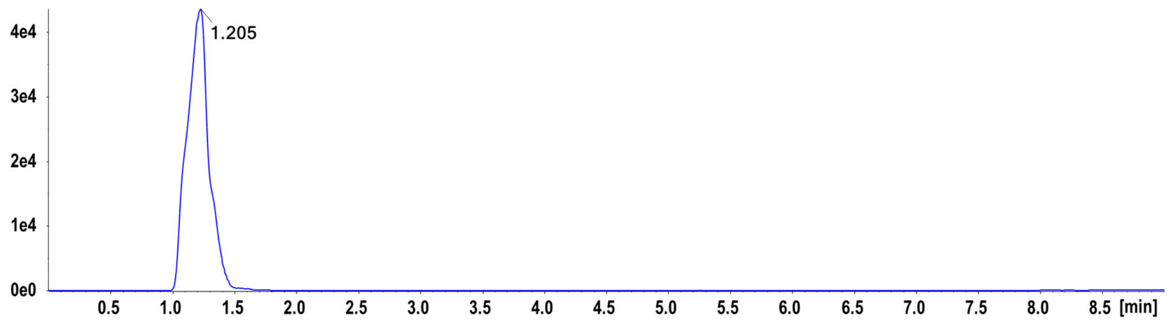

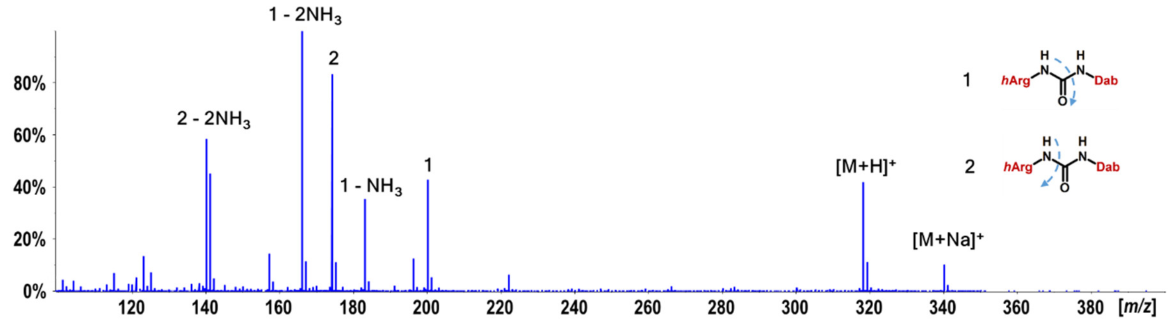

Fragment formula	*m/z* calculated	*m/z* found	Error [ppm]
$C_7H_{14}N_3^+$	140.1182	140.1178	-2.9
$C_7H_{20}N_5^+$	174.1713	174.1714	0.6
$C_8H_{12}N_3O^+$	166.0974	166.0977	1.8
$C_8H_{15}N_4O^+$	183.1240	183.1236	-2.2
$C_8H_{18}N_5O^+$	200.1506	200.1507	0.5

Figure S35. XIC, MS/MS spectra and MS/MS analytical data of enzymatic hydrolysis product 318.2255 *m/z*.

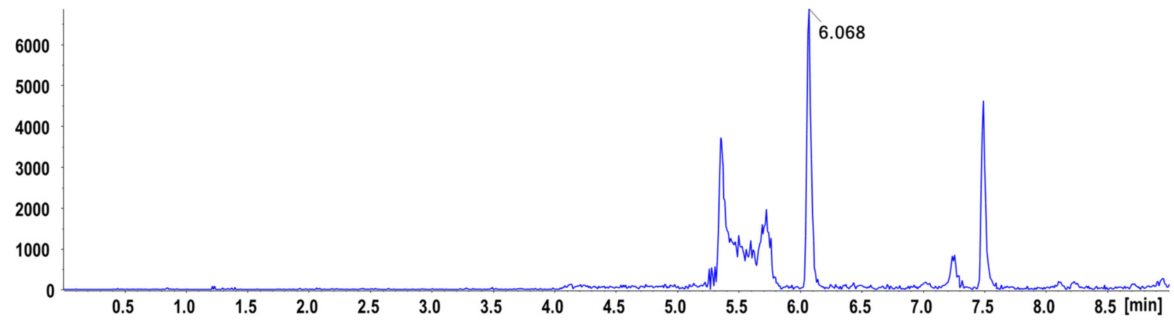

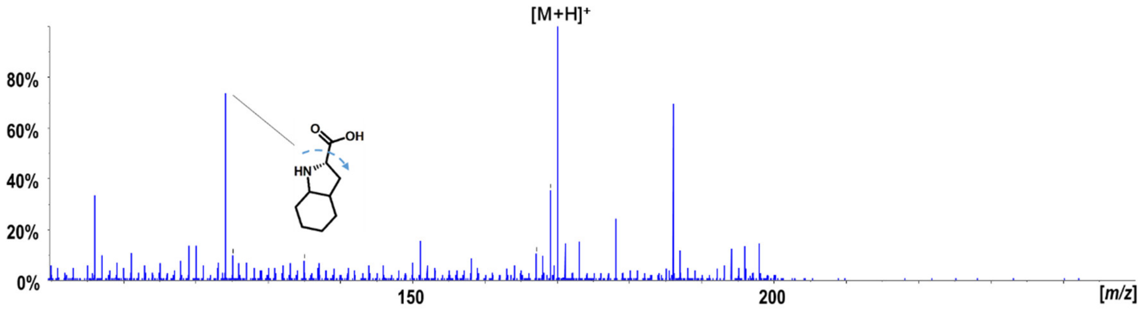

Fragment formula	*m/z* calculated	*m/z* found	Error [ppm]
$C_8H_{14}N^+$	124.1121	124.1123	1.6

Figure S36. XIC, MS/MS spectra and MS/MS analytical data of enzymatic hydrolysis product 170.1176 *m/z*.

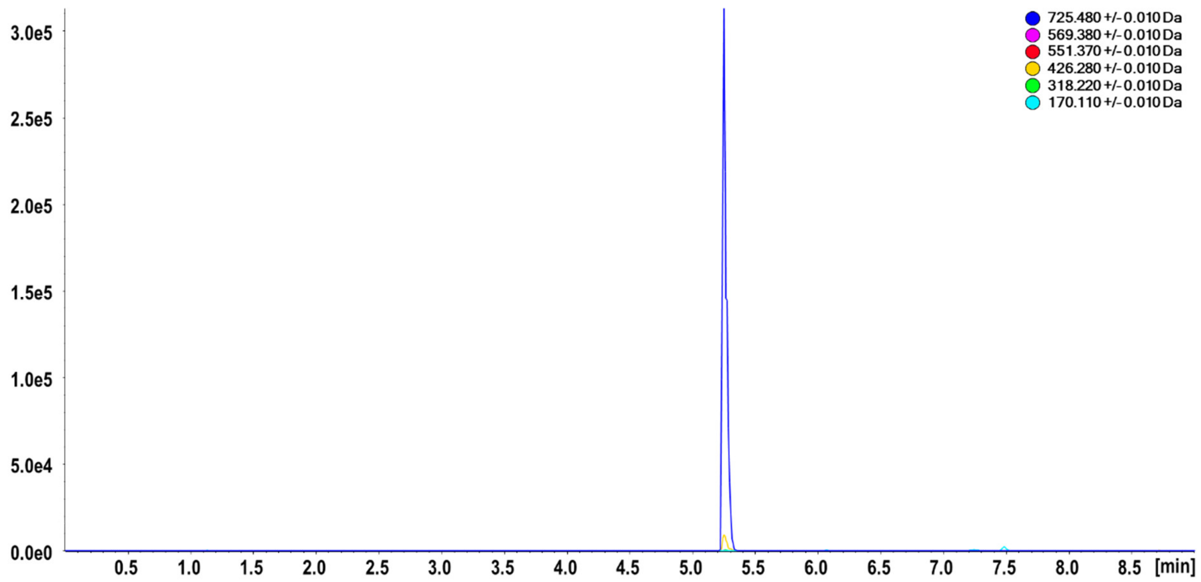

Figure S37. XICs of compound **6** and its degradation products after 0h degradation time.

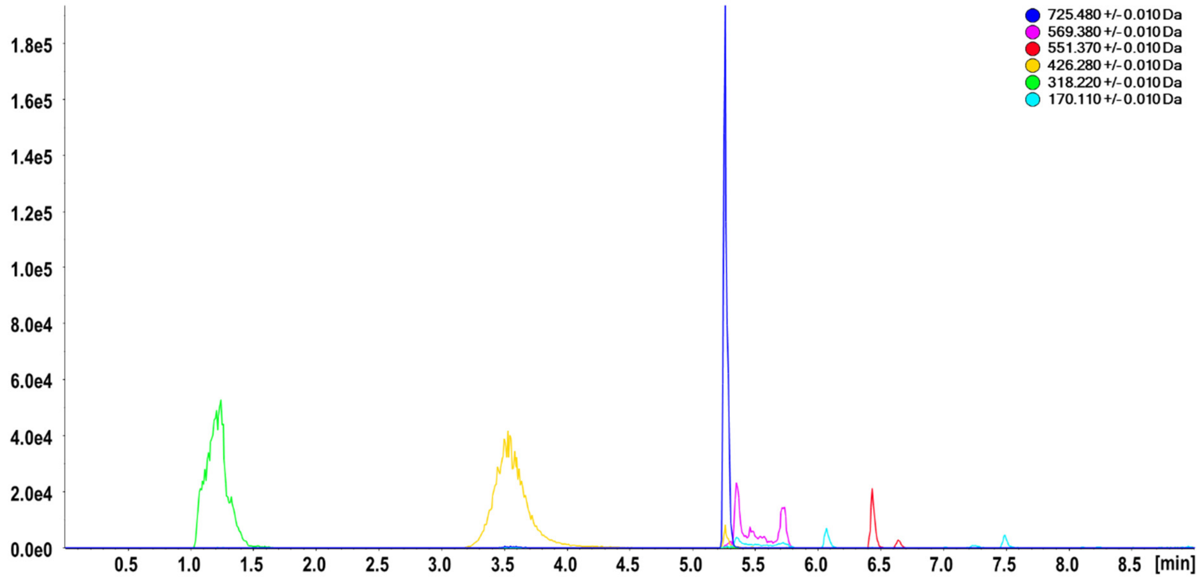

Figure S38. XICs of compound **6** and its degradation products after 96h degradation time.

Table S8. Identified products of hybrid **7** after 96h of incubation in human serum.

RT [min]	$[M+H]^+_{found}$	$[M+H]^+_{calc.}$	Error [ppm]	Formula	Predicted structure
5.25	726.4811	726.4846	-4.8	$C_{30}H_{59}N_{15}O_6^+$	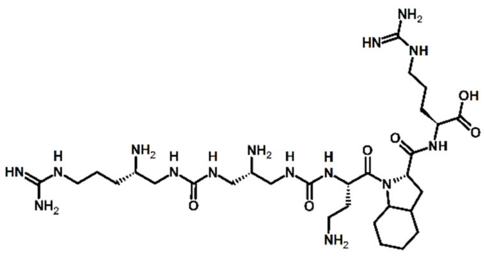
5.35	570.3834	570.3821	-2.3	$C_{24}H_{48}N_{11}O_5^+$	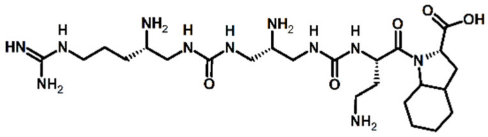
1.20	401.2732	401.2741	-2.2	$C_{15}H_{33}N_{10}O_3^+$	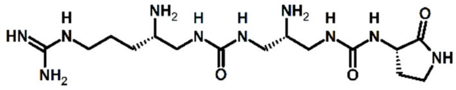
6.07	170.1174	170.1176	-1.2	$C_9H_{16}NO_2^+$	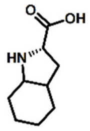

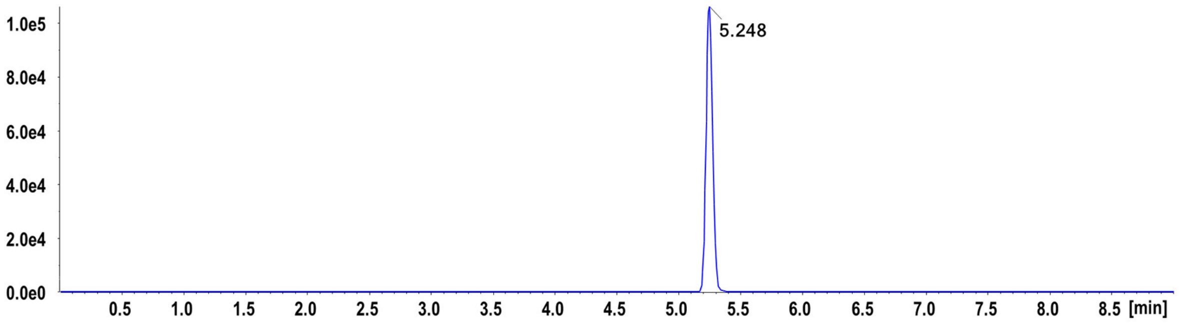

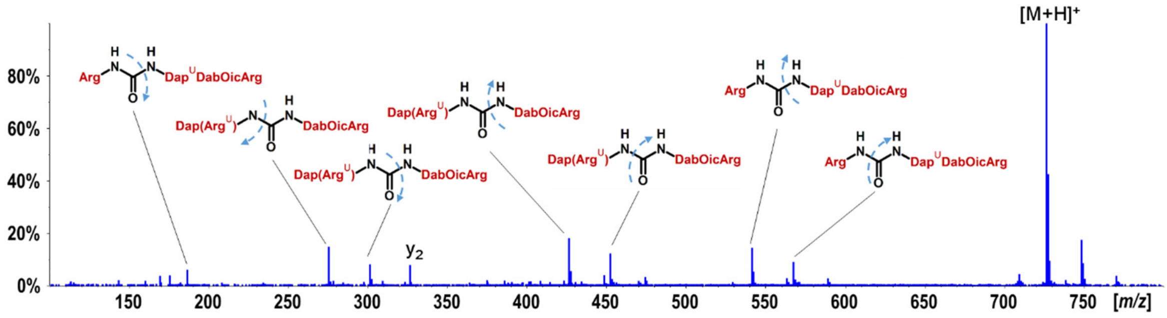

Fragment formula	*m/z* calculated	*m/z* found	Error [ppm]
$C_7H_{16}N_5O^+$	186.1349	186.1350	0.5
$C_{10}H_{27}N_8O^+$	275.2302	275.2295	-2.5
$C_{11}H_{25}N_8O_2^+$	301.2095	301.2092	-1.0
$C_{15}H_{28}N_5O_3^+$	326.2187	326.2172	-4.6
$C_{19}H_{36}N_7O_4^+$	426.2823	426.2809	-3.3
$C_{20}H_{34}N_7O_5^+$	452.2616	452.2609	-1.5
$C_{23}H_{45}N_{10}O_5^+$	541.3569	541.3554	-2.8
$C_{24}H_{43}N_{10}O_6^+$	567.3362	567.3342	-3.5

Figure S39. XIC, MS/MS spectra and MS/MS analytical data of compound **7** after 96h degradation in human serum.

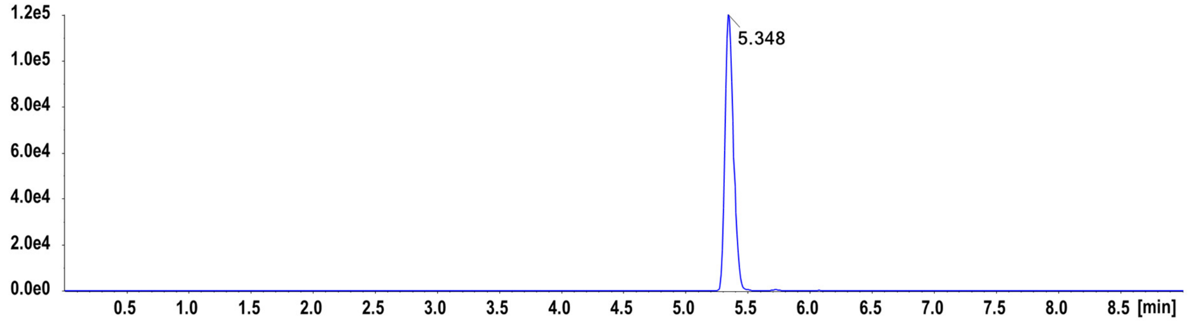

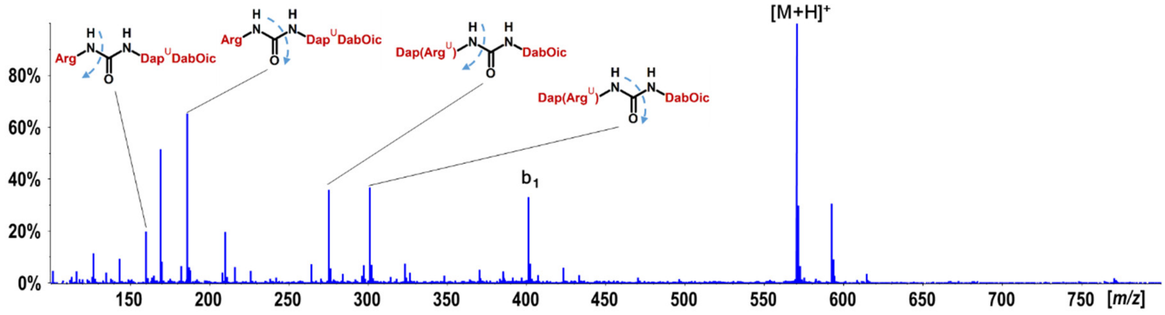

Fragment formula	*m/z* calculated	*m/z* found	Error [ppm]
$C_6H_{18}N_5^+$	160.1557	160.1548	-5.6
$C_7H_{16}N_5O^+$	186.1349	186.1346	-1.6
$C_{10}H_{27}N_8O^+$	275.2302	275.2297	-1.8
$C_{11}H_{25}N_8O_2^+$	301.2095	301.2092	-1.0
$C_{15}H_{33}N_{10}O_3^+$	401.2732	401.2732	0.0

Figure S40. XIC, MS/MS spectra and MS/MS analytical data of enzymatic hydrolysis product 570.3834 *m/z*.

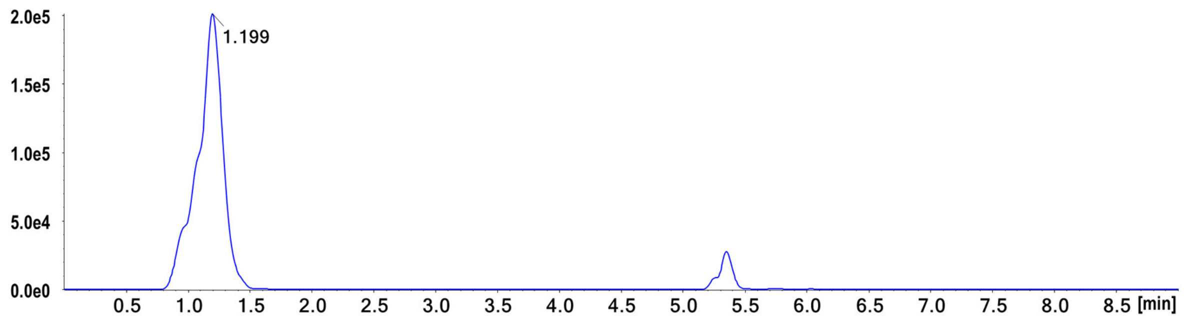

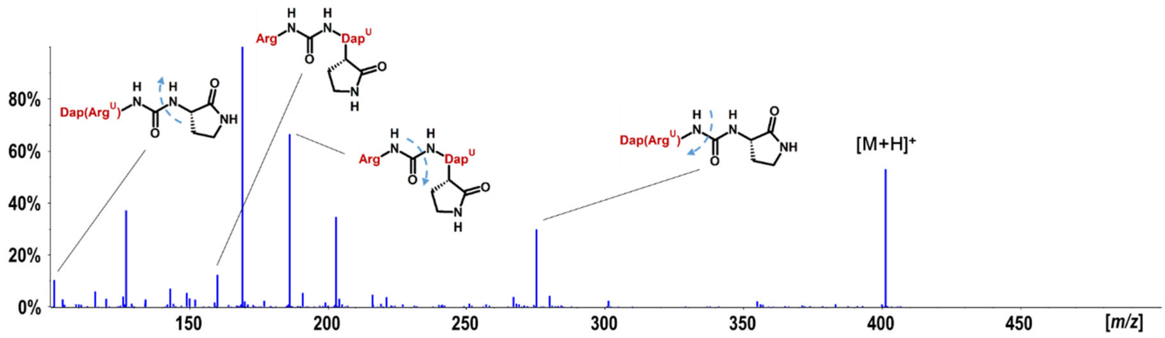

Fragment formula	*m/z* calculated	*m/z* found	Error [ppm]
$C_4H_9N_2O^+$	101.0709	101.0706	-3.0
$C_7H_{16}N_5O^+$	160.1557	160.1558	0.6
$C_8H_{12}N_3O^+$	186.1349	186.1356	3.8
$C_{10}H_{27}N_8O^+$	275.2302	275.2314	4.4

Figure S41. XIC, MS/MS spectra and MS/MS analytical data of enzymatic hydrolysis product 401.2732 *m/z*.

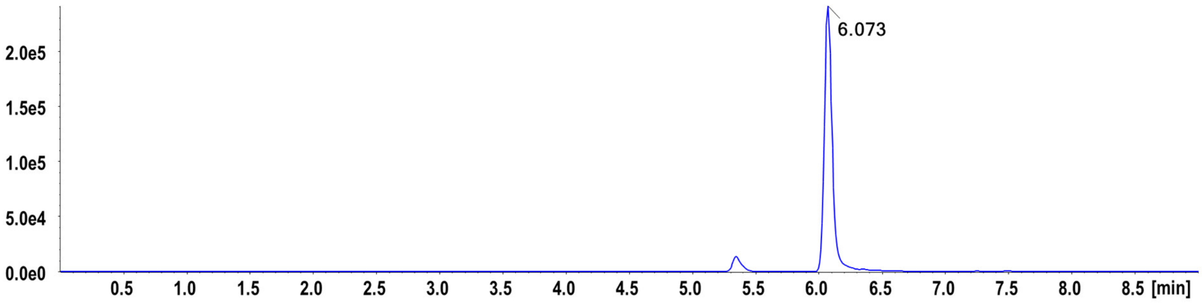

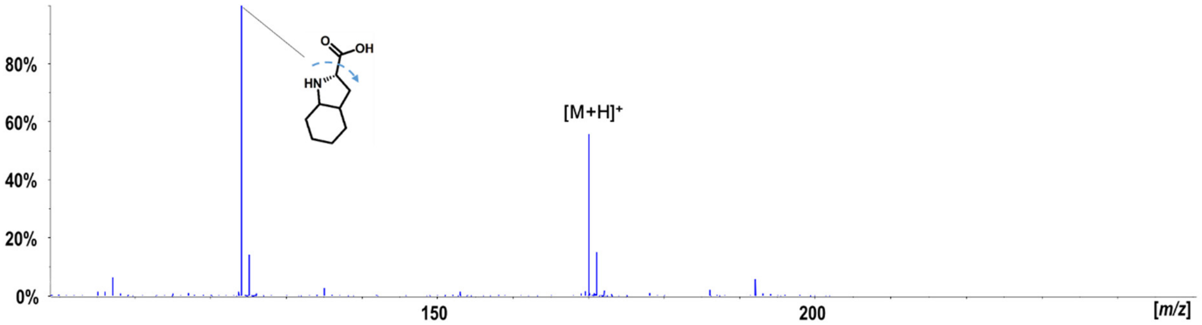

Fragment formula	*m/z* calculated	*m/z* found	Error [ppm]
$C_8H_{14}N^+$	124.1121	124.1119	-1.6

Figure S42. XIC, MS/MS spectra and MS/MS analytical data of enzymatic hydrolysis product 170.1176 *m/z*.

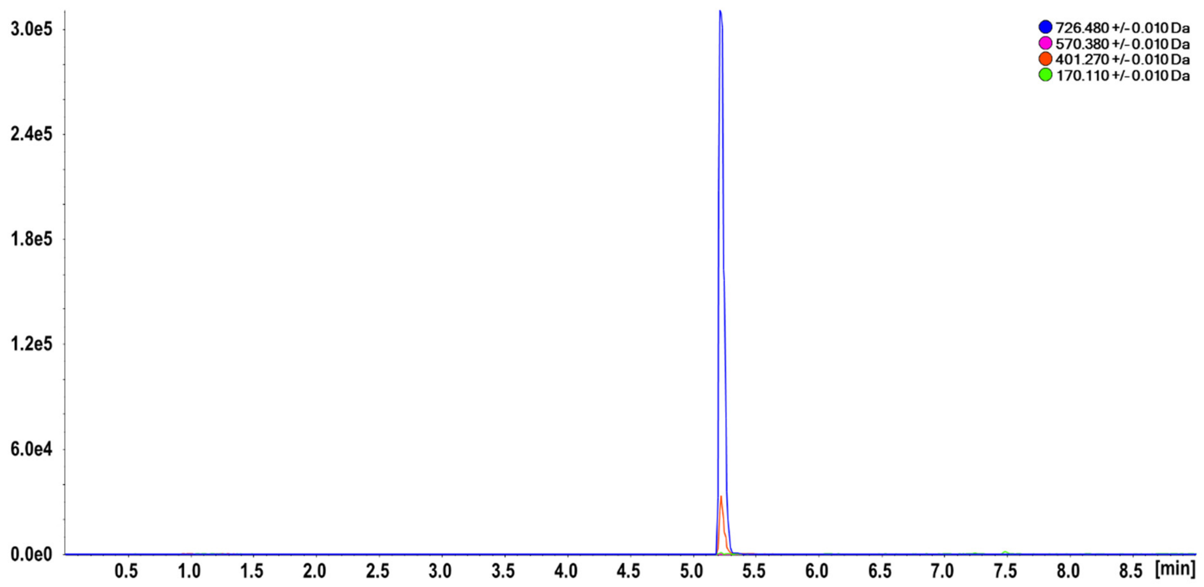

Figure S43. XICs of compound **7** and its degradation products after 0h degradation time.

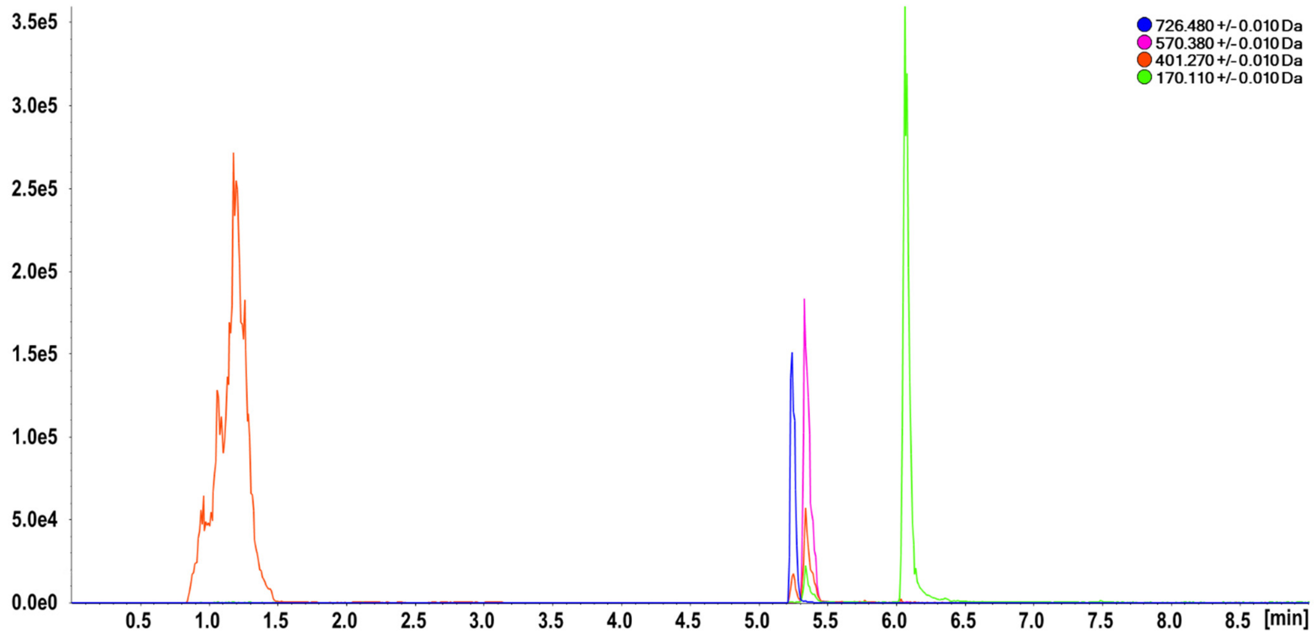

Figure S44. XICs of compound **7** and its degradation products after 96h degradation time.

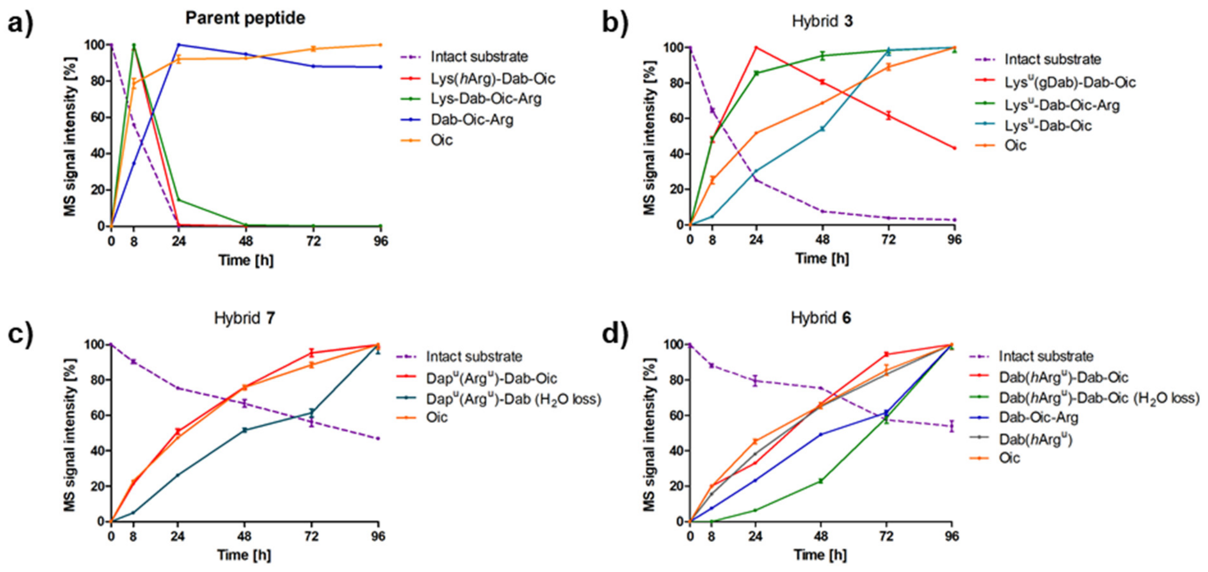

Figure S45. Level of found metabolites at different time intervals (represented as percentage of highest peak area) for **a)** parent peptide, **b)** hybrid **3**, **c)** hybrid **6** and **d)** hybrid **7**. All results are represented as an average from 3 individual experiments on LCMS with error bars indicating ± SEM (N = 3).

8. 2D NMR characterisation of parent peptide H_2N-Lys(*h*Arg)-Dab-Oic-Arg-OH.

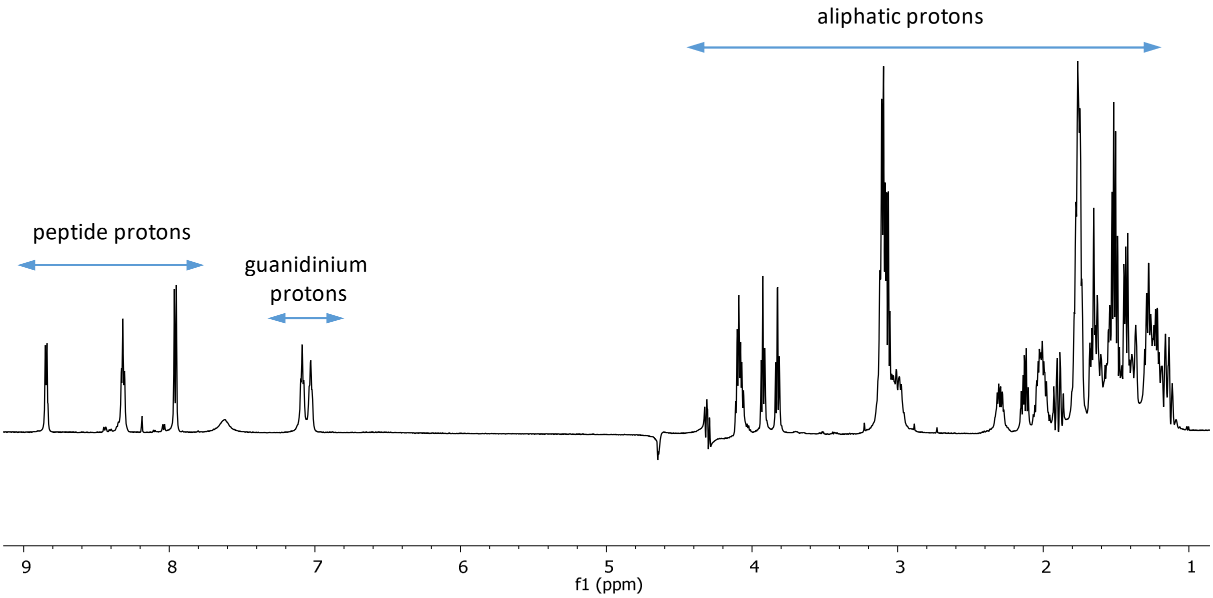

Figure S46. ^{1}H NMR spectrum of parent peptide **H_2N-Lys(*h*Arg)-Dab-Oic-Arg-OH** (600MHz, 9:1 DPBS buffer:D_2O).

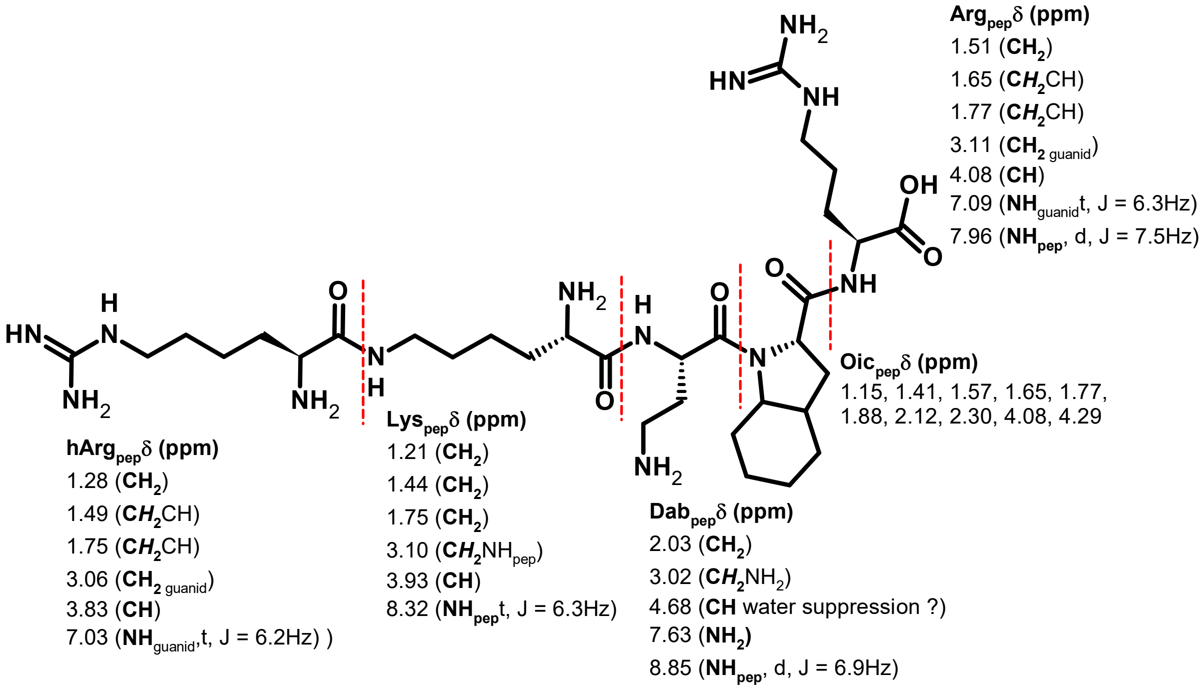

Figure S47. Assignment of signals in parent peptide **H_2N-Lys(*h*Arg)-Dab-Oic-Arg-OH** based on the correlations found in 2D NMR spectra (COSY, TOCSY, HSQC).

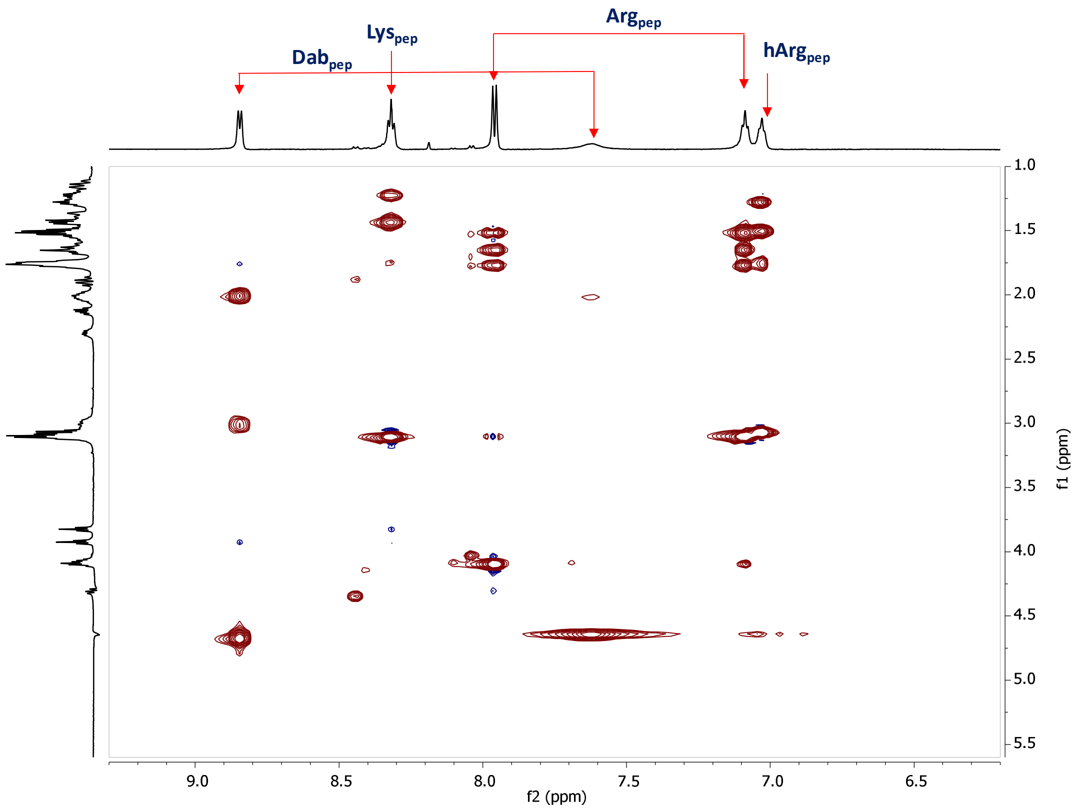

Figure S48. The fingerprint of NH_{pep}/aliphatic protons region of TOCSY spectrum of parent peptide **H_2N-Lys(*h*Arg)-Dab-Oic-Arg-OH**, recorded in 9:1 DPBS buffer:D_2O (600MHz).

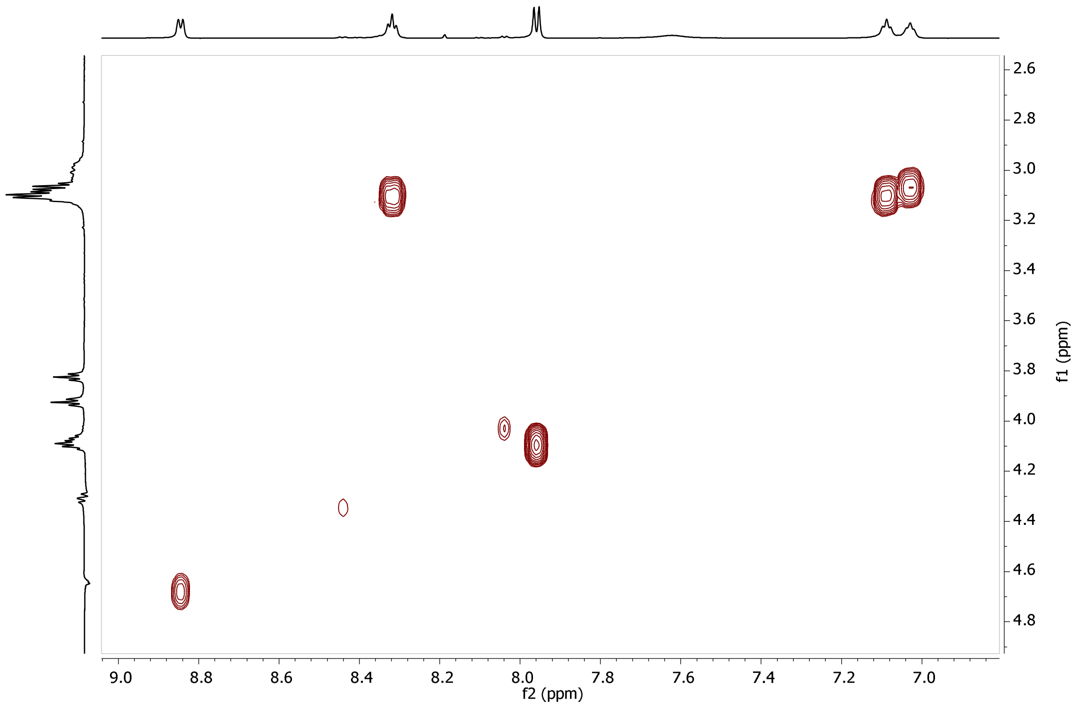

Figure S49. The fingerprint of NH_{pep}/CH, CH_2 region of COSY spectrum of parent peptide **H_2N-Lys(*h*Arg)-Dab-Oic-Arg-OH**, recorded in 9:1 DPBS buffer:D_2O (600MHz).

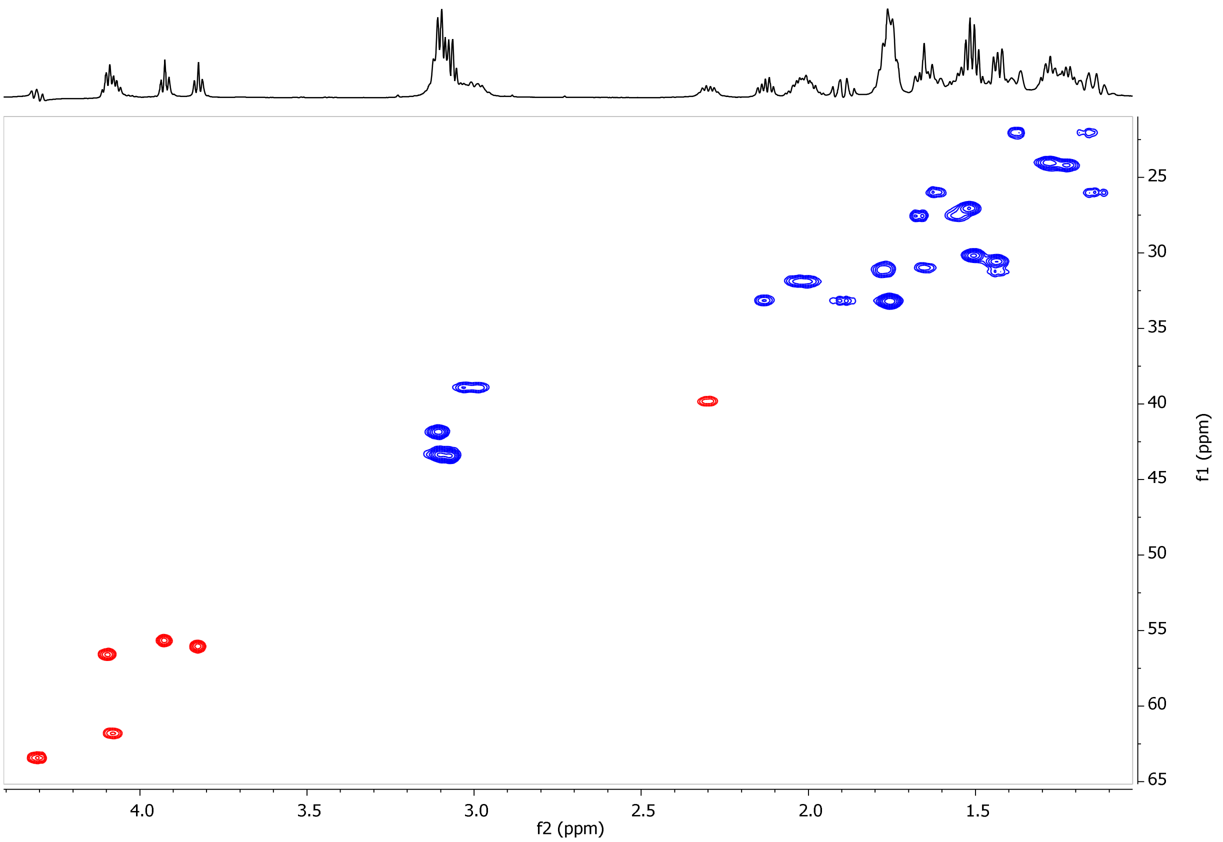

Figure S50. HSQC (13C/1H correlations) spectrum of parent peptide **H_2N-Lys(*h*Arg)-Dab-Oic-Arg-OH**, recorded in 9:1 DPBS buffer:D_2O (600MHz). CH groups marked red, while CH_2 groups marked blue.

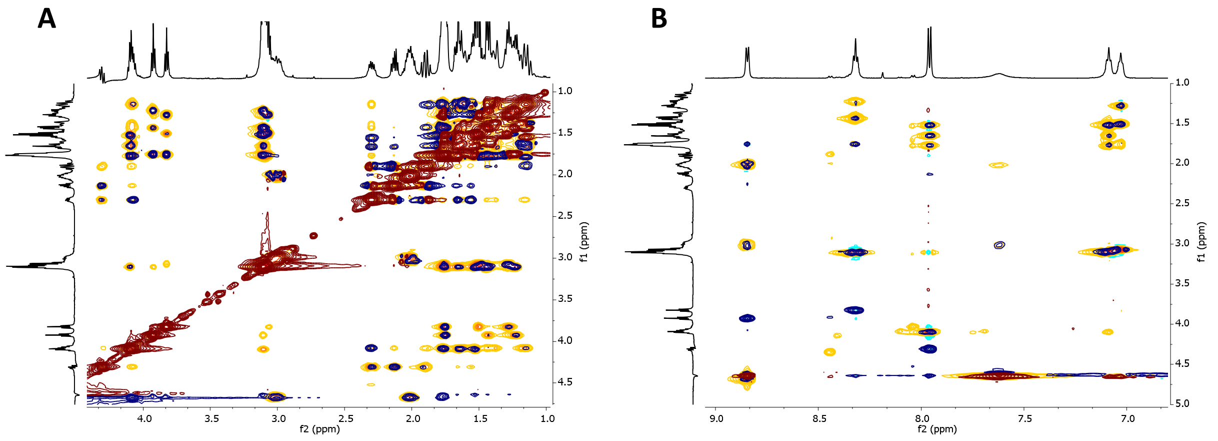

Figure S51. The imposition of TOCSY (yellow/cyan colours) and ROESY (blue/maroon colours) spectra of parent peptide **H_2N-Lys(hArg)-Dab-Oic-Arg-OH A)** aliphatic region; **B)** NH_{pep}/aliphatic region, recorded in 9:1 DPBS buffer: D_2O (600MHz)

9. 2D NMR characterisation of hybrid 6.

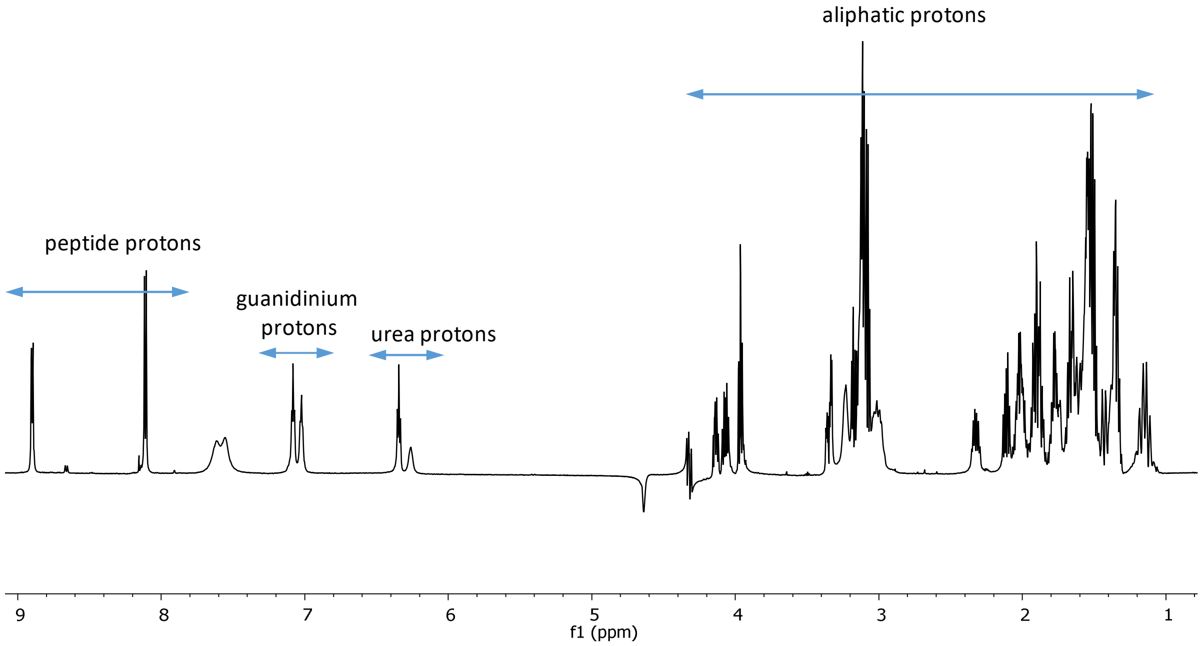

Figure S52. ^{1}H NMR spectrum of hybrid **6** (600MHz, 9:1 DPBS buffer:D_2O).

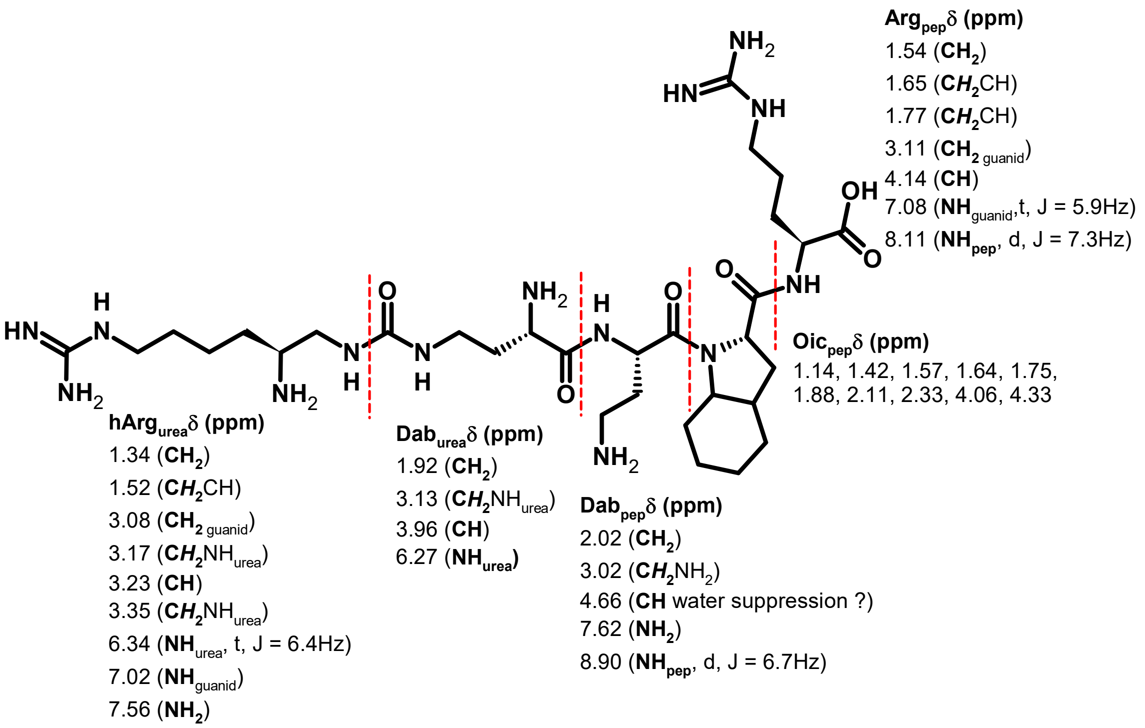

Figure S53. Assignments of signals in hybrid **6** based on the correlations found in 2D NMR spectra (COSY, TOCSY, HSQC).

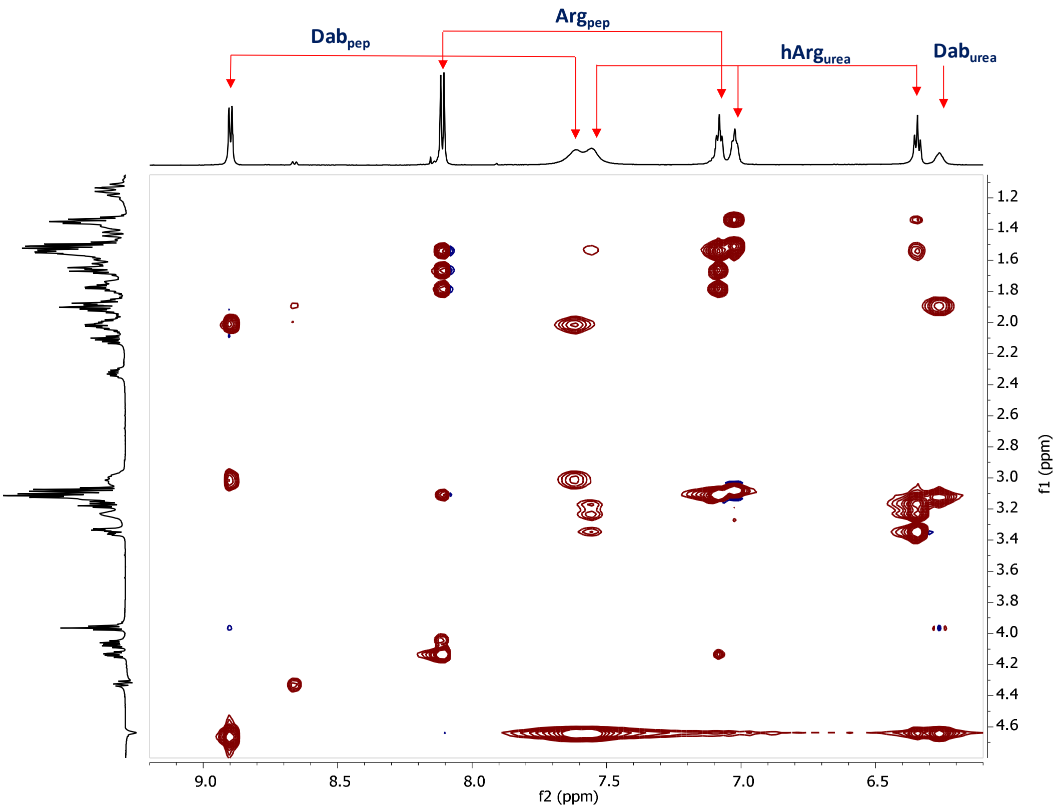

Figure S54. The fingerprint of NH_{pep}/aliphatic protons and NH_{urea}/aliphatic protons region of TOCSY spectrum of hybrid **6**, recorded in 9:1 DPBS buffer: D_2O (600MHz)**.**

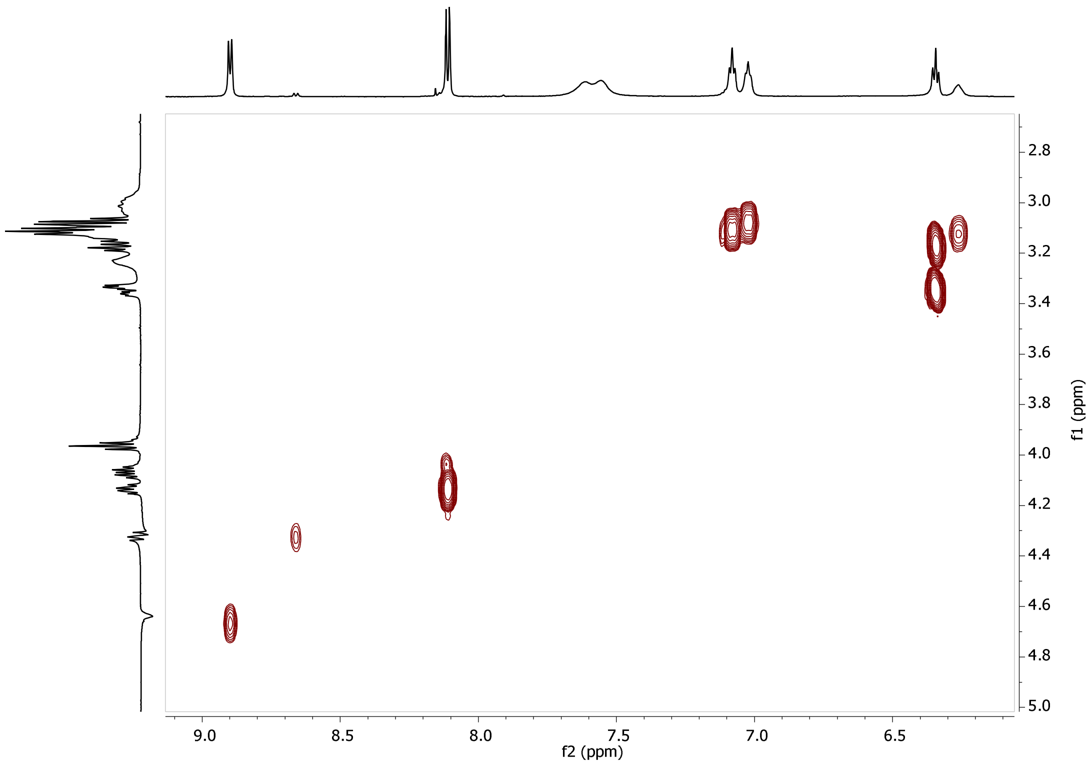

Figure S55. The fingerprint of NH_{pep}/CH and NH_{urea}/CH_2 region of COSY spectrum of hybrid **6**, recorded in 9:1 DPBS buffer: D_2O (600MHz)**.**

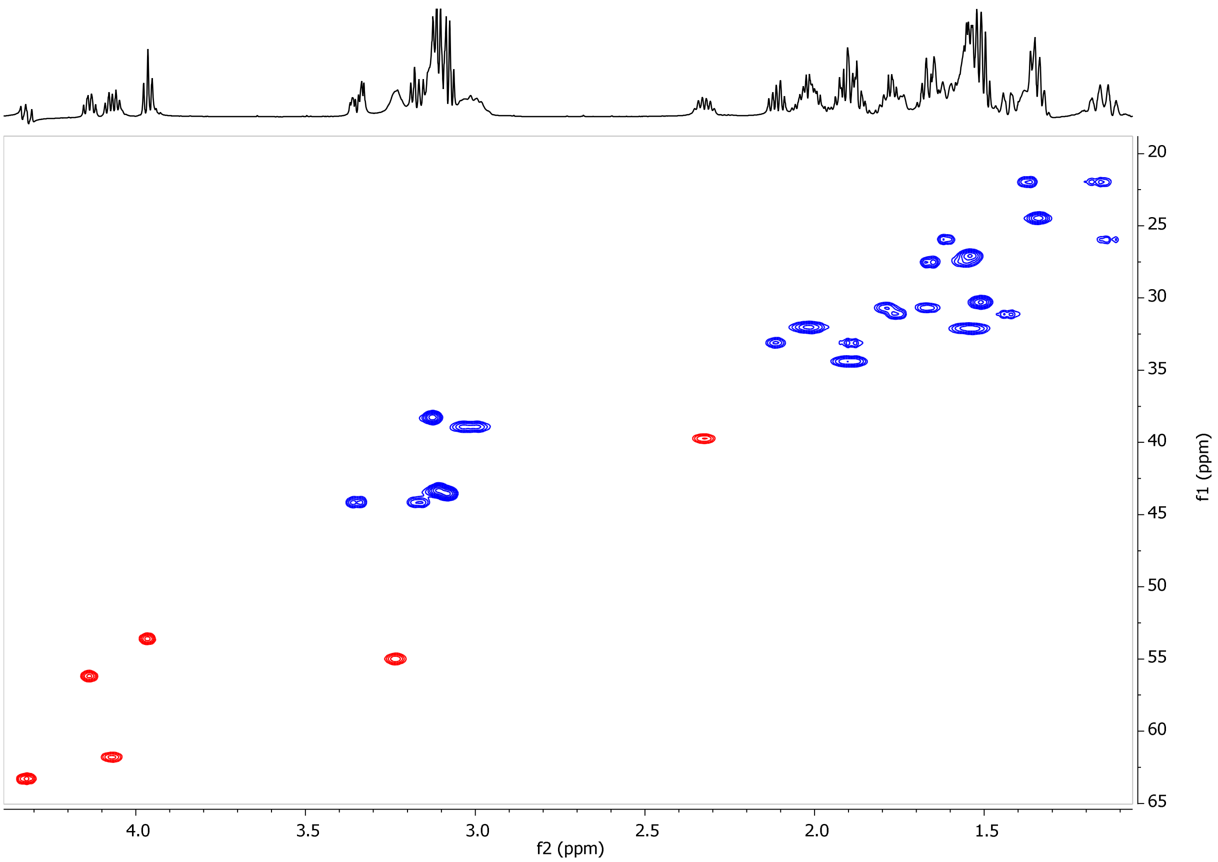

Figure S56. HSQC (13C/1H correlations) spectrum of hybrid **6**, recorded in 9:1 DPBS buffer: D_2O (600MHz). CH groups marked red, while CH_2 groups marked blue.

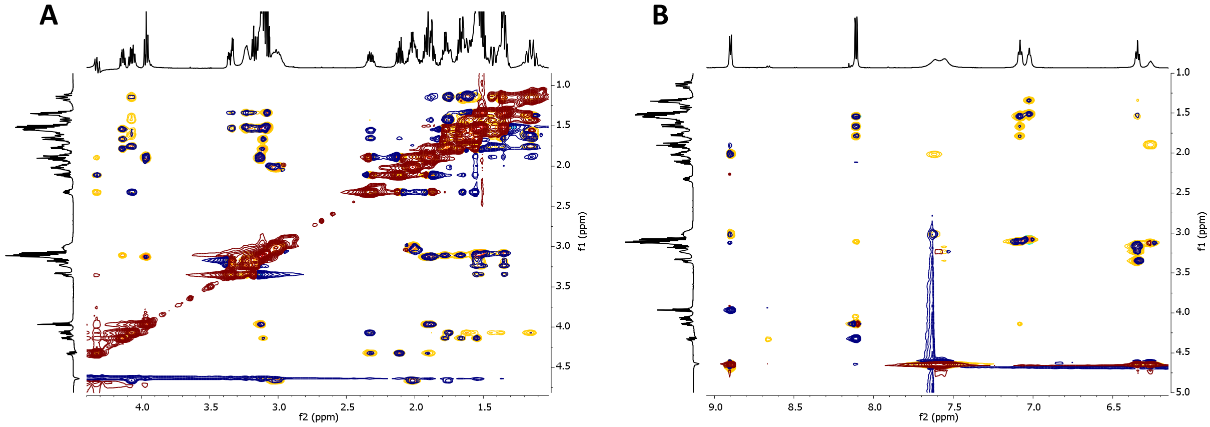

Figure S57. The imposition of TOCSY (yellow/cyan colours) and ROESY (blue/maroon colours) spectra of hybrid **6 A)** aliphatic region; **B)** $NH_{pep/urea}$/aliphatic region, recorded in 9:1 DPBS buffer: D_2O (600MHz)

10. Molecular dynamics.

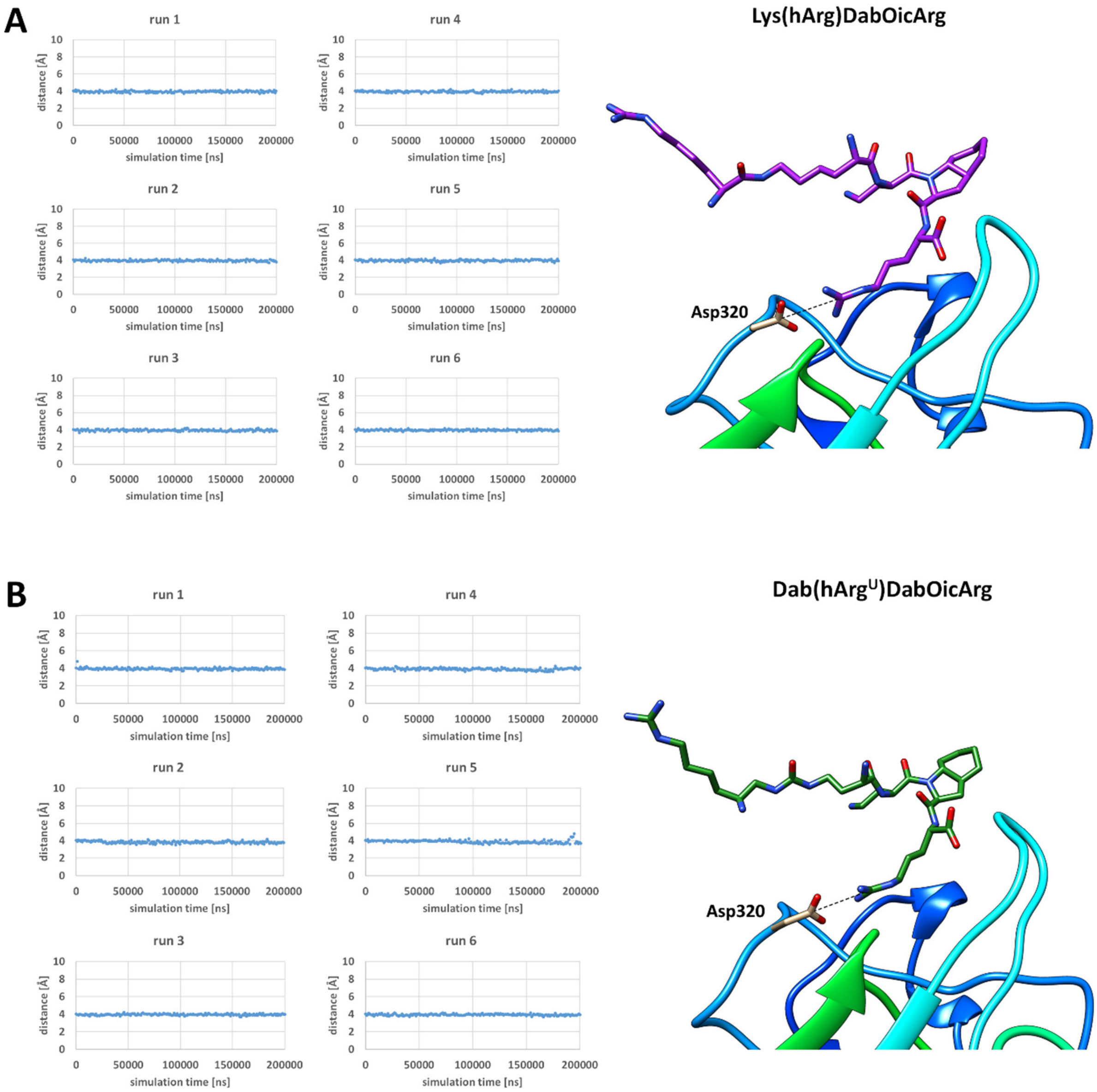

Figure S58. Time evolution of distances between Cγ of Asp320 and Cζ of Arg residue in **A)** parent peptide and **B)** compound **6**.

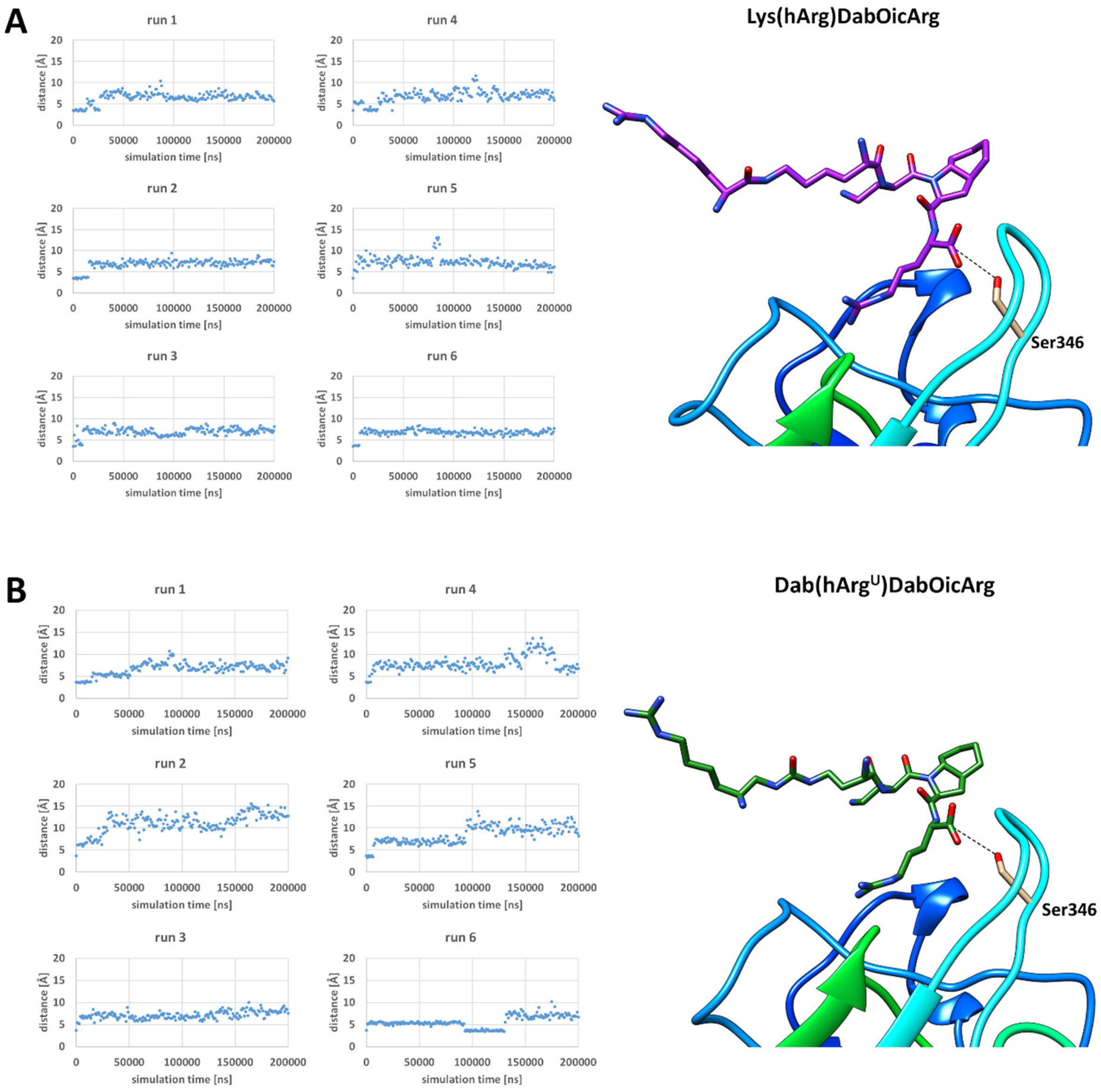

Figure S59. Time evolution of distances between Oγ of Ser346 and CO of Arg residue in **A)** parent peptide and **B)** compound **6**.

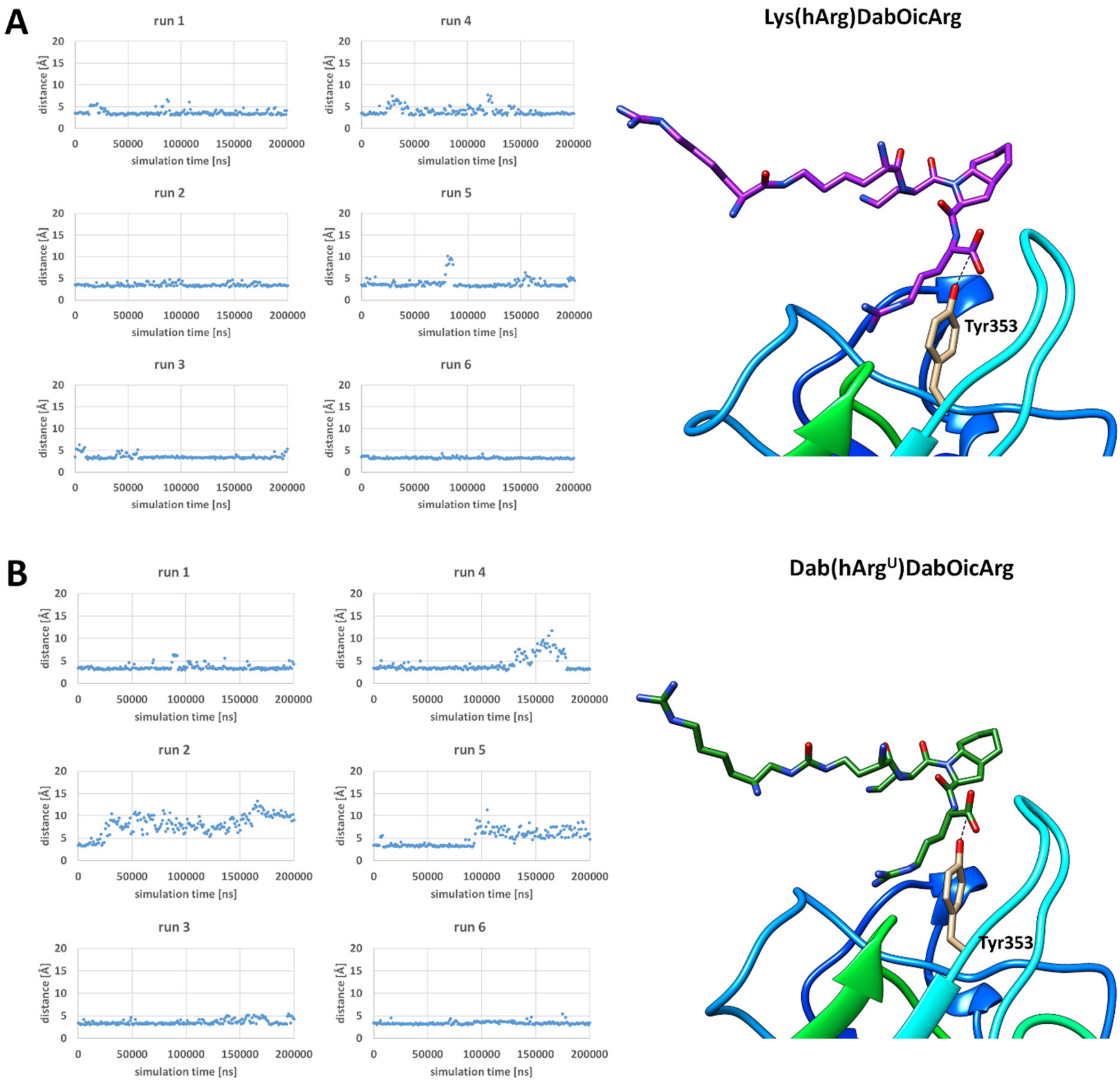

Figure S60. Time evolution of distances between Oη of Tyr353and CO of Arg residue in **A)** parent peptide and **B)** compound **6**.

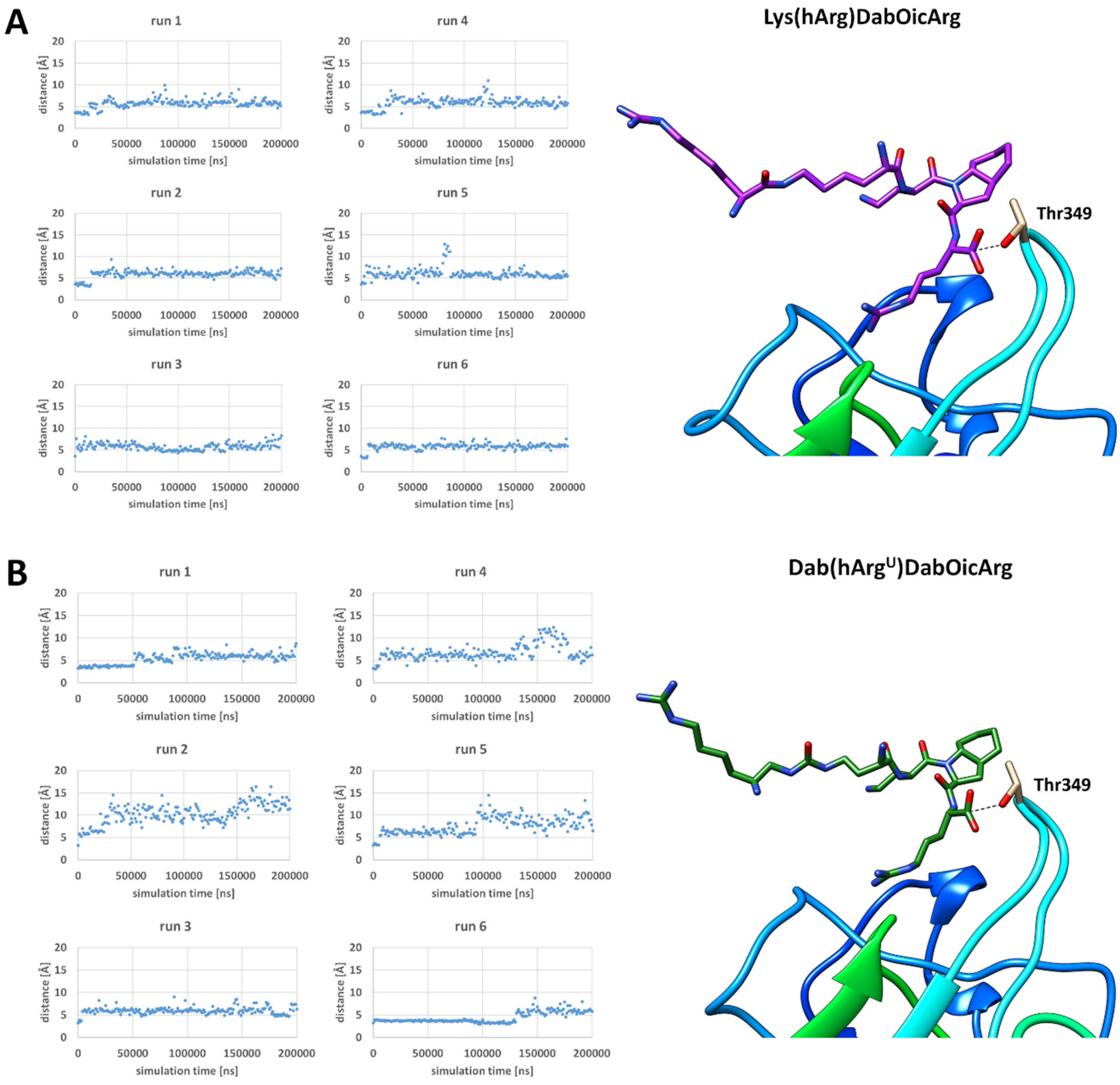

Figure S61. Time evolution of distances between Oγ of Thr349 and CO of Arg residue in **A)** parent peptide and **B)** compound **6**.

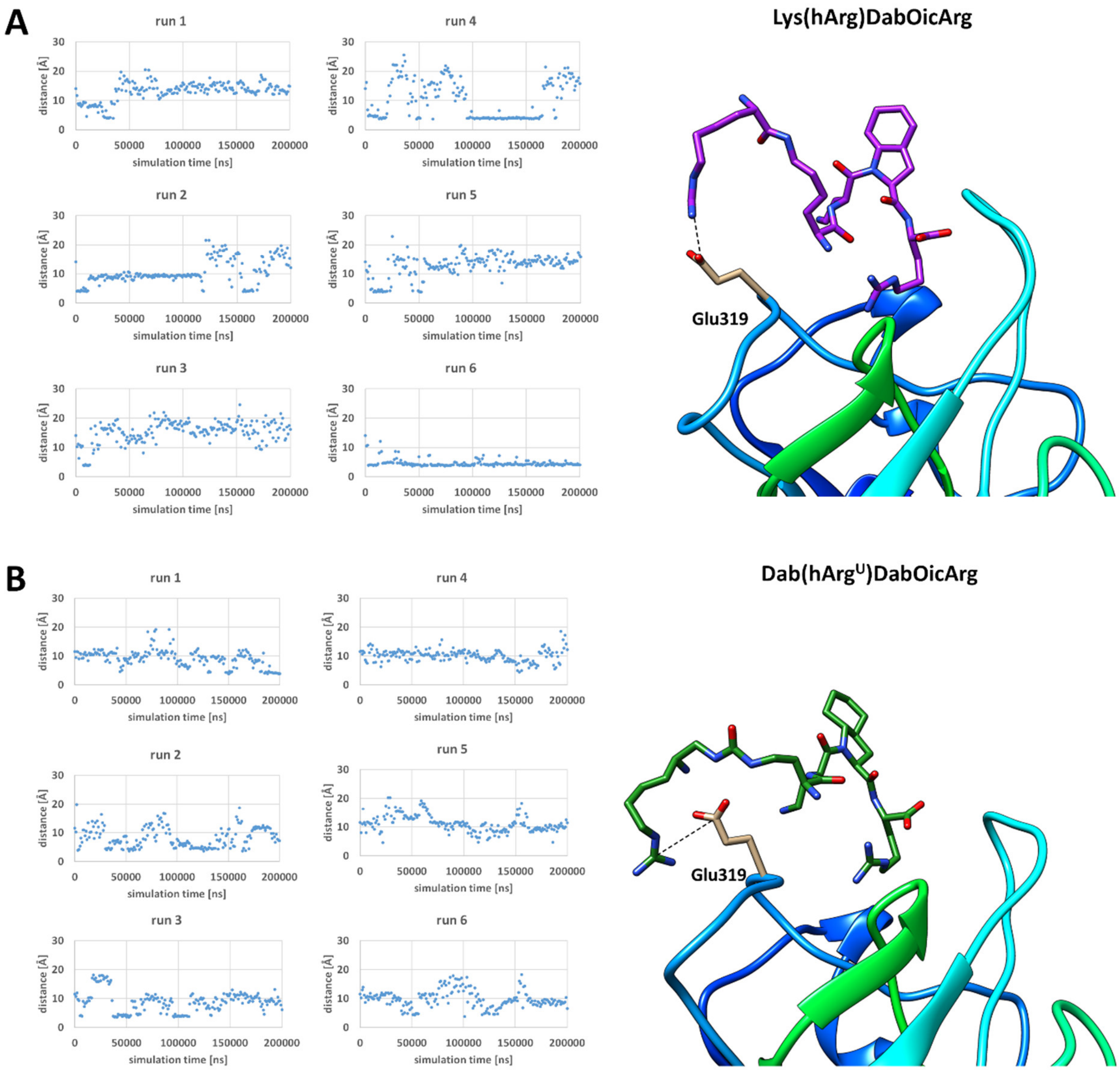

Figure S62. Time evolution of distances between Cδ of Glu319 and Cη of *h*Arg residue in **A)** parent peptide and **B)** compound **6**.

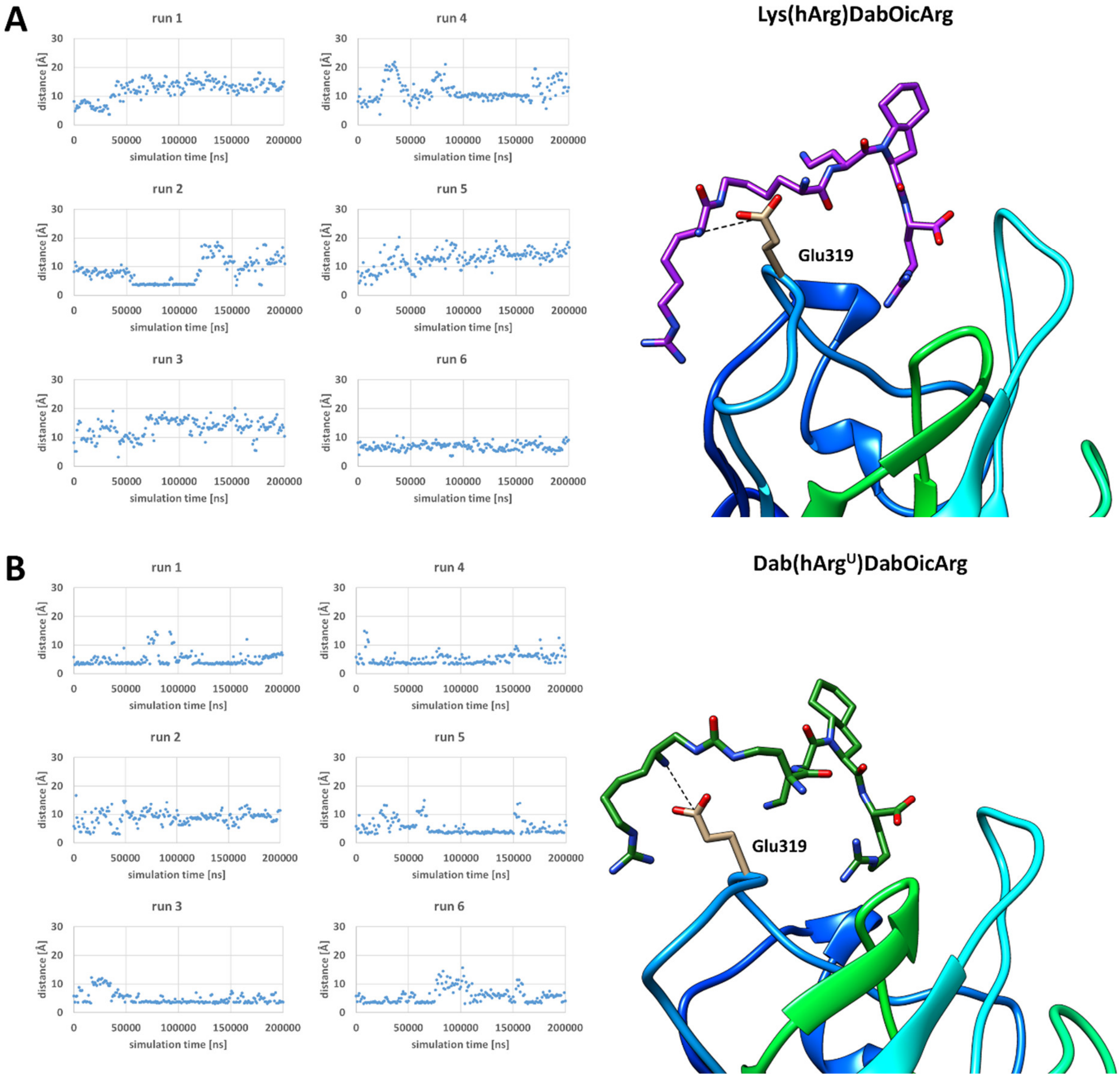

Figure S63. Time evolution of distances between Cδ of Glu319 and Nα of *h*Arg residue in **A)** parent peptide and **B)** compound **6**.

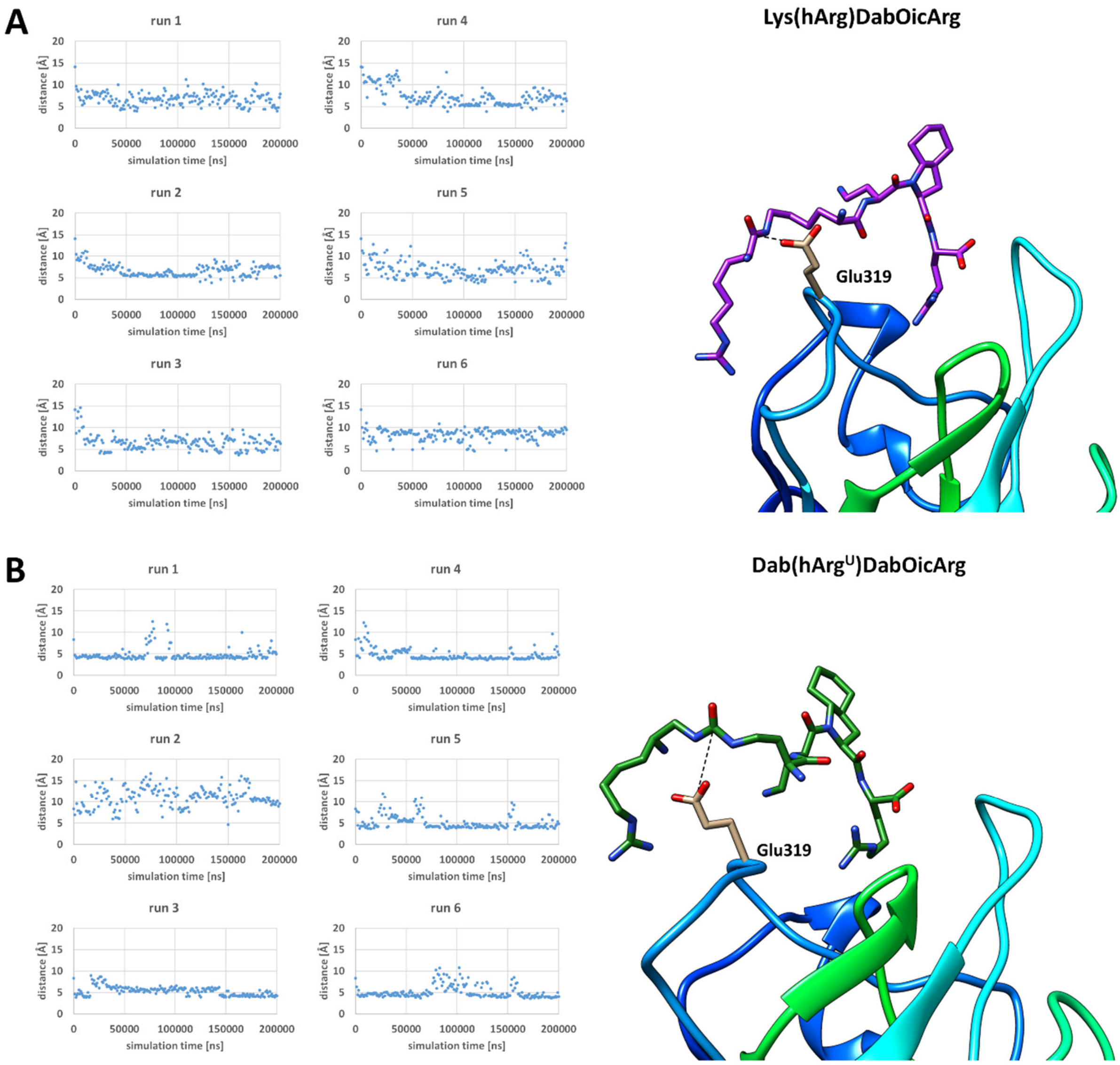

Figure S64. Time evolution of distances between Cδ of Glu319 and CO of *h*Arg residue in **A)** parent peptide and **B)** compound **6**.

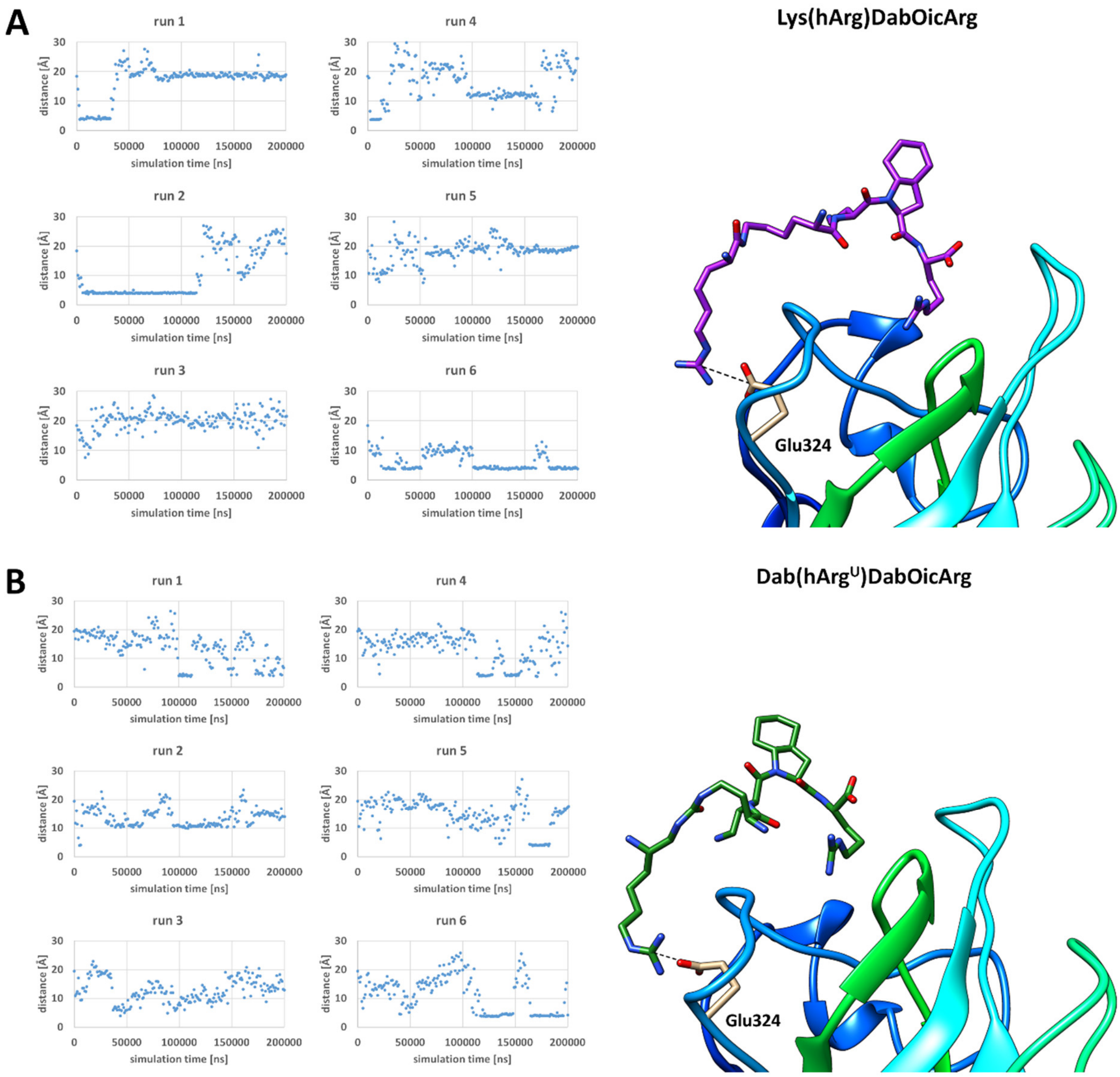

Figure S65. Time evolution of distances between Cδ of Glu324 and Cη of *h*Arg residue residue in **A)** parent peptide and **B)** compound **6**.

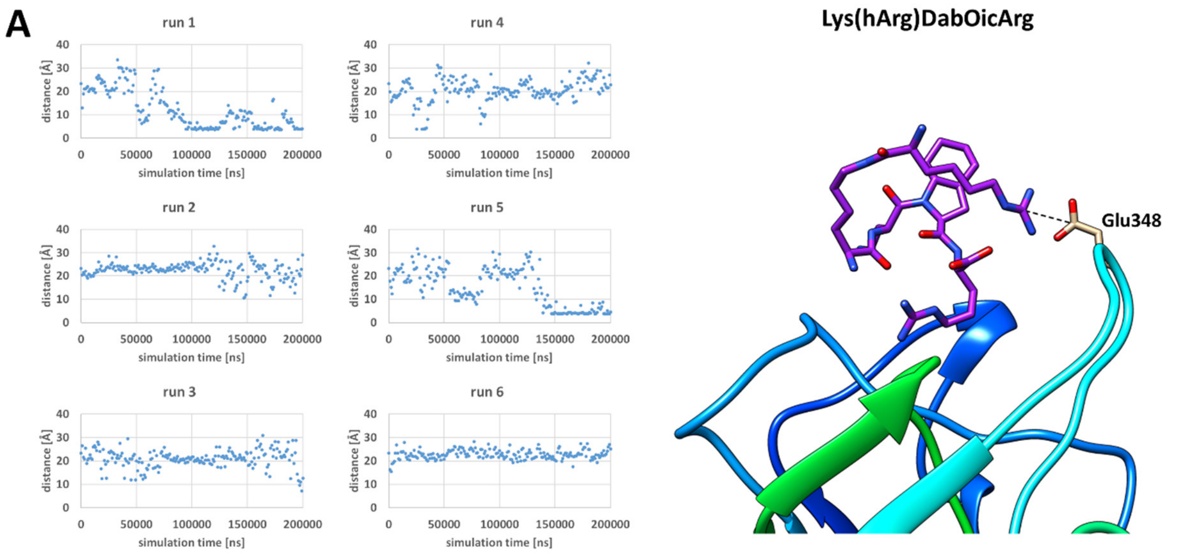

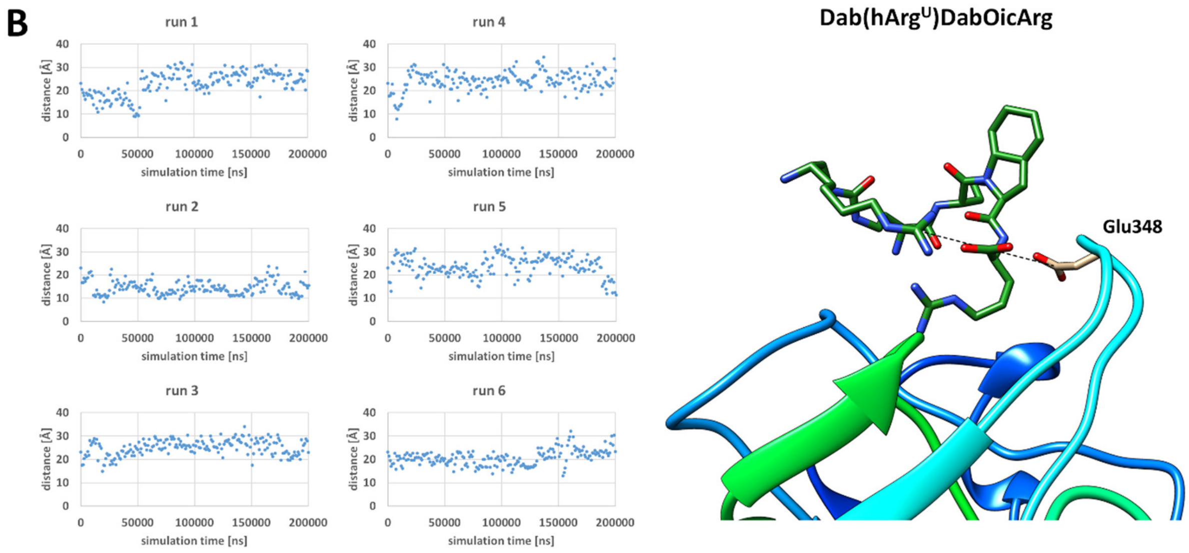

Figure S66. Time evolution of distances between Cδ of Glu348 and Cη of *h*Arg residue in **A)** parent peptide and **B)** compound **6**.

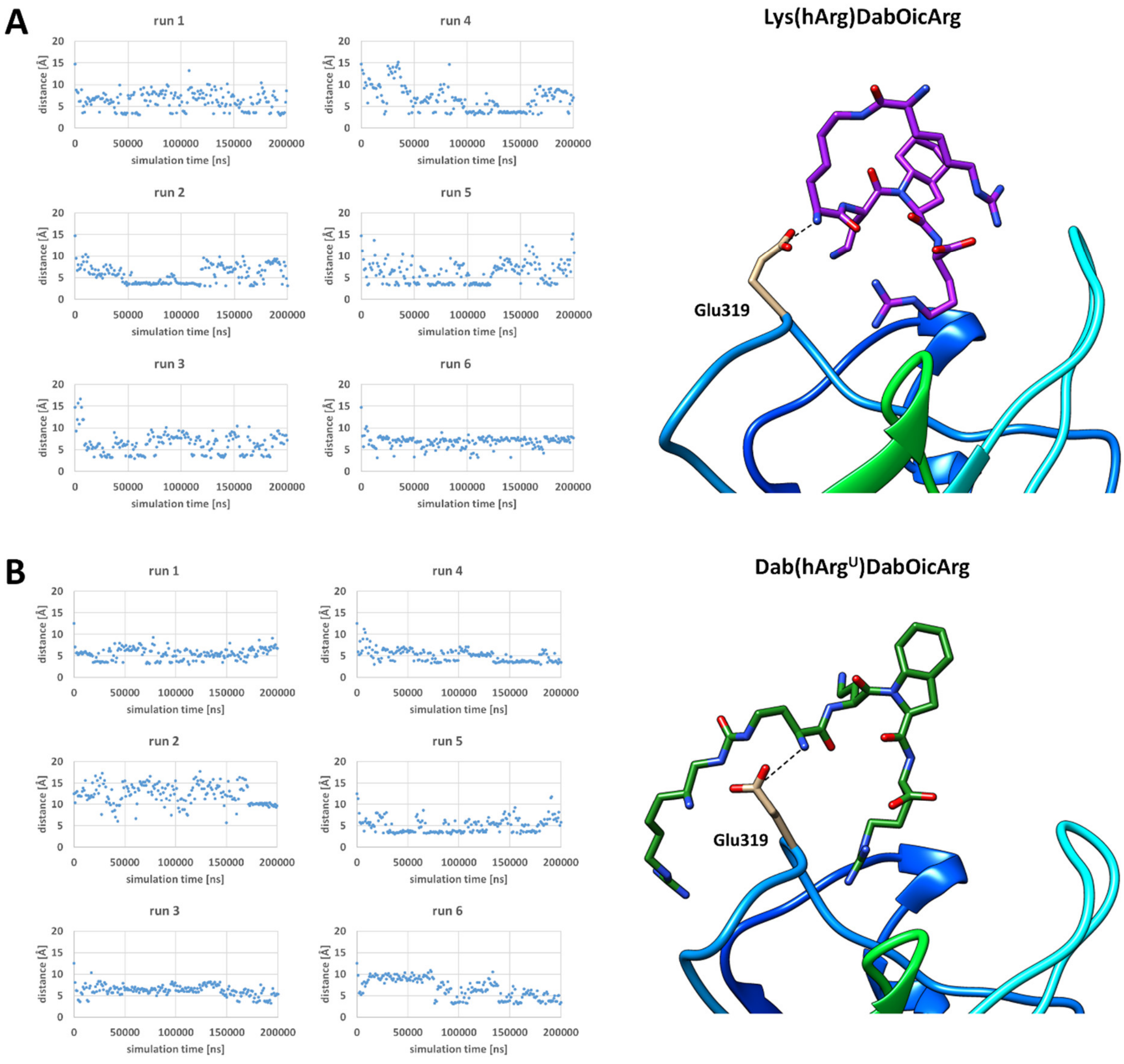

Figure S67. Time evolution of distances between Cδ of Glu319 and Nα of Lys/Dab (P1) residue in **A)** parent peptide and **B)** compound **6**.

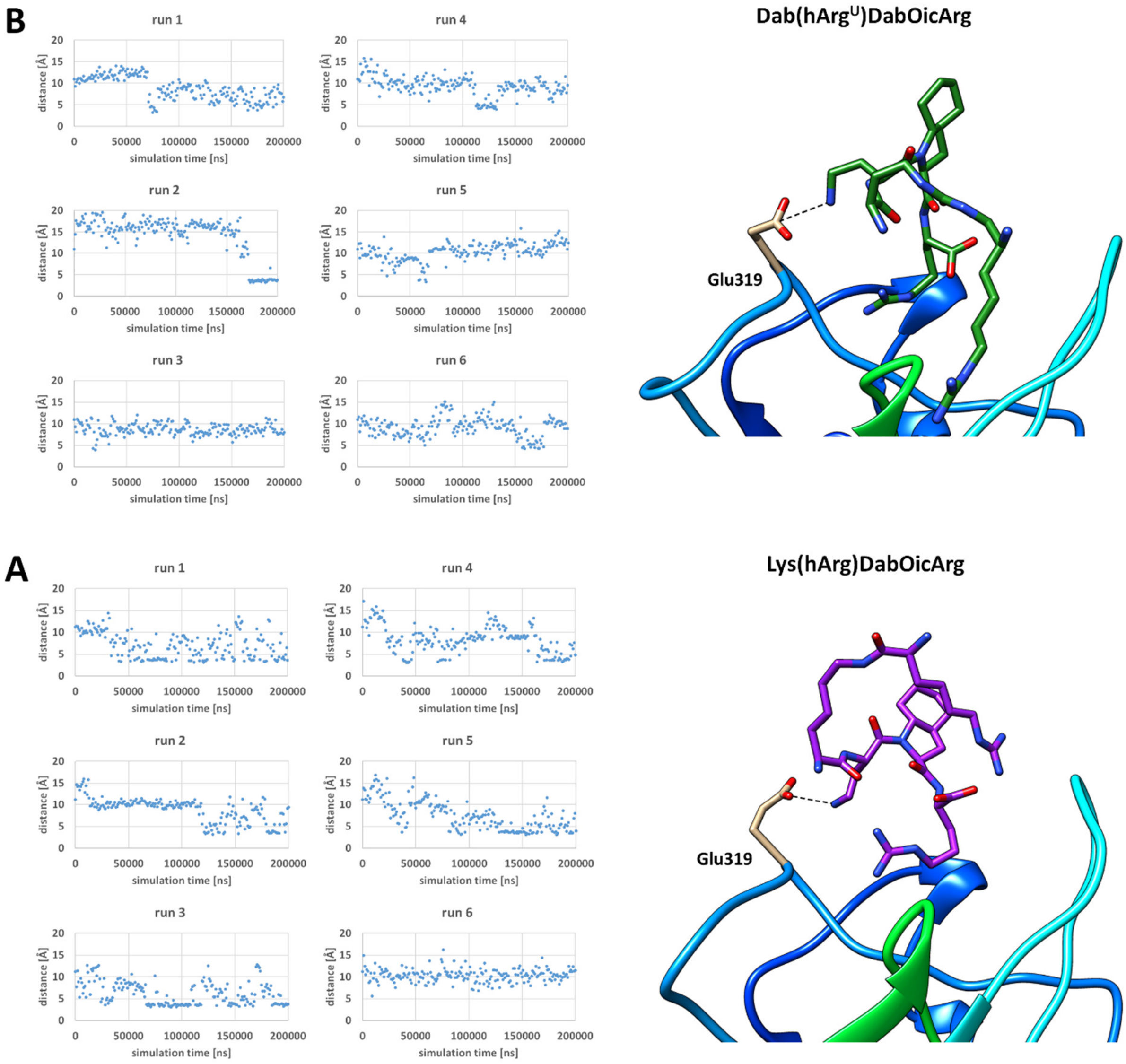

Figure S68. Time evolution of distances between Cδ of Glu319and Nδ of Dab (P2) residue in **A)** parent peptide and **B)** compound **6**.

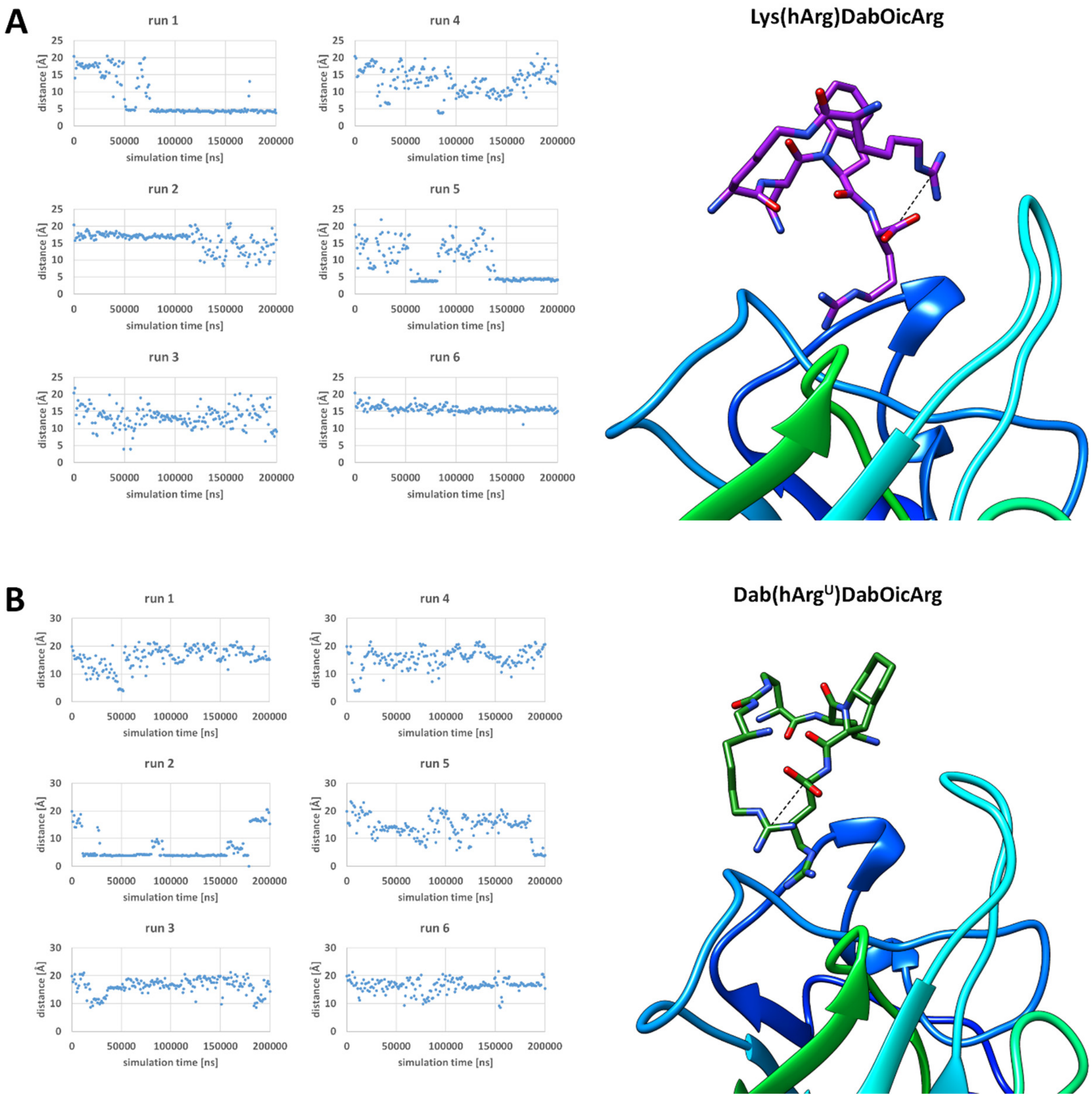

Figure S69. Time evolution of distances between CO of Arg and Cη of *h*Arg residue in **A)** parent peptide and **B)** compound **6**.

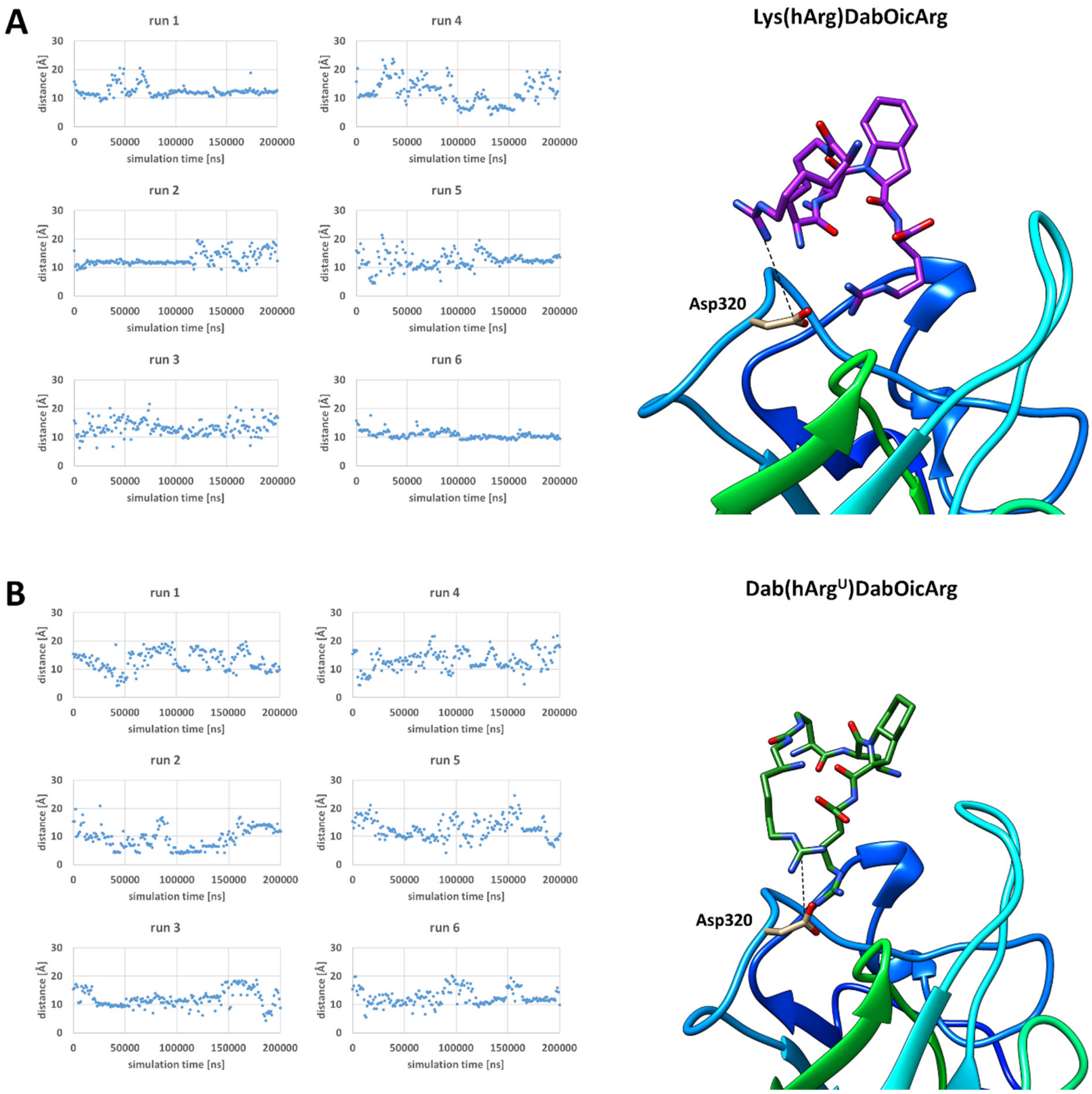

Figure S70. Time evolution of distances between Cγ of Asp320 and Cη of *h*Arg residue in **A)** parent peptide and **B)** compound **6**.

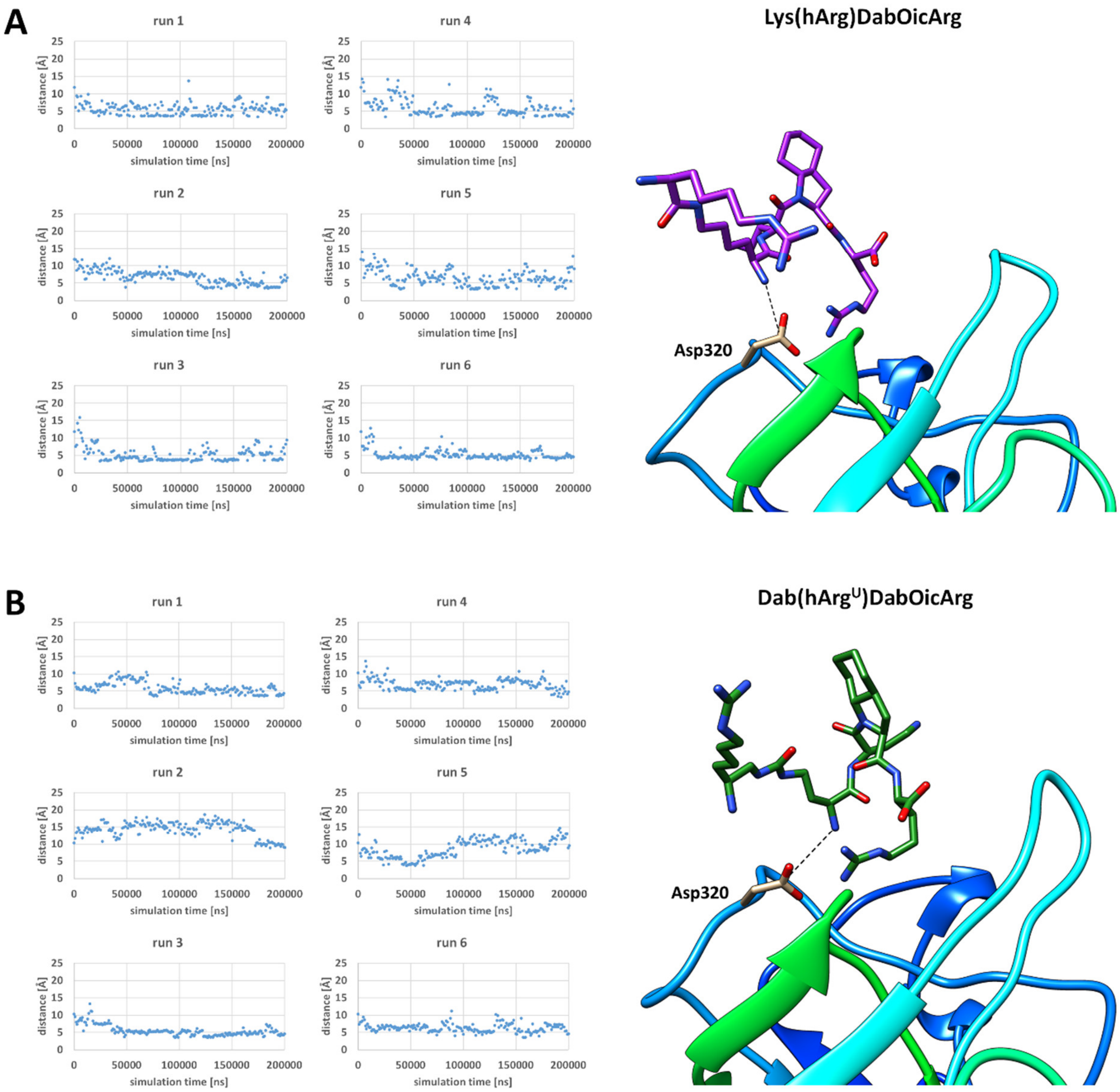

Figure S71. Time evolution of distances between Cγ of Asp320 and Nα of Lys/Dab (P1) residue in **A)** parent peptide and **B)** compound **6**.

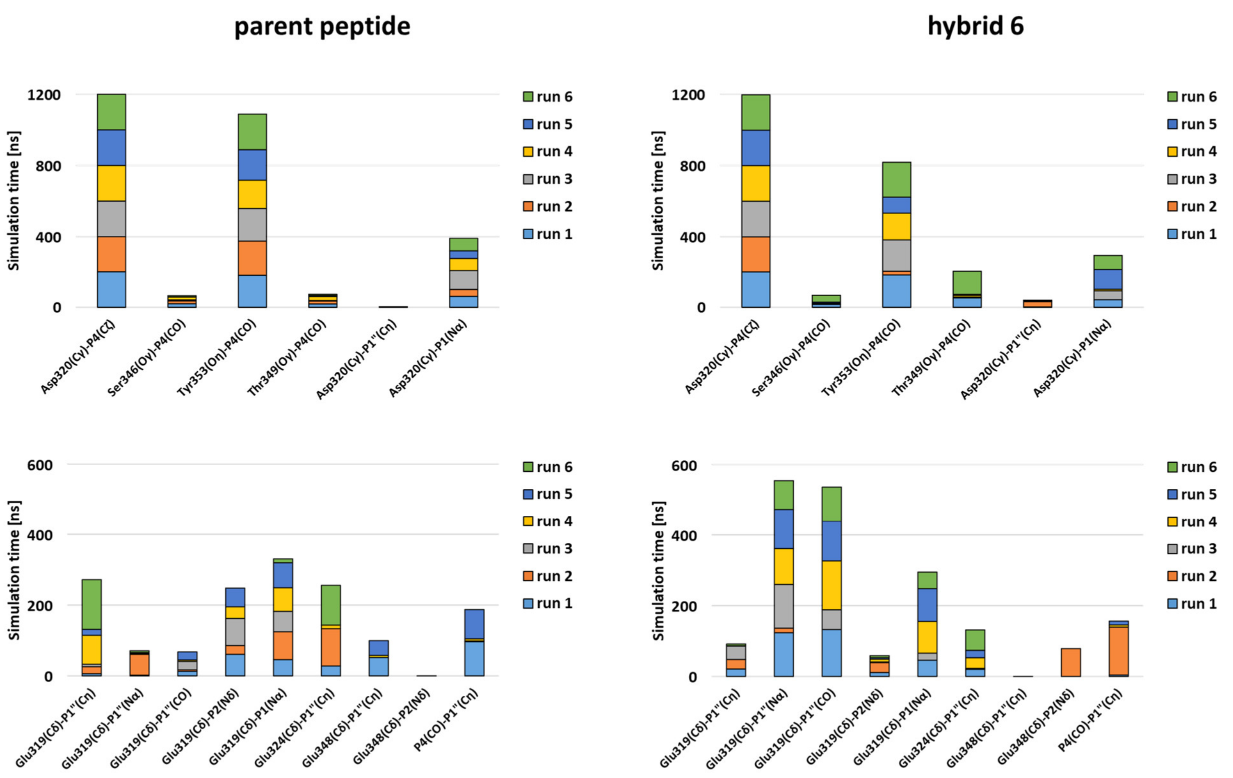

Figure S72. Interactions between NRP-1 residues and ligands (parent peptide and hybrid **6**) functional groups represented as contacts between selected atoms and total time they occurred. Colour difference represents individual runs.

11. References

1. Douat-Casassus, C.; Pulka, K.; Claudon, P.; Guichard, G. Microwave-Enhanced Solid-Phase Synthesis of N,N′-Linked Aliphatic Oligoureas and Related Hybrids. *Org. Lett.* **2012**, *14*, 3130–3133. doi.org/10.1021/ol3012106

2. Puszko, A. K.; Sosnowski, P.; Pułka-Ziach, K.; Hermine, O.; Hopfgartner, G.; Lepelletier, Y.; Misicka, A. Urea Moiety as Amide Bond Mimetic in Peptide-like Inhibitors of VEGF-A_{165}/NRP-1 Complex. *Bioorganic Med. Chem. Lett.* **2019**, *29*, 2493-2497. doi.org/10.1016/j.bmcl.2019.07.016

3. Collie, G.W.; Pulka-Ziach, K.; Lombardo, C.M.; Fremaux, J.; Rosu, F.; Decossas, M.; Mauran' L.; Lambert, O.; Gabelica, V.; Mackereth, C.D.; Guichard, G. Shaping quaternary assemblies of water-soluble non-peptide helical foldamers by sequence manipulation. *Nat Chem.* **2015**, *7*, 871-8. doi: 10.1038/nchem.2353
